# Genome-wide association study of soybean (*Glycine max* [L.] Merr.) germplasm for dissecting the quantitative trait nucleotides and candidate genes underlying yield-related traits

**DOI:** 10.3389/fpls.2023.1229495

**Published:** 2023-08-11

**Authors:** Reena Rani, Ghulam Raza, Hamza Ashfaq, Muhammad Rizwan, Muhammad Khuram Razzaq, Muhammad Qandeel Waheed, Hussein Shimelis, Allah Ditta Babar, Muhammad Arif

**Affiliations:** ^1^ Agricultural Biotechnology Division, National Institute for Biotechnology and Genetic Engineering (NIBGE), Constituent College Pakistan Institute of Engineering and Applied Sciences (PIEAS), Faisalabad, Pakistan; ^2^ Plant Breeding and Genetics Division, Nuclear Institute of Agriculture (NIA), Tando Jam, Pakistan; ^3^ Soybean Research Institute, National Center for Soybean Improvement, Nanjing Agricultural University, Nanjing, China; ^4^ Plant Breeding and Genetics Division, Nuclear Institute for Agriculture and Biology (NIAB), Constituent College Pakistan Institute of Engineering and Applied Sciences (PIEAS), Faisalabad, Pakistan; ^5^ School of Agricultural, Earth and Environmental Sciences, African Centre for Crop Improvement, University of KwaZulu-Natal, Pietermaritzburg, South Africa

**Keywords:** soybean, single nucleotide polymorphism, GWAS, gene ontology, candidate gene discovery

## Abstract

Soybean (*Glycine max* [L.] Merr.) is one of the most significant crops in the world in terms of oil and protein. Owing to the rising demand for soybean products, there is an increasing need for improved varieties for more productive farming. However, complex correlation patterns among quantitative traits along with genetic interactions pose a challenge for soybean breeding. Association studies play an important role in the identification of accession with useful alleles by locating genomic sites associated with the phenotype in germplasm collections. In the present study, a genome-wide association study was carried out for seven agronomic and yield-related traits. A field experiment was conducted in 2015/2016 at two locations that include 155 diverse soybean germplasm. These germplasms were genotyped using SoySNP50K Illumina Infinium Bead-Chip. A total of 51 markers were identified for node number, plant height, pods per plant, seeds per plant, seed weight per plant, hundred-grain weight, and total yield using a multi-locus linear mixed model (MLMM) in FarmCPU. Among these significant SNPs, 18 were putative novel QTNs, while 33 co-localized with previously reported QTLs. A total of 2,356 genes were found in 250 kb upstream and downstream of significant SNPs, of which 17 genes were functional and the rest were hypothetical proteins. These 17 candidate genes were located in the region of 14 QTNs, of which ss715580365, ss715608427, ss715632502, and ss715620131 are novel QTNs for PH, PPP, SDPP, and TY respectively. Four candidate genes, *Glyma.01g199200*, *Glyma.10g065700*, *Glyma.18g297900*, and *Glyma.14g009900*, were identified in the vicinity of these novel QTNs, which encode lsd one like 1, Ergosterol biosynthesis ERG4/ERG24 family, HEAT repeat-containing protein, and RbcX2, respectively. Although further experimental validation of these candidate genes is required, several appear to be involved in growth and developmental processes related to the respective agronomic traits when compared with their homologs in *Arabidopsis thaliana*. This study supports the usefulness of association studies and provides valuable data for functional markers and investigating candidate genes within a diverse germplasm collection in future breeding programs.

## 1 Introduction

The human population is rapidly growing and is expected to reach 10 billion in the next 30 years (Hickey et al., 2019). Arable land for agriculture is decreasing, which poses a threat to food and nutritional security due to climate change causing different biotic and abiotic stresses (Dita et al., 2006; Eltaher et al., 2021; Rani et al., 2023a). However, global food security can be met by the cultivation of legume crops, such as soybean (*Glycine max* L. Merr.), which improves soil fertility through nitrogen fixation (Pandey et al., 2016). Soybean consumption is linked to physiological and health benefits, including the reduction of menopausal symptoms, diabetes mellitus, cancer, and the inhibition of cardiovascular illnesses (Messina, 1999; Messina, 2016; Karikari et al., 2020). However, the overall production of soybean is lagging in many underdeveloped nations, including Pakistan, and this presents a significant issue. Therefore, the per-unit yield of soybeans must be substantially increased. Given that the conditions in Pakistan are extremely beneficial for crop development, the country’s soybean breeding program has recently concentrated on introducing soybean varieties with high grain yields. Diverse genetic resources provide plant breeders with a better chance of creating new improved cultivars with desirable traits (Rani et al., 2023b). Identification of genomic regions associated with yield-attributing traits will help to improve the yield potential of soybean.

Seed weight is an important factor in determining soybean production, seed consumption, and evolutionary fitness (Cui et al., 2004; Gandhi, 2009; Li et al., 2019). To select cultivars with a variety of end uses, soybean breeders must generate a large variability in seed weight. In some particular edamame types (accessions), the soybean hundred seed weight can reach as high as 60 g, whereas in wild types (*Glycine soja Sieb*. et *Zucc*.) it does not exceed 1 g. Therefore, the domestication of soybeans also focused on improving seed weight (Lee et al., 2011; Zhou et al., 2015; Han et al., 2016; Wang et al., 2016). Seed weight is regarded as a complex quantitative trait controlled by a large number of important genes and loci, as well several undetectable loci with minimal impacts; as a result, these polygenes interact with the environment. SoyBase (www.soybase.org) contains more than 300 quantitative trait loci (QTLs) for seed weight. However, it is challenging to utilize these QTLs in breeding programs due to the higher confidence interval and lower genetic variation of linkage mapping data (Gupta et al., 2005). Therefore, linkage disequilibrium-based marker-trait association has been used to take advantage of all recombination events occurring in a natural population (Asins, 2002; Rafalski, 2002).

Genome-wide association study (GWAS) is one of the promising approaches for identifying genetic variations responsible for particular traits (Contreras-Soto et al., 2017). Although GWAS is still a relatively new approach in the fields of molecular biology and plant breeding, it has been widely used in crops such as *Capsicum*, maize, *Sorghum*, and soybean (Wang et al., 2012; Morris et al., 2013; Zhang et al., 2015b; Contreras-Soto et al., 2017; Han et al., 2018). According to reports, GWAS is more accurate than well-established methods, such as bi-parental QTL mapping, at identifying candidate genes (Qi et al., 2020). For instance, Miao et al. (2020) recently identified GmSWEET39 (*Glyma.15 g049200*/*Glyma15g05470*) utilizing regional association mapping for seed oil. When this gene was overexpressed in *Arabidopsis*, the quantity of seed oil rose by at least 10%. On all 20 chromosomes of soybean, many QTNs have been discovered and reported through GWAS (Sun et al., 2012; Chaudhary et al., 2015; Zhang et al., 2015a; Wang et al., 2016; Zhang et al., 2016; Fang et al., 2017; Yan et al., 2017b; Copley et al., 2018; Wen et al., 2018; Assefa et al., 2019; Jiang et al., 2019; Zhao et al., 2019). However, population type, size, and the GWAS approach can all lead to differing mapping results. Single-marker genome-wide scan models, such as the mixed linear model (MLM) and general linear model (GLM), are most frequently used for mapping loci related to seed weight in soybean (Wen et al., 2018). The problem of multiple test correction for the threshold significant value as well as mapping power are a couple of the drawbacks of these models. Different multi-locus models, like those for soybean, have been developed and employed in recent GWAS studies.

Population structure, kinship, and the level of linkage disequilibrium (LD) have the greatest effects on the precision and effectiveness of QTLs discovered by GWAS (Neale and Savolainen, 2004; Weir, 2010; Korte and Farlow, 2013). However, biasness in GWAS created by the aforementioned factors can be removed by adjusting the false discovery rate (FDR), via modifications to the model, and the use of population structure matrices and modified kinship (Kang et al., 2008; Vanraden, 2008; Wang et al., 2012; Li et al., 2013; Brzyski et al., 2017). Such modifications in GWAS designs can lead to more accurate identification of significant marker-trait associations, which is reinforced by more recent improvements in computational approaches (Takeuchi et al., 2013; Tang et al., 2016; Kichaev et al., 2019; Qi et al., 2020). The use of bioinformatics techniques has increased the possible identification of potential genes for target QTL. One such methods is to use a co-expression network, which gives genes with similar functions priority. Numerous crops, including maize (Schaefer et al., 2018), rice (Sarkar et al., 2014), peanuts (Zhang et al., 2019), *Arabidopsis* (Angelovici et al., 2017), and soybean (Wu et al., 2019; Yang et al., 2019; Razzaq et al., 2023), have effectively benefited from its application. Through the incorporation of co-expression network analysis, Palumbo et al. (2014) found a class of hub genes that result in considerable transcriptome reprogramming throughout grapevine development. Hub genes (genes with strong connectivity) may provide information about the function of a gene in the network (Das et al., 2017).

The current study was conducted using genotypes from the USDA-ARS with the aim of identifying molecular markers and candidate genes that are related to yield and other important agronomic traits using GWAS. To our knowledge, this is the first study to describe the identification of genetic factors regulating grain yield, as well as high-performing genotypes, in a Pakistani environment.

## 2 Materials and methods

### 2.1 Plant material and phenotyping

**Figure 1:**
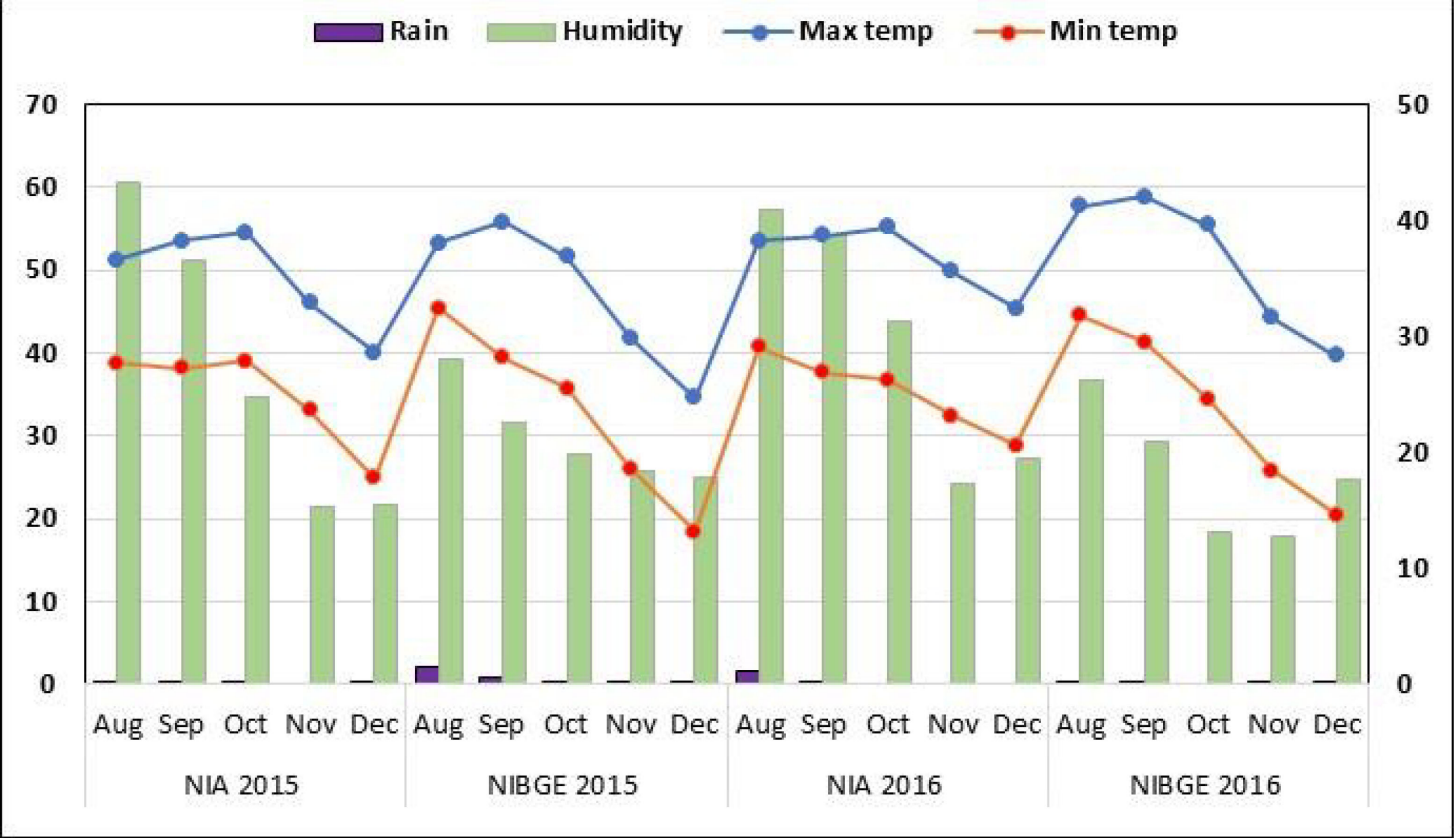
Weather conditions for the soybean genotypes growth period during 2015 and 2016. Monthly rainfall (mm) (left x-axis) and relative humidity (%) (right x-axis).

A total of 155 soybean accessions were collected from the USDA-ARS germplasm collection center (**Supplementary Table 1**). All plant materials were planted at two locations: the National Institute for Biotechnology and Genetic Engineering, Faisalabad (31°’42’N 73°’02’E), and the Nuclear Institute of Agriculture, Tando Jam (25°’60’N 68°’60’E), during August 2015/2016. A field experiment was conducted using a single-row plot randomized complete block design with three repetitions for the tested conditions in four environments (two locations × 2 years). Seedbeds were prepared by ploughing once with a cultivator, then planking and ploughing twice with a rotavator. Sowing was carried out with the use of a dibbler to keep a spacing of 3 inches between plants. For appropriate emergence, a row-to-row gap of 30 cm and a seed depth of 1–2 inches were maintained. For each soybean accession, three 2.43m rows were used. Weather conditions, including temperature, rainfall, and humidity, during the growing period in 2015/2016 at both locations were obtained from https://www.worldweatheronline.com/(**Figure 1**).

Plants from each row were randomly chosen to record phenotypic data at full maturity in the years 2015 and 2016. Plant height (PH) was measured from above the surface of the soil to the tip of the main stem. The number of nodes (NN) were counted on the main stem of each plant. Likewise, pods per plant (PPP) were counted on each plant. Seeds per plant (SDPP) were measured by counting the number of seeds on each plant. Seed weight per plant (SWPP) was measured by calculating the weight of all the seeds harvested from a single plant. For hundred grain weight (HGW), 100 seeds were selected from each genotype to calculate seed weight. The total yield (TY) of each genotype was calculated on a plot-by-plot basis after harvesting.

### 2.2 Statistical analysis

Combined analysis of variance (ANOVA) was used to estimate the genotype, environment, and genotype environment interaction for 2015/2016. The soybean accessions of the GWAS panel were considered as a fixed effect, whereas environment and block were considered as a random effect. Correlations between PH, NN, PP, SDPP, SWPP, HGW, and TY were observed by using mean data of all the traits in the R package “Performance Analytics” to draw the correlation matrix.

### 2.3 Genotyping and quality control

For genotyping the population, Illumina Infinium SoySNP50K Bead Chip data from the Soybase database (https://www.soybase.org/snps/) was downloaded to enable genotyping of the population using the Illumina Infinium SoySNP50K Bead Chip. A total of 42,291 SNPs were found for the selected genotypes, of which 211 that were found in unanchored sequence scaffolds were eliminated before further investigation. In TASSEL v5.0, the remaining 42,080 SNPs were imported. Monomorphic SNPs, SNPs with more than 20% of the genotype’s data missing, SNPs with more than 10% heterozygosity, and SNPs with a minor allele frequency of less than 5% were removed from the data. Finally, the remaining 35,110 SNPs were employed for the GWAS study and diversity analysis. An SNP density plot was constructed using the R package CMplot.

### 2.4 Population STRUCTURE

Population structures of 155 diverse genotypes were investigated using STRUCTURE 2.3.1 software. The number of subgroups (K) was set from one to 10, with three replications. The length of the burn-in period and number of Monto Carlo Markov chain (MCMC) replication were both set to 10,000 replicates. An admixture model along with a correlated allele frequency model (independent of each run) was used to analyze the population structure (Shi et al., 2016). STRUCTURE HARVESTER was used to estimate the best-suited K in this population.

### 2.5 Genome wide association study (GWAS)

Fixed and random model Circulating Probability (FarmCPU) implemented in the R package was used for GWAS. The FarmCPU model incorporates significant markers as covariates in a stepwise regression model MLM and uses a multiple locus linear mixed model (MLMM) to largely minimize the confusion between tested markers and kinship (Liu et al., 2016). Average data of all the traits in each year was used as phenotype, whereas 35,110 SNPs obtained from the 50K SNP chip from SoyBase were taken as genotype for GWAS analysis. The SNPs associated with traits with P ≤ 1.2 × 10^-4^ (-log_10_P _=_ 3.92) were identified as significant SNPs.

### 2.6 Linkage disequilibrium

By using TASSEL 5.0 software, pairwise LD between the markers was estimated using the squared coefficient (r^2^) of alleles. Average r^2^ dropped to half of its maximum value when the decay rate of LD was plotted as the chromosomal distance between markers. The critical value of r^2^ beyond which LD was likely to be caused by linkage was set at r^2^_=_ 0.1.

### 2.7 Candidate gene discovery

The putative genes underlying the ±250 Kb genomic region of significant SNPs were searched using *G.max* Williams 82.a2 as the reference genome in SoyBase (https://www.soybase.org/snps/). Additionally, functional annotation of each gene was investigated using SoyBase to find the potential candidate genes. The following criteria were used to choose candidate genes: (i) genes with known functions in soybean associated with a trait of interest; (ii) genes located by significant SNPs; and (iii) genes with known functions in *Arabidopsis* orthologs associated with the desired trait. The enrichment of Gene Ontology (GO) terms was calculated by comparing all the genes included in each QTN to the number of genes annotated in each GO term using ShinyGO 0.76 web software (Ge et al., 2020). For the identified genes, enrichment analysis was performed to check whether the set is enriched with the genes of a certain pathway or functional category. Genes annotated in the interval were compared with their orthologs in other plant species using The *Arabidopsis* Information Resource (TAIR). The validity of potential candidate genes was then investigated in the literature.

## 3 Results

### 3.1 Statistical analysis

**Figure 2:**
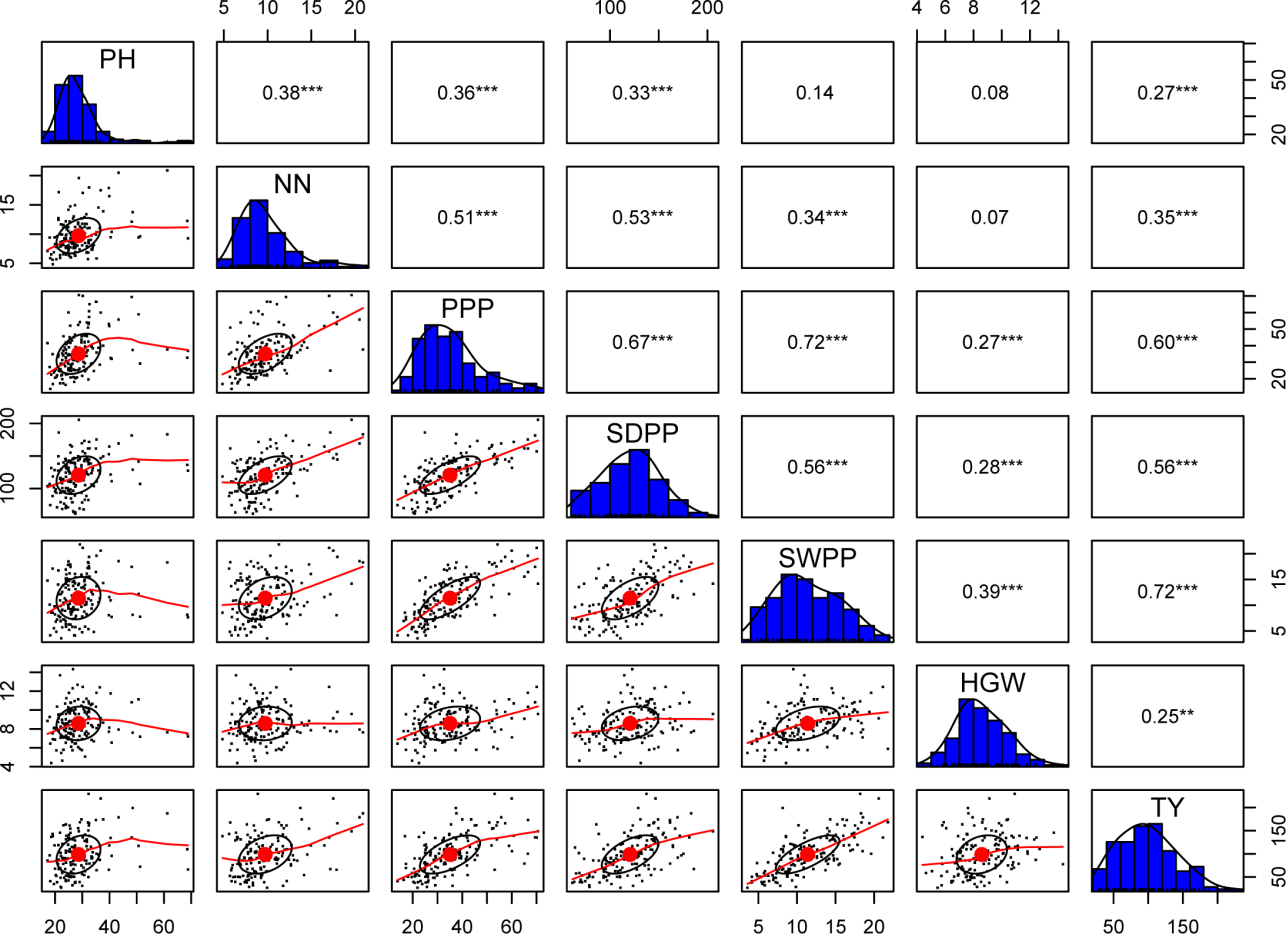
Correlation analysis of 155 soybean accessions between seven traits: plant height (PH), number of nodes (NN), pods per plant (PPP), seed per plant (SDPP), seed weight per plant (SWPP), hundred grain weight (HGW), and total yield (TY). One star ('*'), Two stars ('**'), Three stars ('***') denote that the corresponding variable is significant at 10%, 5%, 1% level, respectively. Absence of star denotes no significant variable.

**Figure 3:**
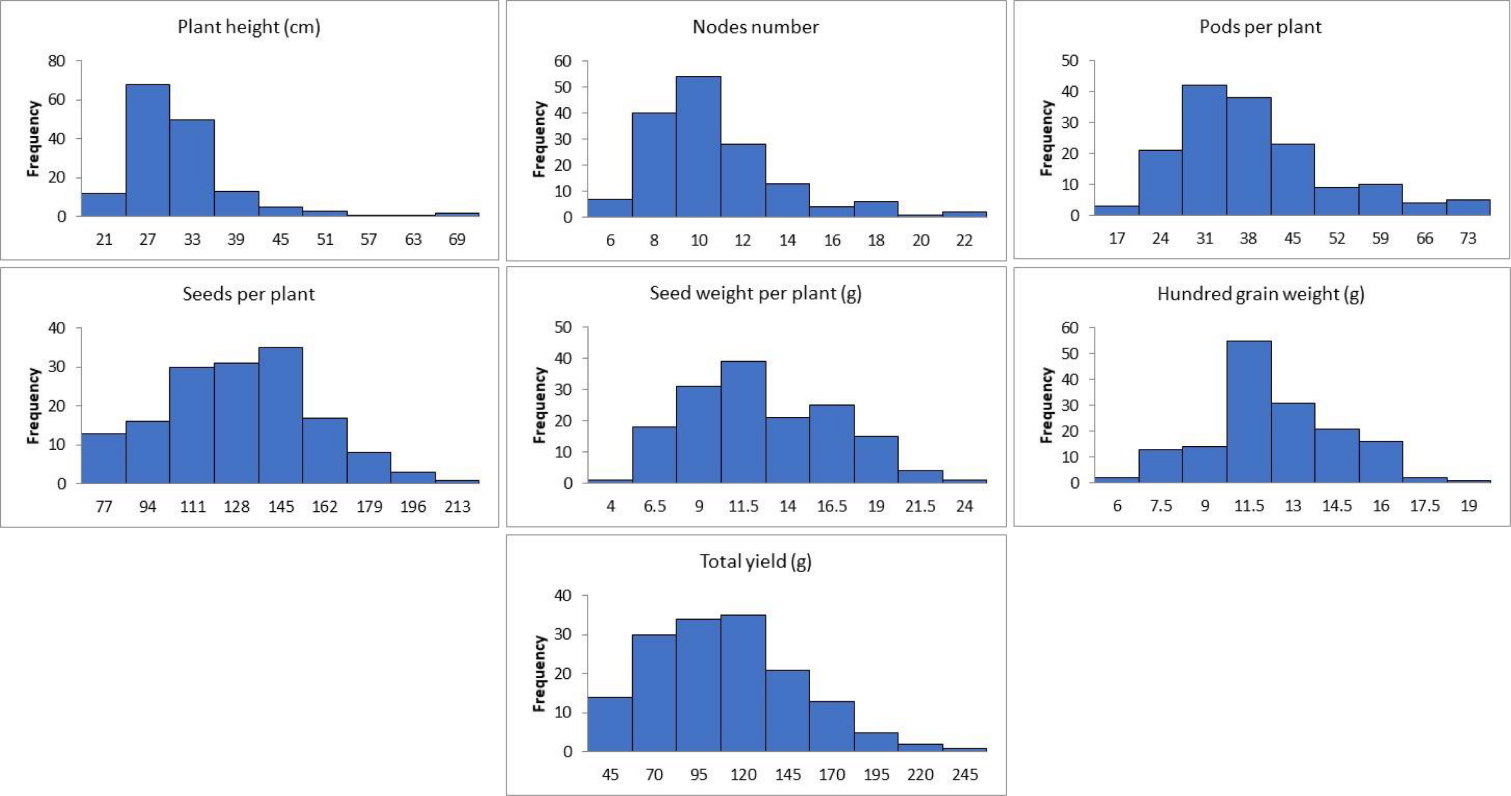
Frequency distribution of the mean data of agronomic traits of soybean accessions across the environments.

The results obtained from combined ANOVA showed that environment was the main influence on all the traits except hundred grain weight, which is mainly influenced by G × E interaction, i.e., 39% (**Table 1**). A correlation matrix of average data for PH, NN, PPP, SDPP, SWPP, HGW, and TY showed that the traits were positively correlated (**Figure 2**). TY showed a high level of positive correlation with PPP, SDPP, and SWPP but a low level of positive correlation with PH, NN, and HGW. PH and HGW showed a low level of positive correlation with all the traits. The correlation observed for PPP was positive but high with NN, SDPP, SWPP, and TY and slightly low with PH and HGW. SDPP showed a moderate positive correlation with SWPP and TY. The correlation observed for HGW was positive but low with all the traits. The frequency distributions of the phenotypic data for the quantitative characteristics PH, NN, PPP, SDPP, SWPP, HGW, and TY revealed a continuous distribution (**Figure 3**).

**Table 1:** Combined analysis of variance (ANOVA) for soybean yield and yield components.

Traits	Source	Df	SS	V%	MS	F value
Plant height	Environment (E)	3	6,099,902	39	2,033,301**	369.35
	Genotype (G)	154	4,521,32	3	2,935.92ns	0.53
	G × E	462	2,170,654	14	4,698.38ns	0.85
	Residuals	1,232	6,782,094	44	5,504.94	
	Total	1,851	1,550,478,1	100	2,046,440	
	CV%	58.02				
Nodes number	Environment (E)	3	5,757,462	58	1,919,154**	765.64
	Genotype (G)	154	1,584,99.4	2	1,029.21ns	0.41
	G × E	462	8,456,36.3	9	1,830.38ns	0.73
	Residuals	1,232	3,088,105	31	2,506.57	
	Total	1,851	9,849,702	100	1,924,520	
	CV%	53.7				
Pods per plant	Environment (E)	3	2,680,701	46	893,566.9**	380.32
	Genotype (G)	154	1,863,21.6	3	1,209.88ns	0.51
	G × E	462	5,207,3.2	1	112.71ns	0.04
	Residuals	1,232	2,894,548	50	2,349.47	
	Total	1,851	5,813,643	100	8,972,38.9	
	CV%	11.9				
Seeds per plant	Environment (E)	3	1,314,069	23	4,380,23**	143.19
	Genotype (G)	154	3,405,77.9	6	2,211.54ns	0.72
	G × E	462	3,517,38.8	6	761.33ns	0.24
	Residuals	1,232	3,768,500	65	3,058.84	
	Total	1,851	5,774,885	100	4,440,54.8	
	CV%	11.39				
Seed weight per plant	Environment (E)	3	3,961,02.2	15	1,320,34.1**	80.06
	Genotype (G)	154	6,127,6.65	2	397.9ns	0.24
	G × E	462	2,077,66.2	8	449.71ns	0.27
	Residuals	1,232	2,031,703	75	1,649.1	
	Total	1,851	2,696,848	100	1,345,30.8	
	CV%	291.74				
Hundred grain weight	Environment (E)	3	3,854.25	24	1,284.75**	134,12.69
	Genotype (G)	154	5,647.79	36	36.67**	382.87
	G × E	462	6,250.34	39	13.52**	141.24
	Residuals	1,232	118	1	0.09	
	Total	1,851	1,587,0.38	100	1,335.052	
	CV%	3.61				
Total yield	Environment (E)	3	1,224,584	16	4,081,94.6**	95.11
	Genotype (G)	154	4,079,91.2	5	2,649.29ns	0.61
	G × E	462	8,745,47.7	11	1,892.96ns	0.44
	Residuals	1,232	5,287,293	68	4,291.63	
	Total	1,851	7,794,416	100	4,170,28.5	
	CV%	15.96				

** significant at p ≤ 0.0001; ns, not significant.

### 3.2 Population STRUCTURE and diversity analysis

**Figure 4:**
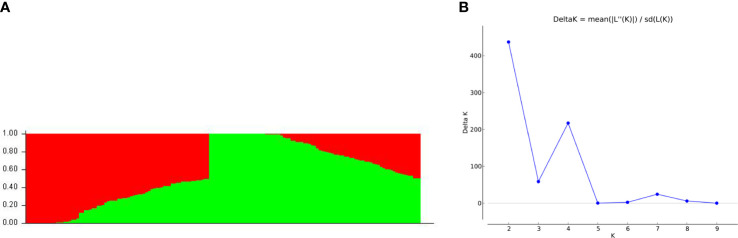
Population STRUCTURE analysis of 155 soybean accessions for genetic diversity analysis. **(A)** Bar plot divides the population into two cluster in which red represents cluster 1 and green represents cluster 2. **(B)** Plot showing the results from STRUCTURE HARVESTER. The delta K peak corresponds to K = 2.

STRUCTURE Harvester revealed a delta K peak at K = 2 (**Figure 4A**), demonstrating the presence of two subpopulations in the panel of 155 soybean natural populations. STRUCTURE 2.3.4 produced a bar plot that displayed two subpopulations with little differentiation but a lot of mixing (**Figure 4B**). Fst values (mean inbreeding coefficients of the subpopulation relative to the overall population) for subpopulation 1 and subpopulation 2 were 0.1101 and 0.4970, respectively (**Supplementary Table 2**). Individuals in the same cluster were separated on average by 0.3508 for subpopulation 1 and 0.2385 for subpopulation 2. (**Supplementary Table 2**). A genotype relating to each cluster was demonstrated in terms of membership proportion, which was found to be 0.4573 and 0.5427 for subpopulation 1 and subpopulation 2, respectively (**Supplementary Table 2**). Among populations, the average allele frequency divergence observed was 0.126 (**Supplementary Table 2**).

### 3.3 Linkage disequilibrium

**Figure 5:**
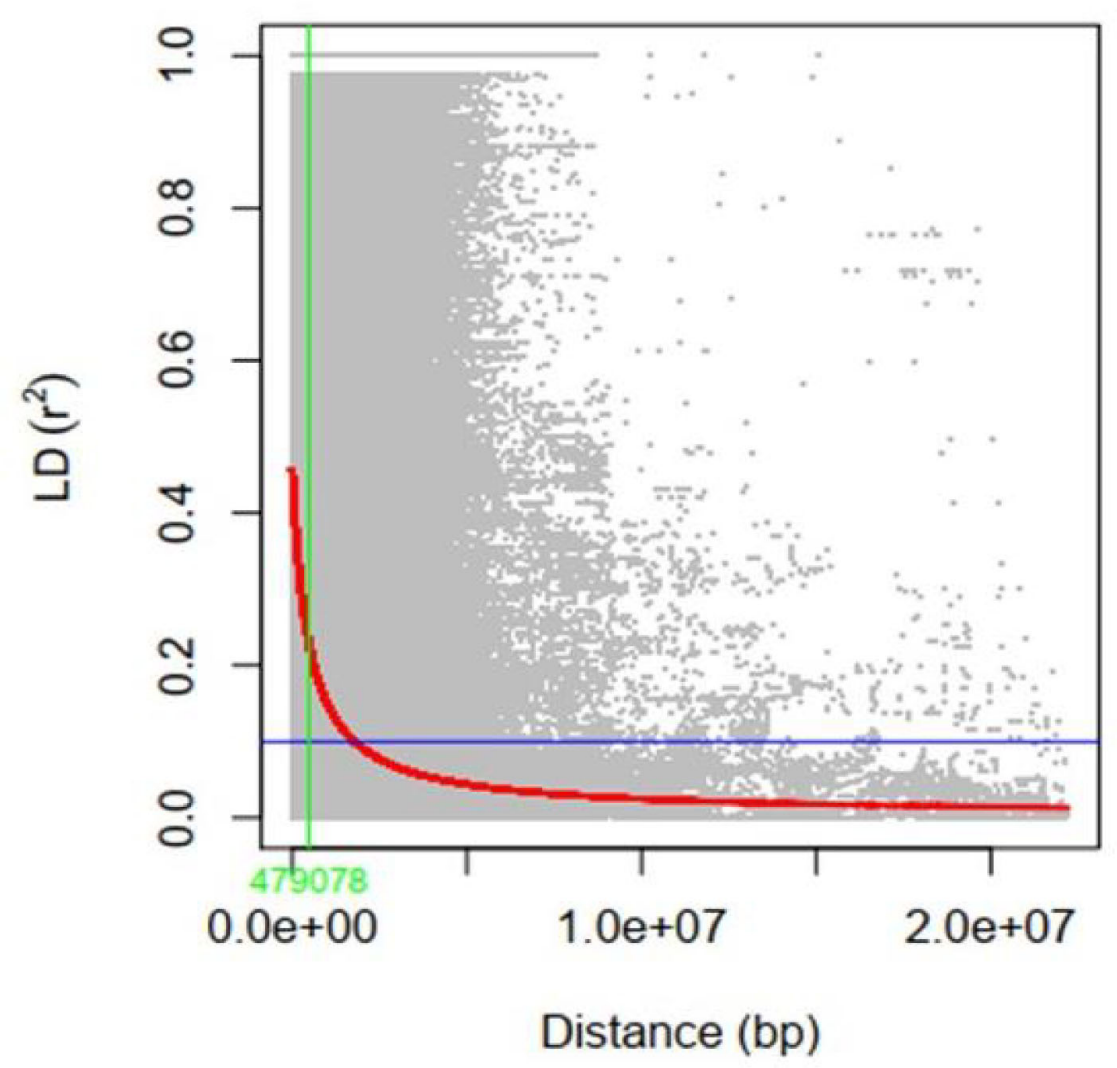
Genome-wide average linkage disequilibrium (LD) decay rate. The x-axis shows the distance (base pair) between SNPs, and the y-axis shows the LD value.

**Figure 6:**
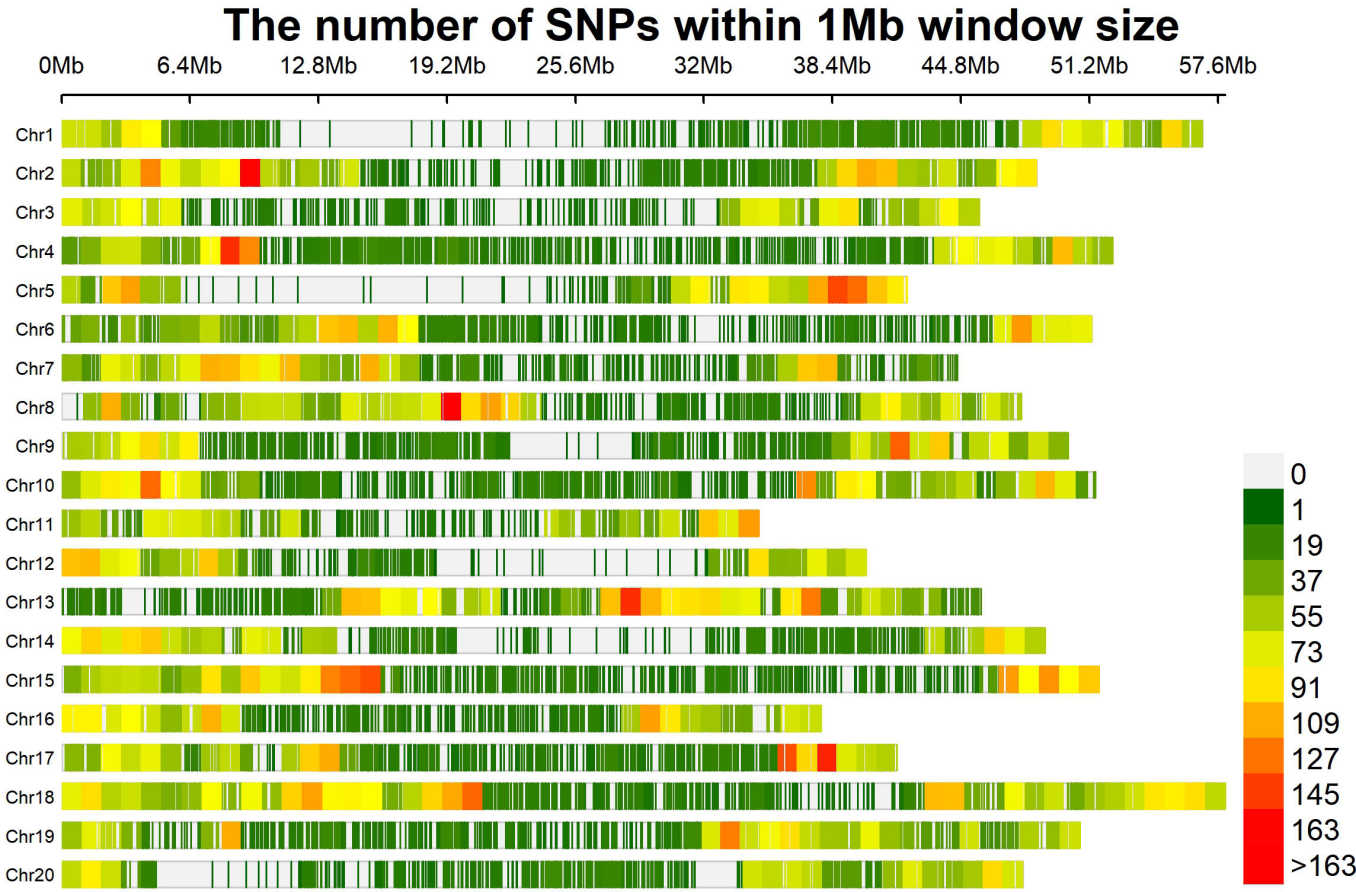
SNP density plot drawn using Cmplot. The horizontal axis shows the chromosome length (Mb); the color bar exhibits the number of SNPs.

To assess the LD decay for the entire genome, 35,110 SNPs were used. The LD decay with increasing physical distance was shown by a scatter plot of r^2^ against physical distance. The average genetic distance at which LD declined below r^2^ of 0.1 was used to calculate the average QTL confidence interval (CI). The whole-genome average maximum r^2^ value was recorded at 0.44, which decayed to 0.22 at a CI of 479,078 bp for the QTLs (**Figure 5**). The average SNP density varied over each chromosome, ranging from 40.57 kb per SNP on chromosome 1 to 20.21 kb per SNP on chromosome 18 (**Supplementary Table 3**; **Figure 6**). A total of 35,110 high-quality SNPs retained after filtering were used for GWAS analysis. SNPs on each chromosome varied from 1,251 (chromosome 12) to 2,868 (chromosome 18), with an average of 1,755 SNPs per chromosome (**Supplementary Table 3**).

### 3.4 GWAS and candidate gene discovery

**Figure 7:**
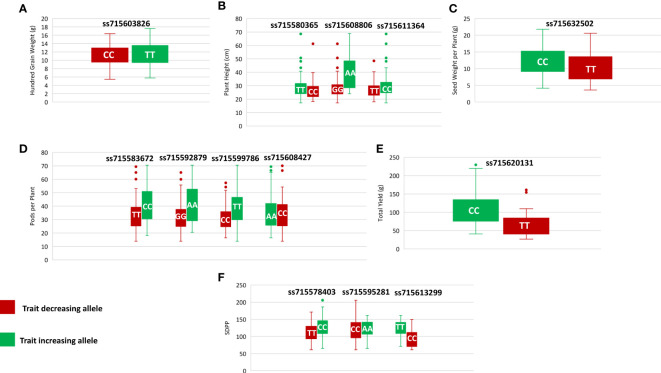
Phenotypic differences between accessions carrying different alleles of significant SNPs for hundred grain weight **(A)**, plant height **(B)**, seed weight per plant **(C)**, pods per plant **(D)**, total yield **(E)**, and seed per plant **(F)**.

**Figure 8:**
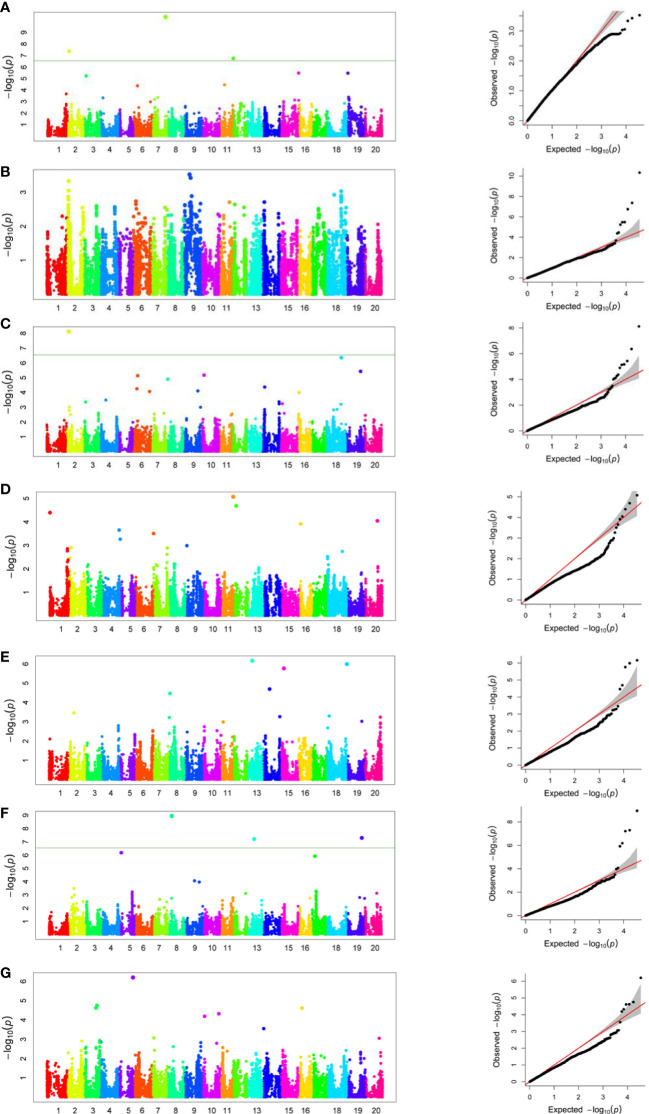
Genome-wide association analysis Manhattan plots and Q-Q plots of 155 soybean accessions for plant height **(A)**, number of nodes **(B)**, pods per plant **(C)**, seeds per plant **(D)**, seed weight per plant **(E)**, hundred grain weight **(F)**, and total yield **(G)**.

Genome-wide association analysis was performed for the grand mean of phenotypic traits of all the environments and 35,100 SNP markers using the FarmCPU, in which P + K values were used as covariates for reducing the FDR. A total of 51 significant SNPs were identified for PH, NN, PPP, SDPP, SWPP, HGW, and TY (**Table 2**). Of these 51 significant SNPs, 18 were putatively novel, whereas the remaining 33 SNPs colocalized with previously reported QTLs. Most of these QTNs have a positive effect on the traits (**Figure 7**). Manhattan plots and associated Q-Q plots are shown in **Figure 8**. A maximum of 12 SNPs were found to be associated with the PPP, and only two SNPs were significantly associated with NN.

**Table 2:** SNPs significantly reported for seven soybean traits along with previously reported QTLs in overlapping regions.

Traits	SNP	Physical position		Significant region		*P* value	Effect	Known QTLs	Reference
		Chr.	Position (bp)	Start position	End position				
HGW	ss715602808	8	8,869,615	8,619,615	9,119,615	1.11E-09	-0.98		
	ss715635170	19	4,224,597,7	4,199,597,7	4,249,597,7	5.07E-08	-0.93	Seed number 2-1, Seed weight 12-3	(Specht et al., 2001; Funatsuki et al., 2005)
	ss715614230	13	1,942,529,9	1,917,529,9	1,967,529,9	6.13E-08	-0.95	Seed weight 42-3, Seed weight 50-14	(Kato et al., 2014)
	ss715589876	5	3,013,606	2,763,606	3,263,606	6.57E-07	-0.63		
	ss715628225	17	7,913,612	7,663,612	8,163,612	1.20E-06	0.89	Seed weight 21-2	(Gai et al., 2007)
	ss715603328	9	2,576,606,5	2,551,606,5	2,601,606,5	8.60E-05	-0.54	Seed weight 50-5	(Kato et al., 2014)
	ss715603826	9	3,941,805,2	3,916,805,2	3,966,805,2	1.07E-04	-0.44	Seed weight 27-3	(Vieira et al., 2006)
	ss715581257	2	1,439,810,0	1,414,810,0	1,464,810,0	3.27E-04	0.56	Seed weight 49-8	(Teng et al., 2009)
PH	ss715597701	7	3,805,720,8	3,780,720,8	3,830,720,8	4.55E-11	3.53	Plant height 37-5	(Yao et al., 2015)
	ss715583023	2	4,733,723	4,483,723	4,983,723	4.27E-08	2.41		
	ss715611364	12	1,075,891	8,258,91	1,325,891	1.79E-07	-1.75	Plant height 38-6	(Lee et al., 2015)
	ss715622750	15	5,118,935,0	5,093,935,0	5,143,935,0	3.29E-06	1.7		
	ss715633210	19	1,596,474	1,346,474	1,846,474	3.39E-06	-1.47		
	ss715586687	3	4,642,708	4,392,708	4,892,708	5.86E-06	-1.59		
	ss715608806	11	1,099,635,9	1,074,635,9	1,124,635,9	3.49E-03	3.27	Plant height 38-5	(Lee et al., 2015)
	ss715595707	6	9,946,974	9,696,974	1,019,697,4	4.21E-05	2.88		
	ss715580365	1	5,342,517,1	5,317,517,1	5,367,517,1	2.12E-04	-1.97		
PPP	ss715583672	2	5,911,089	5,661,089	6,161,089	7.62E-09	3.44	Pod number 9-1	(Kim et al., 2012; Kuroda et al., 2013)
	ss715630698	18	4,297,242,0	4,272,242,0	4,322,242,0	4.37E-07	3.81	Pod maturity 27-5, Pod maturity 27-8, Pod maturity 29-5, Pod maturity 29-8	(Kim et al., 2012)
	ss715634803	19	3,932,988,3	3,907,988,3	3,957,988,3	3.72E-06	2.87	Pod number 1-9, Pod number 10-1, Pod number 8-1	(Zhang et al., 2010; Kuroda et al., 2013; Yang et al., 2013)
	ss715608427	10	6,477,767	6,227,767	6,727,767	6.71E-06	-2.48		
	ss715592879	6	1,228,961,8	1,203,961,8	1,253,961,8	7.36E-06	2.7	Pod maturity 26-1	(Li et al., 2008b)
	ss715599786	8	1,628,899	1,378,899	1,878,899	1.26E-05	2.48	Pod number 5-2	(Liu et al., 2011)
	ss715619696	14	5,757,301	5,507,301	6,007,301	4.29E-05	2.73	Pod dehiscence 3-2	(Kang et al., 2009)
	ss715592677	6	1,014,383,2	9,893,832	1,039,383,2	5.56E-05	-2.81		
	ss715603759	9	3,889,959,3	3,864,959,3	3,914,959,3	7.81E-05	2.11	Pod number 4-2	(Vieira et al., 2006)
	ss715594519	6	4,588,604,2	4,563,604,2	4,613,604,2	8.60E-05	-2.73	Pod number 3-3, Pod number 7-2, Pod number 3-4	(Sun et al., 2006; Palomeque et al., 2009)
	ss715623918	16	2,835,434	2,585,434	3,085,434	9.99E-05	-2.34		
	ss715587193	4	1,670,799,2	1,645,799,2	1,695,799,2	3.23E-04	-3.03	Pod number 11-4	(Li et al., 2010)
SDPP	ss715610388	11	3,278,513,0	3,253,513,0	3,303,513,0	8.44E-06	-6.42		
	ss715613299	12	6,478,153	6,228,153	6,728,153	2.04E-05	-9.16	Seed fill 5-1	(Li et al., 2008b)
	ss715578403	1	1,006,995	7,569,95	1,256,995	4.00E-05	-6.19	Seed weight 18-1.1, Seed weight 18-1.2	(Panthee et al., 2005)
	ss715637388	20	3,405,216,3	3,380,216,3	3,430,216,3	8.97E-05	-8.4		
	ss715625025	16	3,796,947	3,546,947	4,046,947	1.20E-04	5.93		
	ss715588471	4	4,733,395,8	4,708,395,8	4,758,395,8	2.21E-04	5.75	Seed number 7-2, Seed set 1-9	(Tischner et al., 2003; Li et al., 2010)
	ss715595281	6	5,107,511,2	5,082,511,2	5,132,511,2	3.11E-04	-5.6	Seed number 1-2	(Mansur et al., 1996)
SWPP	ss715617193	13	1,399,379,4	1,374,379,4	1,424,379,4	6.86E-07	-1.18	Seed weight 49-8	(Teng et al., 2009)
	ss715632502	18	5,776,665,3	5,751,665,3	5,801,665,3	1.03E-06	-0.91		
	ss715623231	15	9,333,539	9,083,539	9,583,539	1.72E-06	-1.18	Seed weight 11-2	(Lee et al., 2001)
	ss715618430	14	1,756,147,7	1,731,147,7	1,781,147,7	2.01E-05	0.81	Seed weight 36-14	(Han et al., 2012)
	ss715601564	8	3,646,948	3,396,948	3,896,948	3.40E-05	-1.53	Seed weight per plant 3-1	(Liu et al., 2011)
	ss715581293	2	1,461,874,9	1,436,874,9	1,486,874,9	3.47E-04	0.93	Seed weight 50-14, Seed yield 31-5	(Kato et al., 2014; Wang et al., 2014)
TY	ss715591954	5	3,988,682,2	3,963,682,2	4,013,682,2	6.36E-07	-10.39	Seed weight 7-3, Seed yield 20-1, Seed thickness 1-3, Seed weight 10-1, Seed weight 34-9	(Orf et al., 1999; Specht et al., 2001; Li et al., 2008a; Han et al., 2012; Jun et al., 2014)
	ss715585727	3	3,642,764,4	3,617,764,4	3,667,764,4	1.78E-05	-13.52	Seed weight 25-3, Seed weight per plant 1-4	(Chen et al., 2007)
	ss715585334	3	3,337,878,8	3,312,878,8	3,362,878,8	2.40E-05	-9.36	Seed weight per plant 1-4, Seed yield 15-13	(Kabelka et al., 2004; Chen et al., 2007)
	ss715625564	16	8,037,107	7,787,107	8,287,107	2.44E-05	15.03	Seed yield to Plant height ratio 1-3	(Mansur et al., 1996)
	ss715607541	10	4,609,347,8	4,584,347,8	4,634,347,8	4.75E-05	13.83	Seed yield 31-12	(Wang et al., 2014)
	ss715608381	10	6,160,752	5,910,752	6,410,752	6.49E-05	-8.01	Seed yield 23-15, Seed yield 32-2	(Guzman et al., 2007; Fox et al., 2015)
	ss715620131	14	9,740,27	7,240,27	1,224,027	2.80E-04	-8.89		
NN	ss715603084	9	1,548,739,3	1,523,739,3	1,573,739,3	3.00E-04	1.03		
	ss715603180	9	1,846,918,2	1,821,918,2	1,871,918,2	3.78E-04	0.96		

**Figure 9:**
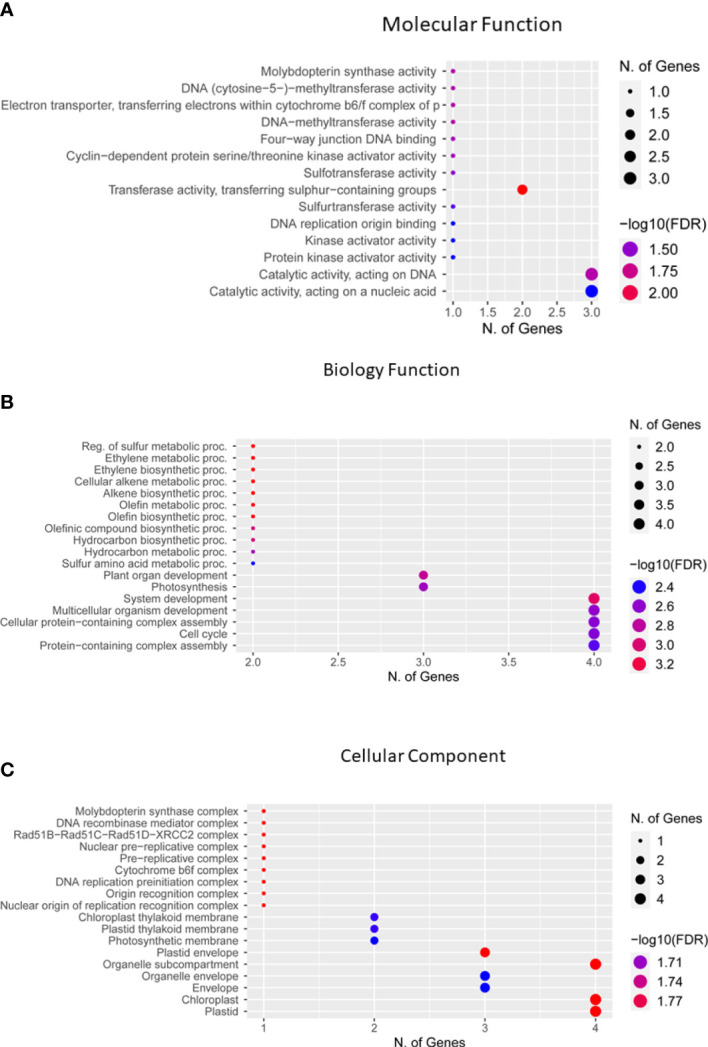
Gene ontology enrichment analysis of 17 genes for their functional categories. **(A)** Molecular function. **(B)** Biology process. **(C)** Cellular components identified using Shiny GO. Fo enrichment analysis false discovery rate (FDR) was calculated based on a p value of 0.05.

Genes located in 500-kbp genomic regions of each significant SNP were identified as candidate genes. For 51 QTNs, 2,356 genes were identified closer to significant SNPs. Gene Ontology web software ShinyGO was used to clarify the putative activities of these genes and classified them on the basis of distinct functional groups (**Figure 9**). Of these genes, 17 were found to be functionally annotated genes, while the remaining genes were hypothetical proteins with no functional annotation (**Table 3**; **Supplementary Table 4**). To confirm the function of these genes, the soybean data base SoyBase (https://www.soybase.org/) was used. Among these functionally annotated genes, *Glyma.09G171300* (GO:0017004), cytochrome b6-f complex subunit 8 is located on chromosome 9 near significant SNP ss715603826 for HGW. Candidate genes located near peak SNPs for PH were *Glyma.01G199200*, *Glyma.01G201600* (GO:0005515), *Glyma.11G143900* (GO:0033063), *Glyma.11G145300* (GO:0008146*)*, *Glyma.12G017300* (GO:0003676), and *Glyma.12G014600* (GO:0003677) encoding lsd one like 1, Tetratricopeptide repeat (TPR)-like superfamily protein, DNA repair protein RAD51 homolog 4, Protein-tyrosine sulfotransferase, binding partner of acd11 1, and Origin recognition complex subunit 3. A total of five candidate genes that were identified closer to most significant SNPs for PPP were *Glyma.02G065600* (GO:0016779), *Glyma.06G153500* (GO:0005524), *Glyma.06G153400* (GO:0016655), *Glyma.08G018500* (GO:0016787), and *Glyma.10G065700* (GO:0050613), which encode DNA polymerase lambda (POLL), ABC transporter family protein, NAD(P)H-quinone oxidoreductase subunit O, Serine/threonine-protein phosphatase, and Delta (14)-sterol reductase, respectively. For SWPP, GWAS identified seven significant SNPs; however, no annotated gene was located near four of these SNPs, while the other three SNPs have the functionally annotated genes *Glyma.01G007800* (GO:0003682), *Glyma.06G321300* (GO:0030366), and *Glyma.12G083700* (GO:0016538) on chromosomes 1, 6, and 12, respectively. These genes have the functional annotation of DNA (cytosine-5)-methyltransferase CMT3, Molybdopterin synthase sulfur carrier subunit, and Cyclin-dependent kinases regulatory subunit 2, respectively. Out of six significantly associated SNPs for SWPP, only the single SNP ss715632502 on chromosome 18 has candidate gene *Glyma.18G297900* (GO:0005488), which functions as HEAT repeat-containing protein. Similar to the SWPP, TY has only one significant SNP, ss715620131, on chromosome 14 that has the functionally annotated candidate gene *Glyma.14G009900* (GO:0061077) encoding Chaperonin-like RbcX protein. Of these 17 genes, four candidate genes, *Glyma.01g199200*, *Glyma.10g065700* (GO:0050613), *Glyma.18g297900* (GO:0005488), and *Glyma.14g009900* (GO:0061077), were located in the vicinity of the novel QTNs ss715580365, ss715608427, ss715632502, and ss715620131 (**Tables 2**, **3**), respectively.

**Table 3:** Functional annotation of potential candidate genes along with their expression tissues with respect to *Arabidopsis thaliana* homologs.

Trait	SNP	Chr	Genes	Position (bp)		*Arabidopsis* homologs	Expressed in	Functional annotation
				Start	Stop			
HGW	ss715603826	9	*Glyma.09g171300*	4,054,052,8	4,054,061,7	ATCG00210	-	Cytochrome b6-f complex subunit 8
PH	ss715580365	1	*Glyma.01g199200*	5,442,013,7	5,442,334,2	AT1G32540.2	Stem	lsd one like 1
	ss715580365	1	*Glyma.01g201600*	5,460,051,7	5,460,157,3	ATCG00360.1	Stem	Tetratricopeptide repeat (TPR)-like superfamily protein
	ss715608806	11	*Glyma.11g143900*	1,094,884,6	1,095,332,2	AT1G07745.1	NA	DNA repair protein RAD51 homolog 4
	ss715608806	11	*Glyma.11g145300*	1,737,225,7	1,737,794,1	AT1G08030.1	Stem	Protein-tyrosine sulfotransferase
	ss715611364	12	*Glyma.12g017300*	1,212,339	1,215,047	AT4G17720.1	Stem	binding partner of acd11 1
	ss715611364	12	*Glyma.12g014600*	1,040,874	1,052,808	AT5G16690.1	Stem	Origin recognition complex subunit 3
PPP	ss715583672	2	*Glyma.02g065600*	5,804,132	5,815,509	AT1G10520.1	NA	DNA polymerase lambda (POLL)
	ss715592879	6	*Glyma.06g153500*	1,248,379,6	1,248,725,0	AT4G33460.1	Seed	ABC transporter family protein
	ss715592879	6	*Glyma.06g153400*	1,248,237,1	1,248,400,8	AT1G74880.1	Seed	NAD(P)H-quinone oxidoreductase subunit O
	ss715599786	8	*Glyma.08g018500*	1,497,919	1,502,332	AT4G11240.1	Seed	Serine/threonine-protein phosphatase
	ss715608427	10	*Glyma.10g065700*	6,325,563	6,330,154	AT3G52940.1	Seed	Ergosterol biosynthesis ERG4/ERG24 family
SDPP	ss715578403	1	*Glyma.01g007800*	7,680,61	7,767,31	AT1G69770.1	Seed	DNA (cytosine-5)-methyltransferase CMT3
	ss715595281	6	*Glyma.06g321300*	5,052,678,5	5,052,967,0	AT4G10100.1	Seed	Molybdopterin synthase sulfur carrier subunit
	ss715613299	12	*Glyma.12g083700*	6,651,774	6,652,404	AT2G27970.1	Seed	Cyclin-dependent kinases regulatory subunit 2
SWPP	ss715632502	18	*Glyma.18g297900*	5,778,056,4	5,783,011,7	AT1G67140.3	Seed	HEAT repeat-containing protein
TY	ss715620131	14	*Glyma.14g009900*	7,649,36	7,674,60	AT5G19855.1	Seed	RbcX2

## 4 Discussion

Numerous studies on QTL mapping in soybean have revealed details about the genetic regions that underlie the genetic control of important agronomic traits. However, these results have very low mapping resolution. Despite being an essential source of plant protein and vegetable oil, soybean production is lower than other key crops. The precision of QTNs and the genetic diversity in the selected association panel dictate the usefulness and efficacy of MAS in a crop. More phenotypic and genotypic variation in the association panel would increase the chances of discovering QTNs and valuable alleles that might be employed as molecular markers for marker-assisted breeding (Zhao et al., 2019). Because of its significant photoperiod response, soybean was challenging to grow in unfavorable environmental conditions and grow to full maturity (Zhang et al., 2016). Breeders will always continue to focus on yield-related traits and other qualitative traits as they are directly related to the productivity and quality of crops (Bruce et al., 2019; Luo et al., 2023). When direct selection for yield is difficult, they also serve as selection goals in plant breeding programs. To promote crop development, crop germplasm collections are characterized for yield-related traits (Adeboye et al., 2021). There are reports about a complicated inheritance pattern for soybean yield and its sensitivity to the environment (Bhat et al., 2022). Therefore, improving soybean production through the manipulation of traits associated with yield has been the long-term objective of breeders. A key component of the soybean improvement method for creating varieties with greater yield potential is identifying the genetic basis of yield-related features.

Genome-wide association studies are now viewed as an important method for identifying genomic regions linked to complex traits in a variety of crops (Tibbs Cortes et al., 2021; Priyanatha et al., 2022). In the current study, GWAS was used for the identification of QTNs associated with PH, NN, PPP, SDPP, SWPP, HGW, and TY. A panel of 155 soybeans accessions and 35,100 SNPs after imputation were used for marker-trait association. LD block helps in determining the distance between the marker and candidate gene that will not undergo a crossing over event during meiosis. However, LD varies between species and populations (Li et al., 2018b). In our study, for 155 soybean accessions, the overall LD decay distance across the entire genome was 479,078 bp (r^2 = ^0.1), which was higher than the previously reported distance of 119.07 kb in cultivated soybean but within the reported range (90–574 kb) (Jiang et al., 2019). Moreover, 54,175 functionally annotated genes are present in the 975 Mb genome of cultivated soybean (Wang et al., 2016). Average SNP spacing reported in our study was 27.78 kb (**Supplementary Table 3**), with large gaps, which was theoretically enough for effective GWAS analysis; however, a high-resolution map with SNP markers can be helpful in future to find more trait-QTN relationships. In a previous study, Priyanatha et al. (2022) also reported low SNP coverage that can be improved in future GWAS studies by increasing SNP coverage with few chromosomal gaps. Improvements in GWAS can be made for lower level polymorphisms and shorter LD decay block, as proposed by He et al. (2017). In addition, some other strategies, such as mapping of LD blocks (Bandillo et al., 2015), SNPLDBs (He et al., 2017), and haplotype blocks (Greenspan and Geiger, 2004; Contreras-Soto et al., 2017), are also being used. In GWAS, panel RILs can be employed to maximize the heritability of QTNs (Viana et al., 2017; Luo et al., 2023). All of these factors may strengthen marker-trait relationships and boost the detection rate. Furthermore, Mohammadi et al. (2020) describe further techniques to improve GWAS detection of real marker-trait relationships and QTL validation.

A total of 51 QTNs were identified in this study of which 33 are colocalized with the previously reported QTLs and 18 were putatively novel QTNs (**Table 2**). Of these novel QTNs, two were identified for HGW and NN each, while 6, 4, 3, and 1 QTNs were associated with PH, SDPP, PPP, and TY respectively. After confirming the SNP validation, the information obtained from this study could be used in future breeding programs for trait introgression. These QTNs were further used to find the candidate genes in a 500 kb region.

In current study, GWAS revealed 2,356 genes for six traits based on the gene expression data and annotations. We only included 17 potential candidate genes, the activities of which were involved in controlling soybean plant height, node number, pods per plant, seeds per plant, seed weight per plant, hundred grain weight, and total yield (**Table 3**). Among these genes, *Glyma.09g171300* is proposed as a candidate gene for HGW and is located in the vicinity of ss715603826, which was previously reported by Li et al. (2018a) when identifying the role of amino acids in soybean seed. A pleiotropic cluster of six QTLs was colocated at ss715603826 on chromosome 9. This QTN showed a positive allelic effect on the HGW (**Figure 7**) and is present in a similar region with a previously reported QTL seed weight QTL viz., *Seed weight 27-3* (Vieira et al., 2006). This gene encodes Cytochrome b6-f complex subunit 8, which mediates electron transfer during photosynthesis. Yamori et al. (2016) confirmed in rice that increasing photosynthesis through the manipulation of *cyt b6f* results in an increase or decrease in plant yield.

The *Glyma.14g009900* gene that was identified in the flanking region of TY QTN is homologous to *Arabidopsis* gene *AT5G19855.1*. This gene encodes an RbcX protein that has a chaperon-like function; therefore, it plays a significant role in the correct assembly of RbcL and RbcS subunits during RuBisCO biogenesis and is also essential for the protein to attain its maximum activity (Rudi et al., 1998; Kolesiński et al., 2011). Rubisco catalyzes the first step in two opposing chemical pathways: photorespiration (using O2 as a substrate) and photosynthetic carbon fixation (using CO2 as a substrate) (Andrews and Lorimer, 1985; Erb and Zarzycki, 2018). The photosynthetic uptake of CO_2_ results in the production of functioning sugars (Gutteridge and Gatenby, 1995; Choquet and Wollman, 2023), which are responsible for plant development and yield (Saschenbrecker et al., 2007). The *Glyma.18g297900* gene that was identified in the flanking region of ss715632502, a QTN for seed weight per plant, is homologous to the *Arabidopsis* gene *AT1G67140.3* (*SWEETIE*) and encodes HEAT repeat-containing protein. In *Arabidopsis*, this gene affects carbon utilization and has major role in the growth and development stages of the plant (Veyres et al., 2008).

Three candidate genes, *Glyma.01g007800*, *Glyma.06g321300*, and *Glyma.12g083700*, were found in the flanking region of QTNs for seeds per plant. *Glyma.01g007800*, which encodes DNA (cytosine-5)-methyltransferase CMT3, is homologous to the *Arabidopsis* gene *AT1G69770.1*. DNA methylation is an epigenetic variation that regulates a variety of functions, including stress responses, expression of transposable elements (TEs), and gene expression (Gallego-Bartolomé, 2020). The methods for maintaining DNA methylation (MDM) are dependent on the context of the cytosine sequence (CG, CHG, or CHH, H=T, C, A), and they are catalyzed by several DNA methyltransferases (Zhang et al., 2018). Methyltransferase 1 (MET1) maintains CG cytosine methylation. Chromomethylase 3 (CMT3) and CMT2 sustain CHG cytosine methylation (Stroud et al., 2014). Numerous studies have shown that altering DNA methylation offers an alternate strategy for crop improvement, making it a significant target for such manipulation (King, 2015; Feng et al., 2022). In previous studies, different activations of DNA C5-MTase genes were reported during the developmental stages of embryos and seeds in *Arabidopsis*, cereals, and legumes (Sharma et al., 2009; Garg et al., 2014; Qian et al., 2014; Feng et al., 2022). Another gene, *Glyma.06g321300*, which is homologous to the *Arabidopsis* gene *AT4G10100.1*, encodes Molybdopterin synthase sulfur carrier subunit, a ubiquitin-like protein that is similar to a molybdopterin synthase small subunit called MoaD, which contains a C-terminal thiocarboxylated glycine residue that acts as a sulfur donor for molybdopterin production. In soybean, the use of Mo as a fertilizer increases total yield (Rana et al., 2020). Additionally, *Glyma.12g083700* is the gene identified for seed per plant that encodes Cyclin-dependent kinases regulatory subunit 2. The *Arabidopsis* homolog of this gene is *AT2G27970.1*, which is also known as *CKS2*. In a previous study, it was reported that *CcKS2* regulates the function of different genes by entering the nucleus and plays an important role in the developmental stages of plants (Tamirisa et al., 2017).

For pods per plant, five genes were identified in overlapping regions or near regions of four significant QTNs. Two genes, *Glyma.06g153500* and *Glyma.06g153400*, at chromosome six, overlap one another. *Glyma.06g153500* encodes ABC transporter family protein, which is homologous to the *Arabidopsis* gene *AT4G33460.1*. In *Arabidopsis*, 22 functionally analyzed ABC transporters have been identified that are involved in plant development, plant nutrition, organ growth, and responses to many biotic and abiotic stresses (Kang et al., 2011; Lü et al., 2018). Many essential cellular activities that use ATP hydrolysis to energize the transport of solutes across membranes are mediated by the ATP-binding cassette (ABC) protein family, particularly the intrinsic membrane subfamilies. The ABC transport family has been widely identified in many crops, including 130 in maize (Pang et al., 2013), 121 in rice (Moon and Jung, 2014), 179 in Brassica (Yan et al., 2017a), and 154 in tomato (Ofori et al., 2018). Previously, Mishra et al. (2019), through *in silico* analysis, identified 261 ABC genes in soybean that are present in nine different plant tissues and are involved in seven developmental stages and stress conditions. Therefore, *Glyma.06g153500* is considered as a strong candidate gene that plays an important role in soybean pods. Another candidate gene, *Glyma.06g153400*, was homologous to the *Arabidopsis* gene *AT1G74880.1*. This gene encodes NAD(P)H-quinone oxidoreductase subunit O, which is important for prenylquinone metabolism and vitamin K1 accumulation and is located in chloroplasts (Eugeni Piller et al., 2011; Vidal et al., 2018). Candidate gene *Glyma.02g065600*, which encodes DNA polymerase lambda (POLL), is homologous to the *Arabidopsis* gene *AT1G10520.1*. This gene is still novel in plants and is the only member of the X family as it is homologous to a mammalian gene. Maintenance of genome integrity is a key process in all organisms. DNA polymerases (Pols) are central players in this process as they are in charge of the faithful reproduction of the genetic information, as well as DNA repair (Pedroza-Garcia et al., 2019). The fact that the POLL promoter is activated by UV and that both overexpressing and silenced plants exhibit altered growth phenotypes support the hypothesis that DNA pol plays a significant role in plant growth (Roy, 2014). Candidate gene *Glyma.08g018500* is homologous to the *Arabidopsis* gene *AT4G11240*. This gene encodes Serine/threonine-protein phosphatase, which acts as a negative regulator of the plant defense response (País et al., 2009; Máthé et al., 2019). In soybean cotyledons, the inhibitor triggers anti-fungal defense responses even in the absence of infection or elicitors (Mackintosh et al., 1994). Another candidate gene identified for pods was *Glyma.10g065700*, which encodes Ergosterol biosynthesis ERG4/ERG24 family and is homologous to the *Arabidopsis* gene *AT3G52940.1*, which encodes sterol C-14 reductase and plays a major role in plant cell division, embryogenesis, and development (He et al., 2003).

For plant height in soybean, six genes were identified in the genomic region of three significant QTNs. Two candidate genes, *Glyma.11g143900* and *Glyma.11g145300*, were located in the CDS region of ss715608806, which has a positive additive effect of 3.27 on plant height. *Glyma.11g145300*, which encodes Protein-tyrosine sulfotransferase (TPST), is homologous to the *Arabidopsis* gene *AT1G08030.1*. TPST has been linked to a variety of significant biological processes in eukaryotic species (Zhong et al., 2020). This protein is a 500-aa type I transmembrane protein that expresses throughout the plant body. To control root development and gene expression in biological processes in *Arabidopsis*, including auxin production and accumulation, TPST is involved in fructose signaling (Zhong et al., 2020). TPST responds to the plant hormone auxin, which plays an important role in stem elongation (Zhou et al., 2010). *Glyma.11g143900* encodes the DNA repair protein RAD51 homolog 4, which is involved in the pathway of homologous recombination, which is considered as a precise DNA damage repair process (Markmann-Mulisch et al., 2007; Angelis et al., 2023). This gene was identified as homologous to the *Arabidopsis* gene *AT1G07745.1*, which plays a role in somatic homologous recombination and pathogen-related gene transcription (Durrant et al., 2007; Angelis et al., 2023). Although the precise physiological roles of the RAD51 paralogs are still not entirely understood, they operate to promote break repair and transduce the DNA damage signal to effector kinases (Bonilla et al., 2020). *Glyma.01g199200* and *Glyma.01g201600*, at chromosome 1, are proposed candidate genes for PH. QTN ss715580365 has been located in the CDS region of these two genes. *Glyma.01g199200* encodes lsd one like 1 protein. The homolog of this gene in *Arabidopsis* is *AT1G32540.2*, which is symbolized as *LOL1* and encodes plant-specific zinc finger protein and is expressed in almost all parts of plants and functions in controlled cell death (Epple et al., 2003; Borovsky et al., 2019). The rice homolog of this gene negatively regulates programmed cell death, but when it is overexpressed, it increases chlorophyll in shoots (Wang et al., 2005). In Solanaceae, this gene is involved in fruit development (Borovsky et al., 2019). Another candidate gene, *Glyma.01g201600*, which is homologous to the *Arabidopsis* gene *ATCG00360.1*, encodes Tetratricopeptide repeat (TPR)-like superfamily protein. In nature, tetratricopeptide repeat (TPR) and TPR-like domains are common. They participate in a variety of biological processes and are known for binding to short linear peptide motifs (Perez-Riba and Itzhaki, 2019). TPR proteins function in auxin, cytokinin, and gibberellin responses and ethylene production (Greenboim-Wainberg et al., 2005; Yoshida et al., 2005; Wei and Han, 2017). Auxin is an important plant hormone that promotes cell growth through stem elongation (Dilworth et al., 2017). Therefore, *Glyma.01g201600* can be considered a strong candidate gene that plays an important role in plant height. At chromosome 12, two candidate genes, *Glyma.12g017300* and *Glyma.12g014600*, were predicted for plant height. *Glyma.12g017300* encodes a binding partner of acd11 1 and is homologous to *AT4G17720.1* in *Arabidopsis*. This gene is uniformly present in land plants, which raises the possibility that this immunological regulatory module emerged in the early developmental stages of land plants and assisted in their colonization (Zhang et al., 2020). *Glyma.12g014600* encodes Origin recognition complex subunit 3, which is an important component element in plants and plays a significant role in many biological processes, including DNA replication, checkpoint regulation, heterochromatin formation, and chromosome assembly (Chen et al., 2013; Popova et al., 2018). *Glyma.12g014600* is homologous to the *Arabidopsis* gene *AT5G16690.1*, which is also known as *AtORC3*. All the members of the ORC gene family are expressed in all three stages of flowering, except *AtORC3*, which is only expressed after fertilization (Collinge et al., 2004).

The expression levels of the 17 genes described above varied significantly between extreme materials in the current investigation during the growth and developmental stages of soybean seeds. Four candidate genes, *Glyma.01g199200*, *Glyma.10g065700*, *Glyma.18g297900*, and *Glyma.14g009900*, were identified in the vicinity of the novel QTNs. Although further experimental validation of these candidate genes is required, many are involved in developmental processes controlling the expression of the respective traits, as determined through comparison with their homologs in *Arabidopsis*. Thus, we hypothesized that these 17 genes are potential candidates for PH, PPP, SDPP, SWPP, HGW, and TY. Consequently, the discovery of these fresh putative QTNs and candidate genes opens up a potential new supply of desired genetics for research and analysis. Therefore, these genes could be chosen for further investigation and potential functional confirmation to advance our understanding of how important agronomic traits in soybean are regulated.

## Conclusion

To the best of our knowledge, this study is the first to look into a genetic panel of soybean lines in Pakistan using a GWAS design to identify QTLs for soybean plant height, node number, pods per plant, seeds per plant, seed weight per plant, hundred grain weight, and total yield. This study confirmed 33 QTNs that were colocalized with previously reported QTLs for yield and its components. Additionally, 19 putative novel QTNs were identified for yield and its components using a panel of 155 diverse soybean accessions. There were 17 candidate genes within a ±250 kb region of significant SNPs. Results obtained from Gene Ontology analysis of these genes showed that most of are involved in the growth and developmental stages of soybean and hence play an important role in the final yield. By adding to the growing body of research, this work increases our understanding of the true strength of genetics underlying agronomic features in soybean. The findings of the current GWAS study, along with those from the previous reports, support the idea that exotic germplasm can serve as a source of unique genetic diversity for ongoing agricultural improvement. The current study’s limitations might be overcome in future by the addition of better SNP coverage or alternative strategies, such as high-density mapping.

## Data availability statement

The original contributions presented in the study are included in the article/**Supplementary Material**. Further inquiries can be directed to the corresponding authors.

## Author contributions

MA, GR, and RR conceived and designed the project. RR and GR conducted the experiments. RR, HA, AD, MW, and MKR analyzed the data. MR and HS provided technical inputs in executing experiments and data analysis. RR wrote the manuscript with input from HA and MR and feedback from all the authors. MR and HS proofread the manuscript. The final manuscript was read and approved by all the authors.
