# Supplementary material for: Genome-wide association study of soybean (Glycine max [L.] Merr.) germplasm for dissecting the quantitative trait nucleotides and candidate genes underlying yield-related traits
Source: Front Plant Sci. 2023 Aug 11;14:1229495. doi: 10.3389/fpls.2023.1229495 (PMC10450938; doi:10.3389/fpls.2023.1229495)
Supplement: Supplementary file 1 [file DataSheet_1.docx]

Supplementary Material

## Supplementary Table S1. Details of the germplasm investigated in this study.

| **Code** | **PI ID** | **Origin** | **Code** | **PI ID** | **Origin** |
| --- | --- | --- | --- | --- | --- |
| G1 | PI630984 | USA, Tennessee | G79 | PI547844 | USA, Georgia |
| G2 | PI595645 | USA, Georgia | G80 | PI518771 | USA, Mississippi |
| G3 | PI602597 | USA, Georgia | G81 | PI561401 | USA, Ohio |
| G4 | PI657626 | USA, Indiana | G82 | PI572237 | USA, Georgia |
| G5 | PI633610 | USA, Arkansan | G83 | PI583295 | USA, Ohio |
| G6 | PI592756 | USA, South Carolina | G84 | PI604464 | USA, Illinois |
| G7 | PI614155 | USA, Ohio | G85 | PI633735 | USA, Missouri |
| G8 | PI639740 | USA, Illinois | G86 | PI636696 | USA, Ohio |
| G9 | PI614088 | USA, Illinois | G87 | PI639282 | USA, Georgia |
| G10 | PI633609 | USA, Arkansan | G88 | PI643914 | USA, Georgia |
| G11 | PI634335 | USA, Illinois | G89 | PI644024 | USA, Georgia |
| G12 | PI598124 | USA, Missouri | G90 | PI644025 | USA, Georgia |
| G13 | PI603953 | USA, South Carolina | G91 | PI644042 | USA, Georgia |
| G14 | PI615694 | USA, South Carolina | G92 | PI644043 | USA, Georgia |
| G15 | PI647085 | USA, North Carolina | G93 | PI644044 | USA, Georgia |
| G16 | PI641156 | USA, North Carolina | G94 | PI644045 | USA, Georgia |
| G17 | PI617045 | USA, North Carolina | G95 | PI644046 | USA, Georgia |
| G18 | PI634827 | USA, Nebraska | G96 | PI644053 | USA, Georgia |
| G19 | PI614832 | USA, Nebraska | G97 | PI644054 | USA, Georgia |
| G20 | PI648270 | USA | G98 | PI644056 | USA, Georgia |
| G21 | PI633970 | USA, Arkansan | G99 | PI644057 | USA, Georgia |
| G22 | PI612157 | USA, Georgia | G100 | PI644058 | USA, Georgia |
| G23 | PI617041 | USA, South Carolina | G101 | PI644059 | USA, Georgia |
| G24 | PI598222 | USA, Tennessee | G102 | PI647082 | USA, Georgia |
| G25 | PI631122 | USA, Tennessee | G103 | PI548693 | USA, Indiana |
| G26 | PI639187 | USA, Arkansan | G104 | PI232988 | China |
| G27 | PI628836 | Brazil | G105 | PI232991 | China |
| G28 | PI628837 | Brazil | G106 | PI250844 | Iran |
| G29 | PI628838 | Brazil | G107 | PI290136 | France |
| G30 | PI628875 | Brazil | G108 | PI323555 | India |
| G31 | PI628917 | Brazil | G109 | PI323556 | India |
| G32 | PI628918 | Brazil | G110 | PI346308 | India |
| G33 | PI628928 | Brazil | G111 | PI360835 | Japan |
| G34 | PI628948 | Brazil | G112 | PI371611 | Pakistan |
| G35 | PI628953 | Brazil | G113 | PI404153 | USA, Georgia |
| G36 | PI628963 | Brazil | G114 | PI404159 | USA, Georgia |
| G37 | PI644103 | Brazil | G115 | PI404160A | USA, Georgia |
| G38 | PI548298 | USA, Ohio | G116 | PI404160B | USA, Georgia |
| G39 | PI632401 | USA, Ohio | G117 | PI404198A | China |
| G40 | PI548660 | China | G118 | PI437126A | USA, Georgia |
| G41 | PI542712 | USA, Georgia | G119 | PI437127A | USA, Georgia |
| G42 | PI612608 | USA, Delaware | G120 | PI437127B | USA, Georgia |
| G43 | PI548533 | USA, Illinois | G121 | PI437654 | China |
| G44 | PI548316 | China | G122 | PI438183 | China |
| G45 | PI548664 | USA, Florida | G123 | PI438427 | India |
| G46 | PI536009 | USA, Georgia | G124 | PI438489B | USA |
| G47 | PI553045 | USA, Georgia | G125 | PI438498 | USA |
| G48 | PI535807 | USA, Texas | G126 | PI446893 | China |
| G49 | PI614153 | USA, Ohio | G127 | PI458517 | China |
| G50 | PI508083 | USA, Minnesota | G128 | PI475822A | China |
| G51 | PI553039 | USA, Arkansas | G129 | PI475822B | China |
| G52 | PI548318 | China | G130 | PI475822C | China |
| G53 | PI548655 | USA, Mississippi | G131 | PI486355 | Korea |
| G54 | PI548573 | Canada, Ontario | G132 | PI512322C | USA, Georgia |
| G55 | PI572243 | Canada, Ontario | G133 | PI567198 | USA, Georgia |
| G56 | PI543795 | USA, Missouri | G134 | PI567199 | USA, Georgia |
| G57 | PI518664 | USA, Virginia | G135 | PI567201D | USA, Georgia |
| G58 | PI548657 | USA, Mississippi | G136 | PI567208 | USA, Georgia |
| G59 | PI548656 | China | G137 | PI567209A | USA, Georgia |
| G60 | PI548389 | USA, North Carol | G138 | PI200492 | Japan |
| G61 | PI548391 | USA, Tennessee | G139 | PI200538 | Japan |
| G62 | PI642732 | China | G140 | PI205384 | Pakistan |
| G63 | PI548400 | USA, Lowa | G141 | PI219698 | Pakistan |
| G64 | PI509044 | USA, Lowa | G142 | PI230970 | Japan |
| G65 | PI548482 | USA, Ohio | G143 | PI269518B | Pakistan |
| G66 | PI548520 | China | G144 | PI269518C | Pakistan |
| G67 | PI643146 | China | G145 | PI309658 | Pakistan |
| G68 | PI548485 | USA, Mississippi | G146 | PI346300 | India |
| G69 | PI548488 | USA, Minnesota | G147 | PI346306 | India |
| G70 | PI632402 | USA, Georgia | G148 | PI371607 | Pakistan |
| G71 | PI593654 | USA, Illinois | G149 | PI371612 | Pakistan |
| G72 | PI522236 | USA, Illinois | G150 | PI416937 | Japan |
| G73 | PI548493 | USA, Georgia | G151 | PI437126C | USA, Georgia |
| G74 | PI511813 | USA, Virginia | G152 | PI462312 | India |
| G75 | PI518671 | USA, Illinois | G153 | PI486328 | India |
| G76 | PI553042 | USA, Illinois | G154 | PI572265A | USA, Georgia |
| G77 | PI553038 | USA, Ohio | G155 | PI323278 | Pakistan |
| G78 | PI548271 | USA, North Carol |  |  |  |

**Supplementary Table S2.** Mean *Fst* values, average distance between samples within the same subpopulation, average probability value of from each individual within each cluster, and allele frequency divergence among populations.

|  |  | **Run_1** | **Run_2** | **Run_3** | **Run_4** | **Run_5** | **Run_6** | **Run_7** | **Run_8** | **Run_9** | **Run_10** | **Average** |
| --- | --- | --- | --- | --- | --- | --- | --- | --- | --- | --- | --- | --- |
| Mean Fst value | Cluster 1 | 0.0611 | 0.0607 | 0.0614 | 0.0602 | 0.0629 | 0.0631 | 0.0622 | 0.0623 | 0.5444 | 0.0627 | 0.1101 |
|  | Cluster 2 | 0.5456 | 0.545 | 0.5455 | 0.5458 | 0.5451 | 0.545 | 0.5452 | 0.5452 | 0.0628 | 0.5451 | 0.4970 |
| Average distances  (expected heterozygosity)  between individuals in same cluster | Cluster 1 | 0.3648 | 0.3649 | 0.3648 | 0.3649 | 0.3647 | 0.3647 | 0.3648 | 0.3648 | 0.2249 | 0.3647 | 0.3508 |
|  | Cluster 2 | 0.2245 | 0.2245 | 0.2245 | 0.2245 | 0.2246 | 0.2246 | 0.2245 | 0.2245 | 0.3645 | 0.2246 | 0.2385 |
| Overall proportion of membership of the sample  in each of the 2 clusters | Cluster 1 | 0.447 | 0.447 | 0.446 | 0.447 | 0.446 | 0.446 | 0.446 | 0.446 | 0.556 | 0.446 | 0.4573 |
|  | Cluster 2 | 0.553 | 0.553 | 0.554 | 0.553 | 0.554 | 0.554 | 0.554 | 0.554 | 0.444 | 0.554 | 0.5427 |
| Allele frequency divergence among populations  (Net nucleotide distance) | | 0.1259 | 0.1258 | 0.1259 | 0.1258 | 0.1261 | 0.1261 | 0.126 | 0.126 | 0.1266 | 0.126 | 0.1260 |

**Supplementary Table S3:** Summary of the SNP markers, sequence lengths, and SNP densities on the 20 chromosomes of Soybean.

| **Chromosome** | **Number of SNPs** | **Sequence length (Mb)** | **SNP density (kb/SNP)** |
| --- | --- | --- | --- |
| Chr.01 | 1400 | 56.8 | 40.57 |
| Chr.02 | 2106 | 48.51 | 23.03 |
| Chr.03 | 1406 | 45.69 | 32.5 |
| Chr.04 | 1711 | 52.31 | 30.57 |
| Chr.05 | 1556 | 42.18 | 27.11 |
| Chr.06 | 1696 | 51.28 | 30.24 |
| Chr.07 | 1834 | 44.56 | 24.3 |
| Chr.08 | 2123 | 47.01 | 22.14 |
| Chr.09 | 1659 | 50.04 | 30.16 |
| Chr.10 | 1914 | 51.49 | 26.9 |
| Chr.11 | 1371 | 34.7 | 25.31 |
| Chr.12 | 1251 | 40.05 | 32.01 |
| Chr.13 | 2239 | 45.76 | 20.44 |
| Chr.14 | 1563 | 48.95 | 31.32 |
| Chr.15 | 2097 | 51.49 | 24.55 |
| Chr.16 | 1507 | 37.81 | 25.09 |
| Chr.17 | 1678 | 41.41 | 24.68 |
| Chr.18 | 2868 | 57.96 | 20.21 |
| Chr.19 | 1835 | 50.69 | 27.62 |
| Chr.20 | 1295 | 47.86 | 36.96 |
| Mean | 1755.45 | 47.3275 | 27.7855 |

**Supplementary Table S4.** Details of the genes identified in ±250 genomic regions of significant SNPs and their gene ontology.

| Ensembl Gene ID | Entrez | Gene Type | Species | Chr | Position (Mbp) | Description |
| --- | --- | --- | --- | --- | --- | --- |
| GLYMA_06G153500 | 1.01E+08 | protein_coding | Glycine max | 6 | 12.528888 | ABC transporter I family member 10 [Source:Projected from Arabidopsis thaliana (AT4G33460) UniProtKB/Swiss-Prot;Acc:Q8H1R4] |
| GLYMA_12G017300 | NA | protein_coding | Glycine max | 12 | 1.217529 | binding partner of acd11 1 [Source:Projected from Arabidopsis thaliana (AT5G16840) TAIR;Acc:AT5G16840] |
| GLYMA_12G083700 | 1.01E+08 | protein_coding | Glycine max | 12 | 6.676322 | Cyclin-dependent kinases regulatory subunit 2 [Source:Projected from Arabidopsis thaliana (AT2G27970) UniProtKB/Swiss-Prot;Acc:Q9SJJ5] |
| GLYMA_09G171300 | NA | protein_coding | Glycine max | 9 | 39.626144 | Cytochrome b6-f complex subunit 8 [Source:Projected from Arabidopsis thaliana (ATCG00210) UniProtKB/Swiss-Prot;Acc:P61039] |
| GLYMA_10G065700 | NA | protein_coding | Glycine max | 10 | 6.325563 | Delta(14)-sterol reductase [Source:Projected from Arabidopsis thaliana (AT3G52940) UniProtKB/Swiss-Prot;Acc:Q9LDR4] |
| GLYMA_01G007800 | 1.01E+08 | protein_coding | Glycine max | 1 | 0.763291 | DNA (cytosine-5)-methyltransferase CMT3 [Source:Projected from Arabidopsis thaliana (AT1G69770) UniProtKB/Swiss-Prot;Acc:Q94F88] |
| GLYMA_02G065600 | 1.01E+08 | protein_coding | Glycine max | 2 | 5.831075 | DNA polymerase lambda [Source:Projected from Arabidopsis thaliana (AT1G10520) UniProtKB/Swiss-Prot;Acc:Q9FNY4] |
| GLYMA_11G143900 | 1.01E+08 | protein_coding | Glycine max | 11 | 10.977947 | DNA repair protein RAD51 homolog 4 [Source:Projected from Arabidopsis thaliana (AT1G07745) UniProtKB/Swiss-Prot;Acc:Q9LQQ2] |
| GLYMA_18G297900 | 1.01E+08 | protein_coding | Glycine max | 18 | 57.511194 | HEAT repeat-containing protein [Source:Projected from Arabidopsis thaliana (AT1G67140) TAIR;Acc:AT1G67140] |
| GLYMA_01G199200 | 1.01E+08 | protein_coding | Glycine max | 1 | 53.307594 | lsd one like 1 [Source:Projected from Arabidopsis thaliana (AT1G32540) TAIR;Acc:AT1G32540] |
| GLYMA_06G321300 | 1.01E+08 | protein_coding | Glycine max | 6 | 50.996993 | Molybdopterin synthase sulfur carrier subunit [Source:Projected from Arabidopsis thaliana (AT4G10100) UniProtKB/Swiss-Prot;Acc:Q9S7A3] |
| GLYMA_06G153400 | 1.01E+08 | protein_coding | Glycine max | 6 | 12.527163 | NAD(P)H-quinone oxidoreductase subunit O, chloroplastic [Source:Projected from Arabidopsis thaliana (AT1G74880) UniProtKB/Swiss-Prot;Acc:Q9S829] |
| GLYMA_12G014600 | 1.01E+08 | protein_coding | Glycine max | 12 | 1.045864 | Origin recognition complex subunit 3, Lateral root developmen [Source: Projected from Oryza sativa (Os10g0402200)] |
| GLYMA_11G145300 | 1.01E+08 | protein_coding | Glycine max | 11 | 11.167387 | Protein-tyrosine sulfotransferase [Source:Projected from Arabidopsis thaliana (AT1G08030) UniProtKB/Swiss-Prot;Acc:Q3EDG5] |
| GLYMA_14G009900 | NA | protein_coding | Glycine max | 14 | 0.764519 | RbcX2 [Source:Projected from Arabidopsis thaliana (AT5G19855) UniProtKB/TrEMBL;Acc:A0A178UCQ2] |
| GLYMA_08G018500 | 1.01E+08 | protein_coding | Glycine max | 8 | 1.498348 | Serine/threonine-protein phosphatase [Source:Projected from Arabidopsis thaliana (AT4G11240) UniProtKB/TrEMBL;Acc:A0A178V1Q1] |
| GLYMA_01G201600 | NA | protein_coding | Glycine max | 1 | 53.48787 | Tetratricopeptide repeat (TPR)-like superfamily protein [Source:Projected from Arabidopsis thaliana (ATCG00360) TAIR;Acc:ATCG00360] |
| GLYMA_01G007700 | 1.01E+08 | protein_coding | Glycine max | 1 | 0.759038 | hypothetical protein |
| GLYMA_01G007900 | 1.01E+08 | protein_coding | Glycine max | 1 | 0.774324 | hypothetical protein |
| GLYMA_01G008000 | 1.01E+08 | protein_coding | Glycine max | 1 | 0.779555 | hypothetical protein |
| GLYMA_01G008100 | 1.01E+08 | protein_coding | Glycine max | 1 | 0.787097 | hypothetical protein |
| GLYMA_01G008200 | 1.01E+08 | protein_coding | Glycine max | 1 | 0.802095 | hypothetical protein |
| GLYMA_01G008300 | 1.03E+08 | protein_coding | Glycine max | 1 | 0.813496 | hypothetical protein |
| GLYMA_01G008400 | 1.01E+08 | protein_coding | Glycine max | 1 | 0.818047 | hypothetical protein |
| GLYMA_01G008500 | 1.01E+08 | protein_coding | Glycine max | 1 | 0.827132 | hypothetical protein |
| GLYMA_01G008600 | NA | protein_coding | Glycine max | 1 | 0.837682 | hypothetical protein |
| GLYMA_01G008700 | 1.01E+08 | protein_coding | Glycine max | 1 | 0.841903 | hypothetical protein |
| GLYMA_01G008800 | NA | protein_coding | Glycine max | 1 | 0.861997 | hypothetical protein |
| GLYMA_01G008900 | 1.01E+08 | protein_coding | Glycine max | 1 | 0.865285 | hypothetical protein |
| GLYMA_01G009000 | 1.01E+08 | protein_coding | Glycine max | 1 | 0.882738 | hypothetical protein |
| GLYMA_01G009100 | NA | protein_coding | Glycine max | 1 | 0.897628 | hypothetical protein |
| GLYMA_01G009200 | 1.01E+08 | protein_coding | Glycine max | 1 | 0.901695 | hypothetical protein |
| GLYMA_01G009300 | 1.01E+08 | protein_coding | Glycine max | 1 | 0.908484 | hypothetical protein |
| GLYMA_01G009400 | 1.01E+08 | protein_coding | Glycine max | 1 | 0.920066 | hypothetical protein |
| GLYMA_01G009500 | NA | protein_coding | Glycine max | 1 | 0.934715 | hypothetical protein |
| GLYMA_01G009600 | 1.01E+08 | protein_coding | Glycine max | 1 | 0.937652 | hypothetical protein |
| GLYMA_01G009700 | 1.01E+08 | protein_coding | Glycine max | 1 | 0.952611 | hypothetical protein |
| GLYMA_01G009800 | 1.01E+08 | protein_coding | Glycine max | 1 | 0.959845 | hypothetical protein |
| GLYMA_01G009900 | 1.01E+08 | protein_coding | Glycine max | 1 | 0.967283 | hypothetical protein |
| GLYMA_01G010000 | 1.01E+08 | protein_coding | Glycine max | 1 | 0.973266 | hypothetical protein |
| GLYMA_01G010100 | 1.01E+08 | protein_coding | Glycine max | 1 | 0.980614 | hypothetical protein |
| GLYMA_01G010200 | 1.01E+08 | protein_coding | Glycine max | 1 | 0.993024 | hypothetical protein |
| GLYMA_01G010300 | 1.01E+08 | protein_coding | Glycine max | 1 | 1.00121 | hypothetical protein |
| GLYMA_01G010400 | NA | protein_coding | Glycine max | 1 | 1.0092 | hypothetical protein |
| GLYMA_01G010500 | 1E+08 | protein_coding | Glycine max | 1 | 1.018533 | hypothetical protein |
| GLYMA_01G010600 | 1.01E+08 | protein_coding | Glycine max | 1 | 1.023389 | hypothetical protein |
| GLYMA_01G010700 | 1.01E+08 | protein_coding | Glycine max | 1 | 1.029283 | hypothetical protein |
| GLYMA_01G010800 | 1.03E+08 | protein_coding | Glycine max | 1 | 1.036293 | hypothetical protein |
| GLYMA_01G010900 | 1.01E+08 | protein_coding | Glycine max | 1 | 1.047531 | hypothetical protein |
| GLYMA_01G011000 | NA | protein_coding | Glycine max | 1 | 1.053128 | hypothetical protein |
| GLYMA_01G011100 | 1.01E+08 | protein_coding | Glycine max | 1 | 1.057125 | hypothetical protein |
| GLYMA_01G011200 | 1.01E+08 | protein_coding | Glycine max | 1 | 1.066465 | hypothetical protein |
| GLYMA_01G011300 | 1.01E+08 | protein_coding | Glycine max | 1 | 1.084424 | hypothetical protein |
| GLYMA_01G011400 | 1.01E+08 | protein_coding | Glycine max | 1 | 1.087724 | hypothetical protein |
| GLYMA_01G011500 | 1.01E+08 | protein_coding | Glycine max | 1 | 1.091564 | hypothetical protein |
| GLYMA_01G011600 | 1.01E+08 | protein_coding | Glycine max | 1 | 1.122016 | hypothetical protein |
| GLYMA_01G011700 | 1.01E+08 | protein_coding | Glycine max | 1 | 1.130669 | hypothetical protein |
| GLYMA_01G011800 | NA | protein_coding | Glycine max | 1 | 1.134872 | hypothetical protein |
| GLYMA_01G011900 | 1.01E+08 | protein_coding | Glycine max | 1 | 1.140332 | hypothetical protein |
| GLYMA_01G012000 | 1.03E+08 | protein_coding | Glycine max | 1 | 1.1571 | hypothetical protein |
| GLYMA_01G012100 | 1.01E+08 | protein_coding | Glycine max | 1 | 1.16207 | hypothetical protein |
| GLYMA_01G012200 | NA | protein_coding | Glycine max | 1 | 1.181837 | hypothetical protein |
| GLYMA_01G012300 | NA | protein_coding | Glycine max | 1 | 1.193004 | hypothetical protein |
| GLYMA_01G012400 | NA | protein_coding | Glycine max | 1 | 1.19782 | hypothetical protein |
| GLYMA_01G012500 | 1.01E+08 | protein_coding | Glycine max | 1 | 1.204032 | hypothetical protein |
| GLYMA_01G012600 | NA | protein_coding | Glycine max | 1 | 1.207929 | hypothetical protein |
| GLYMA_01G012700 | 1.03E+08 | protein_coding | Glycine max | 1 | 1.21116 | hypothetical protein |
| GLYMA_01G012800 | 1.01E+08 | protein_coding | Glycine max | 1 | 1.220267 | hypothetical protein |
| GLYMA_01G012900 | NA | protein_coding | Glycine max | 1 | 1.228018 | hypothetical protein |
| GLYMA_01G013000 | NA | protein_coding | Glycine max | 1 | 1.232056 | hypothetical protein |
| GLYMA_01G013100 | 1.03E+08 | protein_coding | Glycine max | 1 | 1.233755 | hypothetical protein |
| GLYMA_01G013200 | 1.03E+08 | protein_coding | Glycine max | 1 | 1.245513 | hypothetical protein |
| GLYMA_01G197900 | 1.01E+08 | protein_coding | Glycine max | 1 | 53.190137 | hypothetical protein |
| GLYMA_01G198000 | 1.01E+08 | protein_coding | Glycine max | 1 | 53.20098 | hypothetical protein |
| GLYMA_01G198100 | 1.01E+08 | protein_coding | Glycine max | 1 | 53.209161 | hypothetical protein |
| GLYMA_01G198200 | NA | protein_coding | Glycine max | 1 | 53.21629 | hypothetical protein |
| GLYMA_01G198300 | NA | protein_coding | Glycine max | 1 | 53.218206 | hypothetical protein |
| GLYMA_01G198400 | NA | protein_coding | Glycine max | 1 | 53.219202 | hypothetical protein |
| GLYMA_01G198500 | 1.01E+08 | protein_coding | Glycine max | 1 | 53.226421 | hypothetical protein |
| GLYMA_01G198600 | NA | protein_coding | Glycine max | 1 | 53.234471 | hypothetical protein |
| GLYMA_01G198700 | 1.01E+08 | protein_coding | Glycine max | 1 | 53.251004 | hypothetical protein |
| GLYMA_01G198800 | 1.01E+08 | protein_coding | Glycine max | 1 | 53.270493 | hypothetical protein |
| GLYMA_01G198900 | 1.01E+08 | protein_coding | Glycine max | 1 | 53.282978 | hypothetical protein |
| GLYMA_01G199000 | 1.01E+08 | protein_coding | Glycine max | 1 | 53.295308 | hypothetical protein |
| GLYMA_01G199100 | 1E+08 | protein_coding | Glycine max | 1 | 53.302337 | hypothetical protein |
| GLYMA_01G199300 | NA | protein_coding | Glycine max | 1 | 53.311478 | hypothetical protein |
| GLYMA_01G199400 | 1.01E+08 | protein_coding | Glycine max | 1 | 53.321811 | hypothetical protein |
| GLYMA_01G199500 | NA | protein_coding | Glycine max | 1 | 53.326917 | hypothetical protein |
| GLYMA_01G199600 | 1.01E+08 | protein_coding | Glycine max | 1 | 53.330656 | hypothetical protein |
| GLYMA_01G199700 | 1E+08 | protein_coding | Glycine max | 1 | 53.347895 | hypothetical protein |
| GLYMA_01G199800 | NA | protein_coding | Glycine max | 1 | 53.351626 | hypothetical protein |
| GLYMA_01G199900 | NA | protein_coding | Glycine max | 1 | 53.356404 | hypothetical protein |
| GLYMA_01G200000 | 1.01E+08 | protein_coding | Glycine max | 1 | 53.360767 | hypothetical protein |
| GLYMA_01G200100 | 1.01E+08 | protein_coding | Glycine max | 1 | 53.383512 | hypothetical protein |
| GLYMA_01G200200 | 1.01E+08 | protein_coding | Glycine max | 1 | 53.391283 | hypothetical protein |
| GLYMA_01G200300 | NA | protein_coding | Glycine max | 1 | 53.399105 | hypothetical protein |
| GLYMA_01G200400 | NA | protein_coding | Glycine max | 1 | 53.401726 | hypothetical protein |
| GLYMA_01G200500 | NA | protein_coding | Glycine max | 1 | 53.404235 | hypothetical protein |
| GLYMA_01G200600 | NA | protein_coding | Glycine max | 1 | 53.423035 | hypothetical protein |
| GLYMA_01G200700 | 1.01E+08 | protein_coding | Glycine max | 1 | 53.428442 | hypothetical protein |
| GLYMA_01G200800 | 1.01E+08 | protein_coding | Glycine max | 1 | 53.438675 | hypothetical protein |
| GLYMA_01G200900 | 1.01E+08 | protein_coding | Glycine max | 1 | 53.456587 | hypothetical protein |
| GLYMA_01G201000 | NA | protein_coding | Glycine max | 1 | 53.464003 | hypothetical protein |
| GLYMA_01G201100 | 1.01E+08 | protein_coding | Glycine max | 1 | 53.472428 | hypothetical protein |
| GLYMA_01G201200 | 1.01E+08 | protein_coding | Glycine max | 1 | 53.4767 | hypothetical protein |
| GLYMA_01G201300 | NA | protein_coding | Glycine max | 1 | 53.477636 | hypothetical protein |
| GLYMA_01G201400 | NA | protein_coding | Glycine max | 1 | 53.479478 | hypothetical protein |
| GLYMA_01G201500 | NA | protein_coding | Glycine max | 1 | 53.482528 | hypothetical protein |
| GLYMA_01G201700 | NA | protein_coding | Glycine max | 1 | 53.489999 | hypothetical protein |
| GLYMA_01G201800 | NA | protein_coding | Glycine max | 1 | 53.491655 | hypothetical protein |
| GLYMA_01G201900 | NA | protein_coding | Glycine max | 1 | 53.493913 | hypothetical protein |
| GLYMA_01G202000 | 1.01E+08 | protein_coding | Glycine max | 1 | 53.495131 | hypothetical protein |
| GLYMA_01G202100 | 1.01E+08 | protein_coding | Glycine max | 1 | 53.502653 | hypothetical protein |
| GLYMA_01G202200 | NA | protein_coding | Glycine max | 1 | 53.506534 | hypothetical protein |
| GLYMA_01G202300 | NA | protein_coding | Glycine max | 1 | 53.509328 | hypothetical protein |
| GLYMA_01G202400 | NA | protein_coding | Glycine max | 1 | 53.514185 | hypothetical protein |
| GLYMA_01G202500 | 1.01E+08 | protein_coding | Glycine max | 1 | 53.518781 | hypothetical protein |
| GLYMA_01G202600 | 1.01E+08 | protein_coding | Glycine max | 1 | 53.532122 | hypothetical protein |
| GLYMA_01G202700 | 1.01E+08 | protein_coding | Glycine max | 1 | 53.546708 | hypothetical protein |
| GLYMA_01G202800 | NA | protein_coding | Glycine max | 1 | 53.555655 | hypothetical protein |
| GLYMA_01G202900 | NA | protein_coding | Glycine max | 1 | 53.556294 | hypothetical protein |
| GLYMA_01G203000 | 1.01E+08 | protein_coding | Glycine max | 1 | 53.562571 | hypothetical protein |
| GLYMA_01G203100 | 1.01E+08 | protein_coding | Glycine max | 1 | 53.583166 | hypothetical protein |
| GLYMA_01G203200 | 1.01E+08 | protein_coding | Glycine max | 1 | 53.607515 | hypothetical protein |
| GLYMA_01G203300 | 1.01E+08 | protein_coding | Glycine max | 1 | 53.631238 | hypothetical protein |
| GLYMA_01G203400 | 1.01E+08 | protein_coding | Glycine max | 1 | 53.66261 | hypothetical protein |
| GLYMA_01G203500 | 1E+08 | protein_coding | Glycine max | 1 | 53.667259 | hypothetical protein |
| GLYMA_02G049300 | 1.01E+08 | protein_coding | Glycine max | 2 | 4.486759 | hypothetical protein |
| GLYMA_02G049400 | 1.01E+08 | protein_coding | Glycine max | 2 | 4.490216 | hypothetical protein |
| GLYMA_02G049500 | NA | protein_coding | Glycine max | 2 | 4.496218 | hypothetical protein |
| GLYMA_02G049600 | NA | protein_coding | Glycine max | 2 | 4.500912 | hypothetical protein |
| GLYMA_02G049700 | 1.03E+08 | protein_coding | Glycine max | 2 | 4.511302 | hypothetical protein |
| GLYMA_02G049800 | 1.01E+08 | protein_coding | Glycine max | 2 | 4.52251 | hypothetical protein |
| GLYMA_02G049900 | NA | protein_coding | Glycine max | 2 | 4.529051 | hypothetical protein |
| GLYMA_02G050000 | NA | protein_coding | Glycine max | 2 | 4.529545 | hypothetical protein |
| GLYMA_02G050100 | 1.01E+08 | protein_coding | Glycine max | 2 | 4.538456 | hypothetical protein |
| GLYMA_02G050200 | 1.01E+08 | protein_coding | Glycine max | 2 | 4.559113 | hypothetical protein |
| GLYMA_02G050300 | 1.01E+08 | protein_coding | Glycine max | 2 | 4.565422 | hypothetical protein |
| GLYMA_02G050400 | 1.01E+08 | protein_coding | Glycine max | 2 | 4.577036 | hypothetical protein |
| GLYMA_02G050500 | NA | protein_coding | Glycine max | 2 | 4.592556 | hypothetical protein |
| GLYMA_02G050600 | 1.01E+08 | protein_coding | Glycine max | 2 | 4.596176 | hypothetical protein |
| GLYMA_02G050700 | 1.01E+08 | protein_coding | Glycine max | 2 | 4.600747 | hypothetical protein |
| GLYMA_02G050800 | NA | protein_coding | Glycine max | 2 | 4.609996 | hypothetical protein |
| GLYMA_02G050900 | NA | protein_coding | Glycine max | 2 | 4.611018 | hypothetical protein |
| GLYMA_02G051000 | NA | protein_coding | Glycine max | 2 | 4.615272 | hypothetical protein |
| GLYMA_02G051100 | 1.01E+08 | protein_coding | Glycine max | 2 | 4.622879 | hypothetical protein |
| GLYMA_02G051200 | NA | protein_coding | Glycine max | 2 | 4.646066 | hypothetical protein |
| GLYMA_02G051300 | 1.01E+08 | protein_coding | Glycine max | 2 | 4.650348 | hypothetical protein |
| GLYMA_02G051400 | 1.01E+08 | protein_coding | Glycine max | 2 | 4.655449 | hypothetical protein |
| GLYMA_02G051500 | 1.01E+08 | protein_coding | Glycine max | 2 | 4.66541 | hypothetical protein |
| GLYMA_02G051600 | 1.01E+08 | protein_coding | Glycine max | 2 | 4.676782 | hypothetical protein |
| GLYMA_02G051700 | 1.01E+08 | protein_coding | Glycine max | 2 | 4.70189 | hypothetical protein |
| GLYMA_02G051800 | NA | protein_coding | Glycine max | 2 | 4.711238 | hypothetical protein |
| GLYMA_02G051900 | 1.01E+08 | protein_coding | Glycine max | 2 | 4.73804 | hypothetical protein |
| GLYMA_02G052000 | 1.01E+08 | protein_coding | Glycine max | 2 | 4.784944 | hypothetical protein |
| GLYMA_02G052100 | NA | protein_coding | Glycine max | 2 | 4.792091 | hypothetical protein |
| GLYMA_02G052200 | 1.01E+08 | protein_coding | Glycine max | 2 | 4.794974 | hypothetical protein |
| GLYMA_02G052300 | 1.01E+08 | protein_coding | Glycine max | 2 | 4.798699 | hypothetical protein |
| GLYMA_02G052400 | 1.01E+08 | protein_coding | Glycine max | 2 | 4.80286 | hypothetical protein |
| GLYMA_02G052500 | NA | protein_coding | Glycine max | 2 | 4.814563 | hypothetical protein |
| GLYMA_02G052600 | 1.01E+08 | protein_coding | Glycine max | 2 | 4.816097 | hypothetical protein |
| GLYMA_02G052700 | 1.01E+08 | protein_coding | Glycine max | 2 | 4.820442 | hypothetical protein |
| GLYMA_02G052800 | NA | protein_coding | Glycine max | 2 | 4.828111 | hypothetical protein |
| GLYMA_02G052900 | 1.01E+08 | protein_coding | Glycine max | 2 | 4.832702 | hypothetical protein |
| GLYMA_02G053000 | 1.01E+08 | protein_coding | Glycine max | 2 | 4.842362 | hypothetical protein |
| GLYMA_02G053100 | 1.01E+08 | protein_coding | Glycine max | 2 | 4.858378 | hypothetical protein |
| GLYMA_02G053200 | 1.01E+08 | protein_coding | Glycine max | 2 | 4.863464 | hypothetical protein |
| GLYMA_02G053300 | 1E+08 | protein_coding | Glycine max | 2 | 4.873543 | hypothetical protein |
| GLYMA_02G053400 | NA | protein_coding | Glycine max | 2 | 4.879037 | hypothetical protein |
| GLYMA_02G053500 | NA | protein_coding | Glycine max | 2 | 4.883312 | hypothetical protein |
| GLYMA_02G053600 | 1.01E+08 | protein_coding | Glycine max | 2 | 4.889654 | hypothetical protein |
| GLYMA_02G053800 | 1.01E+08 | protein_coding | Glycine max | 2 | 4.89495 | hypothetical protein |
| GLYMA_02G053700 | NA | protein_coding | Glycine max | 2 | 4.895041 | hypothetical protein |
| GLYMA_02G053900 | 1.01E+08 | protein_coding | Glycine max | 2 | 4.904172 | hypothetical protein |
| GLYMA_02G054000 | NA | protein_coding | Glycine max | 2 | 4.910692 | hypothetical protein |
| GLYMA_02G054100 | NA | protein_coding | Glycine max | 2 | 4.910697 | hypothetical protein |
| GLYMA_02G054200 | NA | protein_coding | Glycine max | 2 | 4.915245 | hypothetical protein |
| GLYMA_02G054300 | NA | protein_coding | Glycine max | 2 | 4.922224 | hypothetical protein |
| GLYMA_02G054400 | 1.03E+08 | protein_coding | Glycine max | 2 | 4.934648 | hypothetical protein |
| GLYMA_02G054500 | 1.01E+08 | protein_coding | Glycine max | 2 | 4.946229 | hypothetical protein |
| GLYMA_02G054600 | NA | protein_coding | Glycine max | 2 | 4.952254 | hypothetical protein |
| GLYMA_02G054700 | NA | protein_coding | Glycine max | 2 | 4.955285 | hypothetical protein |
| GLYMA_02G054800 | NA | protein_coding | Glycine max | 2 | 4.956488 | hypothetical protein |
| GLYMA_02G054900 | 1.01E+08 | protein_coding | Glycine max | 2 | 4.969683 | hypothetical protein |
| GLYMA_02G055000 | NA | protein_coding | Glycine max | 2 | 4.974147 | hypothetical protein |
| GLYMA_02G055100 | 1.01E+08 | protein_coding | Glycine max | 2 | 4.977623 | hypothetical protein |
| GLYMA_02G062800 | 1.01E+08 | protein_coding | Glycine max | 2 | 5.663668 | hypothetical protein |
| GLYMA_02G062900 | 1.01E+08 | protein_coding | Glycine max | 2 | 5.671018 | hypothetical protein |
| GLYMA_02G063000 | NA | protein_coding | Glycine max | 2 | 5.674394 | hypothetical protein |
| GLYMA_02G063100 | NA | protein_coding | Glycine max | 2 | 5.675594 | hypothetical protein |
| GLYMA_02G063200 | NA | protein_coding | Glycine max | 2 | 5.680261 | hypothetical protein |
| GLYMA_02G063300 | 1.07E+08 | protein_coding | Glycine max | 2 | 5.685209 | hypothetical protein |
| GLYMA_02G063400 | 1.01E+08 | protein_coding | Glycine max | 2 | 5.688033 | hypothetical protein |
| GLYMA_02G063500 | 1.01E+08 | protein_coding | Glycine max | 2 | 5.69952 | hypothetical protein |
| GLYMA_02G063600 | 1.01E+08 | protein_coding | Glycine max | 2 | 5.705744 | hypothetical protein |
| GLYMA_02G063700 | 1.01E+08 | protein_coding | Glycine max | 2 | 5.71696 | hypothetical protein |
| GLYMA_02G063800 | NA | protein_coding | Glycine max | 2 | 5.72059 | hypothetical protein |
| GLYMA_02G063900 | 1.01E+08 | protein_coding | Glycine max | 2 | 5.726822 | hypothetical protein |
| GLYMA_02G064000 | NA | protein_coding | Glycine max | 2 | 5.73338 | hypothetical protein |
| GLYMA_02G064100 | 1.01E+08 | protein_coding | Glycine max | 2 | 5.741318 | hypothetical protein |
| GLYMA_02G064200 | 1.01E+08 | protein_coding | Glycine max | 2 | 5.745915 | hypothetical protein |
| GLYMA_02G064300 | 1.01E+08 | protein_coding | Glycine max | 2 | 5.757818 | hypothetical protein |
| GLYMA_02G064400 | NA | protein_coding | Glycine max | 2 | 5.766138 | hypothetical protein |
| GLYMA_02G064500 | 1.01E+08 | protein_coding | Glycine max | 2 | 5.771125 | hypothetical protein |
| GLYMA_02G064600 | 1.01E+08 | protein_coding | Glycine max | 2 | 5.777446 | hypothetical protein |
| GLYMA_02G064700 | NA | protein_coding | Glycine max | 2 | 5.781636 | hypothetical protein |
| GLYMA_02G064800 | 1.01E+08 | protein_coding | Glycine max | 2 | 5.785451 | hypothetical protein |
| GLYMA_02G064900 | 1.01E+08 | protein_coding | Glycine max | 2 | 5.793297 | hypothetical protein |
| GLYMA_02G065000 | NA | protein_coding | Glycine max | 2 | 5.797404 | hypothetical protein |
| GLYMA_02G065100 | 1.01E+08 | protein_coding | Glycine max | 2 | 5.803054 | hypothetical protein |
| GLYMA_02G065200 | 1.01E+08 | protein_coding | Glycine max | 2 | 5.807044 | hypothetical protein |
| GLYMA_02G065300 | 1.01E+08 | protein_coding | Glycine max | 2 | 5.811685 | hypothetical protein |
| GLYMA_02G065400 | 1.01E+08 | protein_coding | Glycine max | 2 | 5.818124 | hypothetical protein |
| GLYMA_02G065500 | 1.01E+08 | protein_coding | Glycine max | 2 | 5.825365 | hypothetical protein |
| GLYMA_02G065700 | NA | protein_coding | Glycine max | 2 | 5.844253 | hypothetical protein |
| GLYMA_02G065800 | NA | protein_coding | Glycine max | 2 | 5.845126 | hypothetical protein |
| GLYMA_02G065900 | NA | protein_coding | Glycine max | 2 | 5.849455 | hypothetical protein |
| GLYMA_02G066000 | 1.01E+08 | protein_coding | Glycine max | 2 | 5.851639 | hypothetical protein |
| GLYMA_02G066100 | NA | protein_coding | Glycine max | 2 | 5.865034 | hypothetical protein |
| GLYMA_02G066200 | 1.01E+08 | protein_coding | Glycine max | 2 | 5.878333 | hypothetical protein |
| GLYMA_02G066300 | NA | protein_coding | Glycine max | 2 | 5.893358 | hypothetical protein |
| GLYMA_02G066400 | 1.01E+08 | protein_coding | Glycine max | 2 | 5.897892 | hypothetical protein |
| GLYMA_02G066500 | 1.01E+08 | protein_coding | Glycine max | 2 | 5.903451 | hypothetical protein |
| GLYMA_02G066600 | 1.01E+08 | protein_coding | Glycine max | 2 | 5.906703 | hypothetical protein |
| GLYMA_02G066700 | 1.01E+08 | protein_coding | Glycine max | 2 | 5.914478 | hypothetical protein |
| GLYMA_02G066800 | 1.01E+08 | protein_coding | Glycine max | 2 | 5.926127 | hypothetical protein |
| GLYMA_02G066900 | NA | protein_coding | Glycine max | 2 | 5.934162 | hypothetical protein |
| GLYMA_02G067000 | 1.01E+08 | protein_coding | Glycine max | 2 | 5.939206 | hypothetical protein |
| GLYMA_02G067100 | 1.01E+08 | protein_coding | Glycine max | 2 | 5.946276 | hypothetical protein |
| GLYMA_02G067200 | NA | protein_coding | Glycine max | 2 | 5.951938 | hypothetical protein |
| GLYMA_02G067300 | 1E+08 | protein_coding | Glycine max | 2 | 5.954618 | hypothetical protein |
| GLYMA_02G067400 | 1E+08 | protein_coding | Glycine max | 2 | 5.957522 | hypothetical protein |
| GLYMA_02G067500 | 1.01E+08 | protein_coding | Glycine max | 2 | 5.96364 | hypothetical protein |
| GLYMA_02G067600 | NA | protein_coding | Glycine max | 2 | 5.975111 | hypothetical protein |
| GLYMA_02G067700 | NA | protein_coding | Glycine max | 2 | 5.977558 | hypothetical protein |
| GLYMA_02G067800 | NA | protein_coding | Glycine max | 2 | 5.982812 | hypothetical protein |
| GLYMA_02G067900 | NA | protein_coding | Glycine max | 2 | 5.986285 | hypothetical protein |
| GLYMA_02G068000 | 1.01E+08 | protein_coding | Glycine max | 2 | 5.991168 | hypothetical protein |
| GLYMA_02G068100 | 1.01E+08 | protein_coding | Glycine max | 2 | 6.001135 | hypothetical protein |
| GLYMA_02G068200 | 1.01E+08 | protein_coding | Glycine max | 2 | 6.008538 | hypothetical protein |
| GLYMA_02G068300 | 1.01E+08 | protein_coding | Glycine max | 2 | 6.01384 | hypothetical protein |
| GLYMA_02G068400 | 1.01E+08 | protein_coding | Glycine max | 2 | 6.019578 | hypothetical protein |
| GLYMA_02G068500 | 1.01E+08 | protein_coding | Glycine max | 2 | 6.025965 | hypothetical protein |
| GLYMA_02G068600 | 1.01E+08 | protein_coding | Glycine max | 2 | 6.031332 | hypothetical protein |
| GLYMA_02G068700 | NA | protein_coding | Glycine max | 2 | 6.038862 | hypothetical protein |
| GLYMA_02G068800 | NA | protein_coding | Glycine max | 2 | 6.042913 | hypothetical protein |
| GLYMA_02G068900 | NA | protein_coding | Glycine max | 2 | 6.051752 | hypothetical protein |
| GLYMA_02G069000 | 1.01E+08 | protein_coding | Glycine max | 2 | 6.060576 | hypothetical protein |
| GLYMA_02G069100 | 1E+08 | protein_coding | Glycine max | 2 | 6.072361 | hypothetical protein |
| GLYMA_02G069200 | NA | protein_coding | Glycine max | 2 | 6.099269 | hypothetical protein |
| GLYMA_02G069300 | NA | protein_coding | Glycine max | 2 | 6.104135 | hypothetical protein |
| GLYMA_02G069400 | NA | protein_coding | Glycine max | 2 | 6.108481 | hypothetical protein |
| GLYMA_02G069500 | NA | protein_coding | Glycine max | 2 | 6.116379 | hypothetical protein |
| GLYMA_02G069600 | NA | protein_coding | Glycine max | 2 | 6.125872 | hypothetical protein |
| GLYMA_02G069700 | 1.01E+08 | protein_coding | Glycine max | 2 | 6.131843 | hypothetical protein |
| GLYMA_02G069800 | 1.01E+08 | protein_coding | Glycine max | 2 | 6.136124 | hypothetical protein |
| GLYMA_02G069900 | 1.01E+08 | protein_coding | Glycine max | 2 | 6.143958 | hypothetical protein |
| GLYMA_02G070000 | 1.01E+08 | protein_coding | Glycine max | 2 | 6.157247 | hypothetical protein |
| GLYMA_02G136800 | 1.01E+08 | protein_coding | Glycine max | 2 | 14.138973 | hypothetical protein |
| GLYMA_02G136900 | NA | protein_coding | Glycine max | 2 | 14.15837 | hypothetical protein |
| GLYMA_02G137000 | 1.01E+08 | protein_coding | Glycine max | 2 | 14.164694 | hypothetical protein |
| GLYMA_02G137100 | NA | protein_coding | Glycine max | 2 | 14.181768 | hypothetical protein |
| GLYMA_02G137200 | 1.01E+08 | protein_coding | Glycine max | 2 | 14.188791 | hypothetical protein |
| GLYMA_02G137300 | 1.01E+08 | protein_coding | Glycine max | 2 | 14.197509 | hypothetical protein |
| GLYMA_02G137400 | 1.01E+08 | protein_coding | Glycine max | 2 | 14.214097 | hypothetical protein |
| GLYMA_02G137500 | 1.01E+08 | protein_coding | Glycine max | 2 | 14.220675 | hypothetical protein |
| GLYMA_02G137600 | 1.01E+08 | protein_coding | Glycine max | 2 | 14.228005 | hypothetical protein |
| GLYMA_02G137700 | 1.01E+08 | protein_coding | Glycine max | 2 | 14.232594 | hypothetical protein |
| GLYMA_02G137800 | 1.01E+08 | protein_coding | Glycine max | 2 | 14.259785 | hypothetical protein |
| GLYMA_02G137900 | 1.01E+08 | protein_coding | Glycine max | 2 | 14.270263 | hypothetical protein |
| GLYMA_02G138000 | 1.01E+08 | protein_coding | Glycine max | 2 | 14.281246 | hypothetical protein |
| GLYMA_02G138100 | 547808 | protein_coding | Glycine max | 2 | 14.302427 | hypothetical protein |
| GLYMA_02G138200 | NA | protein_coding | Glycine max | 2 | 14.307786 | hypothetical protein |
| GLYMA_02G138300 | 1.01E+08 | protein_coding | Glycine max | 2 | 14.314317 | hypothetical protein |
| GLYMA_02G138400 | 1.01E+08 | protein_coding | Glycine max | 2 | 14.334884 | hypothetical protein |
| GLYMA_02G138500 | 1.01E+08 | protein_coding | Glycine max | 2 | 14.346881 | hypothetical protein |
| GLYMA_02G138600 | 1.01E+08 | protein_coding | Glycine max | 2 | 14.362037 | hypothetical protein |
| GLYMA_02G138700 | 1.01E+08 | protein_coding | Glycine max | 2 | 14.365344 | hypothetical protein |
| GLYMA_02G138800 | 547616 | protein_coding | Glycine max | 2 | 14.37979 | hypothetical protein |
| GLYMA_02G138900 | NA | protein_coding | Glycine max | 2 | 14.385556 | hypothetical protein |
| GLYMA_02G139000 | NA | protein_coding | Glycine max | 2 | 14.39106 | hypothetical protein |
| GLYMA_02G139100 | NA | protein_coding | Glycine max | 2 | 14.396242 | hypothetical protein |
| GLYMA_02G139200 | 1.03E+08 | protein_coding | Glycine max | 2 | 14.4009 | hypothetical protein |
| GLYMA_02G139300 | NA | protein_coding | Glycine max | 2 | 14.413609 | hypothetical protein |
| GLYMA_02G139400 | NA | protein_coding | Glycine max | 2 | 14.421969 | hypothetical protein |
| GLYMA_02G139500 | 1.01E+08 | protein_coding | Glycine max | 2 | 14.477175 | hypothetical protein |
| GLYMA_02G139600 | 1.01E+08 | protein_coding | Glycine max | 2 | 14.481572 | hypothetical protein |
| GLYMA_02G139700 | NA | protein_coding | Glycine max | 2 | 14.495636 | hypothetical protein |
| GLYMA_02G139800 | 1.01E+08 | protein_coding | Glycine max | 2 | 14.501287 | hypothetical protein |
| GLYMA_02G139900 | 1.01E+08 | protein_coding | Glycine max | 2 | 14.504848 | hypothetical protein |
| GLYMA_02G140000 | NA | protein_coding | Glycine max | 2 | 14.520895 | hypothetical protein |
| GLYMA_02G140100 | 1.01E+08 | protein_coding | Glycine max | 2 | 14.52785 | hypothetical protein |
| GLYMA_02G140200 | 1.01E+08 | protein_coding | Glycine max | 2 | 14.53242 | hypothetical protein |
| GLYMA_02G140300 | NA | protein_coding | Glycine max | 2 | 14.544281 | hypothetical protein |
| GLYMA_02G140400 | NA | protein_coding | Glycine max | 2 | 14.544839 | hypothetical protein |
| GLYMA_02G140500 | 1.01E+08 | protein_coding | Glycine max | 2 | 14.552503 | hypothetical protein |
| GLYMA_02G140600 | 1.01E+08 | protein_coding | Glycine max | 2 | 14.556126 | hypothetical protein |
| GLYMA_02G140700 | NA | protein_coding | Glycine max | 2 | 14.56243 | hypothetical protein |
| GLYMA_02G140800 | 1.07E+08 | protein_coding | Glycine max | 2 | 14.573335 | hypothetical protein |
| GLYMA_02G140900 | NA | protein_coding | Glycine max | 2 | 14.575863 | hypothetical protein |
| GLYMA_02G141000 | NA | protein_coding | Glycine max | 2 | 14.586815 | hypothetical protein |
| GLYMA_02G141100 | NA | protein_coding | Glycine max | 2 | 14.602345 | hypothetical protein |
| GLYMA_02G141200 | NA | protein_coding | Glycine max | 2 | 14.606922 | hypothetical protein |
| GLYMA_02G141300 | NA | protein_coding | Glycine max | 2 | 14.610331 | hypothetical protein |
| GLYMA_02G141400 | 1.01E+08 | protein_coding | Glycine max | 2 | 14.620729 | hypothetical protein |
| GLYMA_02G141500 | 1.01E+08 | protein_coding | Glycine max | 2 | 14.624985 | hypothetical protein |
| GLYMA_02G141600 | 1.01E+08 | protein_coding | Glycine max | 2 | 14.635999 | hypothetical protein |
| GLYMA_02G141700 | NA | protein_coding | Glycine max | 2 | 14.642349 | hypothetical protein |
| GLYMA_02G141800 | NA | protein_coding | Glycine max | 2 | 14.645359 | hypothetical protein |
| GLYMA_02G141900 | 1.01E+08 | protein_coding | Glycine max | 2 | 14.653576 | hypothetical protein |
| GLYMA_02G142000 | NA | protein_coding | Glycine max | 2 | 14.666976 | hypothetical protein |
| GLYMA_02G142100 | 1.01E+08 | protein_coding | Glycine max | 2 | 14.668246 | hypothetical protein |
| GLYMA_02G142200 | NA | protein_coding | Glycine max | 2 | 14.674021 | hypothetical protein |
| GLYMA_02G142300 | 1.01E+08 | protein_coding | Glycine max | 2 | 14.678762 | hypothetical protein |
| GLYMA_02G142400 | NA | protein_coding | Glycine max | 2 | 14.688639 | hypothetical protein |
| GLYMA_02G142500 | NA | protein_coding | Glycine max | 2 | 14.690185 | hypothetical protein |
| GLYMA_02G142600 | NA | protein_coding | Glycine max | 2 | 14.709703 | hypothetical protein |
| GLYMA_02G142700 | 1.01E+08 | protein_coding | Glycine max | 2 | 14.716292 | hypothetical protein |
| GLYMA_02G142800 | NA | protein_coding | Glycine max | 2 | 14.721446 | hypothetical protein |
| GLYMA_02G142900 | NA | protein_coding | Glycine max | 2 | 14.730118 | hypothetical protein |
| GLYMA_02G143000 | 1.01E+08 | protein_coding | Glycine max | 2 | 14.735028 | hypothetical protein |
| GLYMA_02G143100 | 1.01E+08 | protein_coding | Glycine max | 2 | 14.757825 | hypothetical protein |
| GLYMA_02G143200 | 1E+08 | protein_coding | Glycine max | 2 | 14.770876 | hypothetical protein |
| GLYMA_02G143300 | 1.01E+08 | protein_coding | Glycine max | 2 | 14.781618 | hypothetical protein |
| GLYMA_02G143400 | 1.01E+08 | protein_coding | Glycine max | 2 | 14.792918 | hypothetical protein |
| GLYMA_02G143500 | NA | protein_coding | Glycine max | 2 | 14.814133 | hypothetical protein |
| GLYMA_02G143600 | NA | protein_coding | Glycine max | 2 | 14.821658 | hypothetical protein |
| GLYMA_02G143700 | 1.01E+08 | protein_coding | Glycine max | 2 | 14.826269 | hypothetical protein |
| GLYMA_02G143800 | NA | protein_coding | Glycine max | 2 | 14.832657 | hypothetical protein |
| GLYMA_02G143900 | NA | protein_coding | Glycine max | 2 | 14.837074 | hypothetical protein |
| GLYMA_02G144000 | NA | protein_coding | Glycine max | 2 | 14.842324 | hypothetical protein |
| GLYMA_02G144100 | NA | protein_coding | Glycine max | 2 | 14.847438 | hypothetical protein |
| GLYMA_02G144200 | 1.01E+08 | protein_coding | Glycine max | 2 | 14.852054 | hypothetical protein |
| GLYMA_03G036400 | NA | protein_coding | Glycine max | 3 | 4.402337 | hypothetical protein |
| GLYMA_03G036500 | 1E+08 | protein_coding | Glycine max | 3 | 4.402746 | hypothetical protein |
| GLYMA_03G036600 | 1.01E+08 | protein_coding | Glycine max | 3 | 4.43291 | hypothetical protein |
| GLYMA_03G036700 | NA | protein_coding | Glycine max | 3 | 4.440551 | hypothetical protein |
| GLYMA_03G036800 | 547805 | protein_coding | Glycine max | 3 | 4.472672 | hypothetical protein |
| GLYMA_03G036900 | NA | protein_coding | Glycine max | 3 | 4.497651 | hypothetical protein |
| GLYMA_03G037000 | 1.01E+08 | protein_coding | Glycine max | 3 | 4.515956 | hypothetical protein |
| GLYMA_03G037100 | 1.01E+08 | protein_coding | Glycine max | 3 | 4.541759 | hypothetical protein |
| GLYMA_03G037200 | NA | protein_coding | Glycine max | 3 | 4.558538 | hypothetical protein |
| GLYMA_03G037300 | 1.01E+08 | protein_coding | Glycine max | 3 | 4.566741 | hypothetical protein |
| GLYMA_03G037400 | NA | protein_coding | Glycine max | 3 | 4.593854 | hypothetical protein |
| GLYMA_03G037500 | 1.01E+08 | protein_coding | Glycine max | 3 | 4.607433 | hypothetical protein |
| GLYMA_03G037600 | 1.01E+08 | protein_coding | Glycine max | 3 | 4.638539 | hypothetical protein |
| GLYMA_03G037700 | NA | protein_coding | Glycine max | 3 | 4.641633 | hypothetical protein |
| GLYMA_03G037800 | 1.01E+08 | protein_coding | Glycine max | 3 | 4.656313 | hypothetical protein |
| GLYMA_03G037900 | 1.01E+08 | protein_coding | Glycine max | 3 | 4.661075 | hypothetical protein |
| GLYMA_03G038000 | NA | protein_coding | Glycine max | 3 | 4.686063 | hypothetical protein |
| GLYMA_03G038100 | 1.01E+08 | protein_coding | Glycine max | 3 | 4.704295 | hypothetical protein |
| GLYMA_03G038200 | NA | protein_coding | Glycine max | 3 | 4.714568 | hypothetical protein |
| GLYMA_03G038300 | NA | protein_coding | Glycine max | 3 | 4.726589 | hypothetical protein |
| GLYMA_03G038400 | NA | protein_coding | Glycine max | 3 | 4.742089 | hypothetical protein |
| GLYMA_03G038500 | 1.01E+08 | protein_coding | Glycine max | 3 | 4.751229 | hypothetical protein |
| GLYMA_03G038600 | 1.01E+08 | protein_coding | Glycine max | 3 | 4.770005 | hypothetical protein |
| GLYMA_03G038700 | 1.01E+08 | protein_coding | Glycine max | 3 | 4.780419 | hypothetical protein |
| GLYMA_03G038800 | 1.01E+08 | protein_coding | Glycine max | 3 | 4.790555 | hypothetical protein |
| GLYMA_03G038900 | NA | protein_coding | Glycine max | 3 | 4.80718 | hypothetical protein |
| GLYMA_03G039000 | NA | protein_coding | Glycine max | 3 | 4.816969 | hypothetical protein |
| GLYMA_03G039100 | NA | protein_coding | Glycine max | 3 | 4.828493 | hypothetical protein |
| GLYMA_03G039200 | NA | protein_coding | Glycine max | 3 | 4.852112 | hypothetical protein |
| GLYMA_03G039300 | 1.01E+08 | protein_coding | Glycine max | 3 | 4.890564 | hypothetical protein |
| GLYMA_03G120000 | 1.01E+08 | protein_coding | Glycine max | 3 | 33.134131 | hypothetical protein |
| GLYMA_03G120100 | 1.01E+08 | protein_coding | Glycine max | 3 | 33.174618 | hypothetical protein |
| GLYMA_03G120200 | 1.01E+08 | protein_coding | Glycine max | 3 | 33.183157 | hypothetical protein |
| GLYMA_03G120300 | NA | protein_coding | Glycine max | 3 | 33.189255 | hypothetical protein |
| GLYMA_03G120400 | 1.01E+08 | protein_coding | Glycine max | 3 | 33.190922 | hypothetical protein |
| GLYMA_03G120500 | NA | protein_coding | Glycine max | 3 | 33.198299 | hypothetical protein |
| GLYMA_03G120600 | NA | protein_coding | Glycine max | 3 | 33.212882 | hypothetical protein |
| GLYMA_03G120700 | 1.01E+08 | protein_coding | Glycine max | 3 | 33.242128 | hypothetical protein |
| GLYMA_03G120800 | NA | protein_coding | Glycine max | 3 | 33.26551 | hypothetical protein |
| GLYMA_03G120900 | NA | protein_coding | Glycine max | 3 | 33.269899 | hypothetical protein |
| GLYMA_03G121000 | 1.01E+08 | protein_coding | Glycine max | 3 | 33.279455 | hypothetical protein |
| GLYMA_03G121100 | 1.01E+08 | protein_coding | Glycine max | 3 | 33.299143 | hypothetical protein |
| GLYMA_03G121200 | 1.01E+08 | protein_coding | Glycine max | 3 | 33.312493 | hypothetical protein |
| GLYMA_03G121300 | 1.01E+08 | protein_coding | Glycine max | 3 | 33.331068 | hypothetical protein |
| GLYMA_03G121400 | NA | protein_coding | Glycine max | 3 | 33.37837 | hypothetical protein |
| GLYMA_03G121500 | NA | protein_coding | Glycine max | 3 | 33.386327 | hypothetical protein |
| GLYMA_03G121600 | NA | protein_coding | Glycine max | 3 | 33.418557 | hypothetical protein |
| GLYMA_03G121700 | NA | protein_coding | Glycine max | 3 | 33.428209 | hypothetical protein |
| GLYMA_03G121800 | NA | protein_coding | Glycine max | 3 | 33.438969 | hypothetical protein |
| GLYMA_03G121900 | NA | protein_coding | Glycine max | 3 | 33.446807 | hypothetical protein |
| GLYMA_03G122000 | 1.01E+08 | protein_coding | Glycine max | 3 | 33.466244 | hypothetical protein |
| GLYMA_03G122100 | 1.01E+08 | protein_coding | Glycine max | 3 | 33.475612 | hypothetical protein |
| GLYMA_03G122200 | NA | protein_coding | Glycine max | 3 | 33.489549 | hypothetical protein |
| GLYMA_03G122300 | 1.01E+08 | protein_coding | Glycine max | 3 | 33.525303 | hypothetical protein |
| GLYMA_03G122400 | 1.01E+08 | protein_coding | Glycine max | 3 | 33.531218 | hypothetical protein |
| GLYMA_03G122500 | 1.01E+08 | protein_coding | Glycine max | 3 | 33.543921 | hypothetical protein |
| GLYMA_03G122600 | NA | protein_coding | Glycine max | 3 | 33.546417 | hypothetical protein |
| GLYMA_03G122700 | NA | protein_coding | Glycine max | 3 | 33.560986 | hypothetical protein |
| GLYMA_03G122800 | NA | protein_coding | Glycine max | 3 | 33.563422 | hypothetical protein |
| GLYMA_03G122900 | 1.01E+08 | protein_coding | Glycine max | 3 | 33.565981 | hypothetical protein |
| GLYMA_03G123000 | NA | protein_coding | Glycine max | 3 | 33.577235 | hypothetical protein |
| GLYMA_03G123100 | NA | protein_coding | Glycine max | 3 | 33.595167 | hypothetical protein |
| GLYMA_03G123200 | NA | protein_coding | Glycine max | 3 | 33.601074 | hypothetical protein |
| GLYMA_03G123300 | NA | protein_coding | Glycine max | 3 | 33.626007 | hypothetical protein |
| GLYMA_03G146500 | 1.01E+08 | protein_coding | Glycine max | 3 | 36.181312 | hypothetical protein |
| GLYMA_03G146600 | NA | protein_coding | Glycine max | 3 | 36.191841 | hypothetical protein |
| GLYMA_03G146700 | 1.01E+08 | protein_coding | Glycine max | 3 | 36.196552 | hypothetical protein |
| GLYMA_03G146800 | NA | protein_coding | Glycine max | 3 | 36.222301 | hypothetical protein |
| GLYMA_03G146900 | 1.01E+08 | protein_coding | Glycine max | 3 | 36.240797 | hypothetical protein |
| GLYMA_03G147000 | 1.01E+08 | protein_coding | Glycine max | 3 | 36.260002 | hypothetical protein |
| GLYMA_03G147100 | NA | protein_coding | Glycine max | 3 | 36.270701 | hypothetical protein |
| GLYMA_03G147200 | 1.01E+08 | protein_coding | Glycine max | 3 | 36.286398 | hypothetical protein |
| GLYMA_03G147300 | NA | protein_coding | Glycine max | 3 | 36.293061 | hypothetical protein |
| GLYMA_03G147400 | NA | protein_coding | Glycine max | 3 | 36.293698 | hypothetical protein |
| GLYMA_03G147500 | NA | protein_coding | Glycine max | 3 | 36.29523 | hypothetical protein |
| GLYMA_03G147600 | 1.01E+08 | protein_coding | Glycine max | 3 | 36.298168 | hypothetical protein |
| GLYMA_03G147700 | NA | protein_coding | Glycine max | 3 | 36.32906 | hypothetical protein |
| GLYMA_03G147800 | 1.01E+08 | protein_coding | Glycine max | 3 | 36.338586 | hypothetical protein |
| GLYMA_03G147900 | NA | protein_coding | Glycine max | 3 | 36.353586 | hypothetical protein |
| GLYMA_03G148000 | 1.01E+08 | protein_coding | Glycine max | 3 | 36.364813 | hypothetical protein |
| GLYMA_03G148100 | 1.01E+08 | protein_coding | Glycine max | 3 | 36.368592 | hypothetical protein |
| GLYMA_03G148200 | 1E+08 | protein_coding | Glycine max | 3 | 36.371047 | hypothetical protein |
| GLYMA_03G148300 | 1.01E+08 | protein_coding | Glycine max | 3 | 36.396979 | hypothetical protein |
| GLYMA_03G148400 | 1.01E+08 | protein_coding | Glycine max | 3 | 36.420379 | hypothetical protein |
| GLYMA_03G148500 | 1.01E+08 | protein_coding | Glycine max | 3 | 36.426486 | hypothetical protein |
| GLYMA_03G148600 | NA | protein_coding | Glycine max | 3 | 36.431048 | hypothetical protein |
| GLYMA_03G148700 | 1.01E+08 | protein_coding | Glycine max | 3 | 36.433817 | hypothetical protein |
| GLYMA_03G148800 | 1.01E+08 | protein_coding | Glycine max | 3 | 36.442696 | hypothetical protein |
| GLYMA_03G148900 | NA | protein_coding | Glycine max | 3 | 36.449311 | hypothetical protein |
| GLYMA_03G149000 | 1.01E+08 | protein_coding | Glycine max | 3 | 36.454104 | hypothetical protein |
| GLYMA_03G149100 | NA | protein_coding | Glycine max | 3 | 36.461605 | hypothetical protein |
| GLYMA_03G149200 | 1.01E+08 | protein_coding | Glycine max | 3 | 36.464868 | hypothetical protein |
| GLYMA_03G149300 | NA | protein_coding | Glycine max | 3 | 36.473156 | hypothetical protein |
| GLYMA_03G149400 | 1.01E+08 | protein_coding | Glycine max | 3 | 36.477239 | hypothetical protein |
| GLYMA_03G149500 | 1.01E+08 | protein_coding | Glycine max | 3 | 36.48329 | hypothetical protein |
| GLYMA_03G149600 | 1.01E+08 | protein_coding | Glycine max | 3 | 36.491781 | hypothetical protein |
| GLYMA_03G149700 | NA | protein_coding | Glycine max | 3 | 36.494664 | hypothetical protein |
| GLYMA_03G149800 | 1.03E+08 | protein_coding | Glycine max | 3 | 36.496026 | hypothetical protein |
| GLYMA_03G149900 | 1.01E+08 | protein_coding | Glycine max | 3 | 36.498291 | hypothetical protein |
| GLYMA_03G150000 | NA | protein_coding | Glycine max | 3 | 36.512933 | hypothetical protein |
| GLYMA_03G150100 | NA | protein_coding | Glycine max | 3 | 36.522024 | hypothetical protein |
| GLYMA_03G150200 | NA | protein_coding | Glycine max | 3 | 36.533611 | hypothetical protein |
| GLYMA_03G150300 | NA | protein_coding | Glycine max | 3 | 36.54408 | hypothetical protein |
| GLYMA_03G150400 | NA | protein_coding | Glycine max | 3 | 36.549973 | hypothetical protein |
| GLYMA_03G150500 | NA | protein_coding | Glycine max | 3 | 36.556001 | hypothetical protein |
| GLYMA_03G150600 | NA | protein_coding | Glycine max | 3 | 36.567084 | hypothetical protein |
| GLYMA_03G150700 | 1.01E+08 | protein_coding | Glycine max | 3 | 36.572128 | hypothetical protein |
| GLYMA_03G150800 | 1.01E+08 | protein_coding | Glycine max | 3 | 36.579699 | hypothetical protein |
| GLYMA_03G150900 | 1E+08 | protein_coding | Glycine max | 3 | 36.585397 | hypothetical protein |
| GLYMA_03G151000 | NA | protein_coding | Glycine max | 3 | 36.594584 | hypothetical protein |
| GLYMA_03G151100 | NA | protein_coding | Glycine max | 3 | 36.59752 | hypothetical protein |
| GLYMA_03G151200 | 1.01E+08 | protein_coding | Glycine max | 3 | 36.609344 | hypothetical protein |
| GLYMA_03G151300 | 1.01E+08 | protein_coding | Glycine max | 3 | 36.616173 | hypothetical protein |
| GLYMA_03G151400 | NA | protein_coding | Glycine max | 3 | 36.620828 | hypothetical protein |
| GLYMA_03G151500 | NA | protein_coding | Glycine max | 3 | 36.641782 | hypothetical protein |
| GLYMA_03G151600 | NA | protein_coding | Glycine max | 3 | 36.64687 | hypothetical protein |
| GLYMA_03G151700 | NA | protein_coding | Glycine max | 3 | 36.676659 | hypothetical protein |
| GLYMA_04G125100 | 1.01E+08 | protein_coding | Glycine max | 4 | 16.472994 | hypothetical protein |
| GLYMA_04G125200 | NA | protein_coding | Glycine max | 4 | 16.474623 | hypothetical protein |
| GLYMA_04G125300 | NA | protein_coding | Glycine max | 4 | 16.500908 | hypothetical protein |
| GLYMA_04G125400 | NA | protein_coding | Glycine max | 4 | 16.529335 | hypothetical protein |
| GLYMA_04G125500 | 1.01E+08 | protein_coding | Glycine max | 4 | 16.532221 | hypothetical protein |
| GLYMA_04G125600 | 1.01E+08 | protein_coding | Glycine max | 4 | 16.622379 | hypothetical protein |
| GLYMA_04G125700 | NA | protein_coding | Glycine max | 4 | 16.709512 | hypothetical protein |
| GLYMA_04G125800 | 1.01E+08 | protein_coding | Glycine max | 4 | 16.726595 | hypothetical protein |
| GLYMA_04G125900 | NA | protein_coding | Glycine max | 4 | 16.809159 | hypothetical protein |
| GLYMA_04G126000 | NA | protein_coding | Glycine max | 4 | 16.811865 | hypothetical protein |
| GLYMA_04G126100 | 1.01E+08 | protein_coding | Glycine max | 4 | 16.937746 | hypothetical protein |
| GLYMA_04G198600 | NA | protein_coding | Glycine max | 4 | 47.086335 | hypothetical protein |
| GLYMA_04G198700 | NA | protein_coding | Glycine max | 4 | 47.105887 | hypothetical protein |
| GLYMA_04G198800 | NA | protein_coding | Glycine max | 4 | 47.11266 | hypothetical protein |
| GLYMA_04G198900 | 1.01E+08 | protein_coding | Glycine max | 4 | 47.113595 | hypothetical protein |
| GLYMA_04G199000 | NA | protein_coding | Glycine max | 4 | 47.121567 | hypothetical protein |
| GLYMA_04G199100 | 1.03E+08 | protein_coding | Glycine max | 4 | 47.129399 | hypothetical protein |
| GLYMA_04G199200 | NA | protein_coding | Glycine max | 4 | 47.137301 | hypothetical protein |
| GLYMA_04G199300 | NA | protein_coding | Glycine max | 4 | 47.148346 | hypothetical protein |
| GLYMA_04G199400 | 1.01E+08 | protein_coding | Glycine max | 4 | 47.154987 | hypothetical protein |
| GLYMA_04G199500 | NA | protein_coding | Glycine max | 4 | 47.174367 | hypothetical protein |
| GLYMA_04G199600 | NA | protein_coding | Glycine max | 4 | 47.17883 | hypothetical protein |
| GLYMA_04G199700 | 1.03E+08 | protein_coding | Glycine max | 4 | 47.197472 | hypothetical protein |
| GLYMA_04G199800 | NA | protein_coding | Glycine max | 4 | 47.211288 | hypothetical protein |
| GLYMA_04G199900 | 1.01E+08 | protein_coding | Glycine max | 4 | 47.213255 | hypothetical protein |
| GLYMA_04G200000 | 1.01E+08 | protein_coding | Glycine max | 4 | 47.245072 | hypothetical protein |
| GLYMA_04G200100 | 1.01E+08 | protein_coding | Glycine max | 4 | 47.260385 | hypothetical protein |
| GLYMA_04G200200 | 1.01E+08 | protein_coding | Glycine max | 4 | 47.272565 | hypothetical protein |
| GLYMA_04G200300 | 1.03E+08 | protein_coding | Glycine max | 4 | 47.280047 | hypothetical protein |
| GLYMA_04G200400 | 1.01E+08 | protein_coding | Glycine max | 4 | 47.289746 | hypothetical protein |
| GLYMA_04G200500 | 1E+08 | protein_coding | Glycine max | 4 | 47.311582 | hypothetical protein |
| GLYMA_04G200600 | 1.01E+08 | protein_coding | Glycine max | 4 | 47.316151 | hypothetical protein |
| GLYMA_04G200700 | 1.01E+08 | protein_coding | Glycine max | 4 | 47.34972 | hypothetical protein |
| GLYMA_04G200800 | NA | protein_coding | Glycine max | 4 | 47.352796 | hypothetical protein |
| GLYMA_04G200900 | 1.01E+08 | protein_coding | Glycine max | 4 | 47.363745 | hypothetical protein |
| GLYMA_04G201000 | 1.01E+08 | protein_coding | Glycine max | 4 | 47.375498 | hypothetical protein |
| GLYMA_04G201100 | NA | protein_coding | Glycine max | 4 | 47.397562 | hypothetical protein |
| GLYMA_04G201200 | 1.01E+08 | protein_coding | Glycine max | 4 | 47.403086 | hypothetical protein |
| GLYMA_04G201300 | 1.03E+08 | protein_coding | Glycine max | 4 | 47.421729 | hypothetical protein |
| GLYMA_04G201400 | 1.07E+08 | protein_coding | Glycine max | 4 | 47.430552 | hypothetical protein |
| GLYMA_04G201500 | 1.01E+08 | protein_coding | Glycine max | 4 | 47.439412 | hypothetical protein |
| GLYMA_04G201600 | NA | protein_coding | Glycine max | 4 | 47.454507 | hypothetical protein |
| GLYMA_04G201700 | NA | protein_coding | Glycine max | 4 | 47.460459 | hypothetical protein |
| GLYMA_04G201800 | NA | protein_coding | Glycine max | 4 | 47.465365 | hypothetical protein |
| GLYMA_04G201900 | 1.01E+08 | protein_coding | Glycine max | 4 | 47.469417 | hypothetical protein |
| GLYMA_04G202000 | NA | protein_coding | Glycine max | 4 | 47.480458 | hypothetical protein |
| GLYMA_04G202100 | 1.01E+08 | protein_coding | Glycine max | 4 | 47.491736 | hypothetical protein |
| GLYMA_04G202200 | 1.01E+08 | protein_coding | Glycine max | 4 | 47.502 | hypothetical protein |
| GLYMA_04G202300 | NA | protein_coding | Glycine max | 4 | 47.511762 | hypothetical protein |
| GLYMA_04G202400 | 1.01E+08 | protein_coding | Glycine max | 4 | 47.513446 | hypothetical protein |
| GLYMA_04G202500 | NA | protein_coding | Glycine max | 4 | 47.518247 | hypothetical protein |
| GLYMA_04G202600 | NA | protein_coding | Glycine max | 4 | 47.524459 | hypothetical protein |
| GLYMA_04G202700 | 1.01E+08 | protein_coding | Glycine max | 4 | 47.529084 | hypothetical protein |
| GLYMA_04G202800 | 1.01E+08 | protein_coding | Glycine max | 4 | 47.536842 | hypothetical protein |
| GLYMA_04G202900 | 1.01E+08 | protein_coding | Glycine max | 4 | 47.545779 | hypothetical protein |
| GLYMA_04G203000 | 1.01E+08 | protein_coding | Glycine max | 4 | 47.555168 | hypothetical protein |
| GLYMA_04G203100 | 1.01E+08 | protein_coding | Glycine max | 4 | 47.579726 | hypothetical protein |
| GLYMA_05G031800 | NA | protein_coding | Glycine max | 5 | 2.760249 | hypothetical protein |
| GLYMA_05G031900 | 1.01E+08 | protein_coding | Glycine max | 5 | 2.767384 | hypothetical protein |
| GLYMA_05G032000 | 1.01E+08 | protein_coding | Glycine max | 5 | 2.790581 | hypothetical protein |
| GLYMA_05G032100 | NA | protein_coding | Glycine max | 5 | 2.794636 | hypothetical protein |
| GLYMA_05G032200 | NA | protein_coding | Glycine max | 5 | 2.802638 | hypothetical protein |
| GLYMA_05G032300 | 1.01E+08 | protein_coding | Glycine max | 5 | 2.818779 | hypothetical protein |
| GLYMA_05G032400 | 1.01E+08 | protein_coding | Glycine max | 5 | 2.835362 | hypothetical protein |
| GLYMA_05G032500 | NA | protein_coding | Glycine max | 5 | 2.844488 | hypothetical protein |
| GLYMA_05G032600 | 1.01E+08 | protein_coding | Glycine max | 5 | 2.848695 | hypothetical protein |
| GLYMA_05G032700 | 1.01E+08 | protein_coding | Glycine max | 5 | 2.864935 | hypothetical protein |
| GLYMA_05G032800 | NA | protein_coding | Glycine max | 5 | 2.870616 | hypothetical protein |
| GLYMA_05G032900 | NA | protein_coding | Glycine max | 5 | 2.872504 | hypothetical protein |
| GLYMA_05G033000 | 1.01E+08 | protein_coding | Glycine max | 5 | 2.882073 | hypothetical protein |
| GLYMA_05G033100 | NA | protein_coding | Glycine max | 5 | 2.882782 | hypothetical protein |
| GLYMA_05G033200 | NA | protein_coding | Glycine max | 5 | 2.894024 | hypothetical protein |
| GLYMA_05G033300 | NA | protein_coding | Glycine max | 5 | 2.89958 | hypothetical protein |
| GLYMA_05G033400 | 1.01E+08 | protein_coding | Glycine max | 5 | 2.903718 | hypothetical protein |
| GLYMA_05G033500 | NA | protein_coding | Glycine max | 5 | 2.908499 | hypothetical protein |
| GLYMA_05G033600 | 1.01E+08 | protein_coding | Glycine max | 5 | 2.918077 | hypothetical protein |
| GLYMA_05G033700 | NA | protein_coding | Glycine max | 5 | 2.928085 | hypothetical protein |
| GLYMA_05G033800 | 1.01E+08 | protein_coding | Glycine max | 5 | 2.933201 | hypothetical protein |
| GLYMA_05G033900 | 1.07E+08 | protein_coding | Glycine max | 5 | 2.962201 | hypothetical protein |
| GLYMA_05G034000 | 1.01E+08 | protein_coding | Glycine max | 5 | 2.965889 | hypothetical protein |
| GLYMA_05G034100 | NA | protein_coding | Glycine max | 5 | 2.989078 | hypothetical protein |
| GLYMA_05G034200 | 1.01E+08 | protein_coding | Glycine max | 5 | 2.995359 | hypothetical protein |
| GLYMA_05G034300 | 1.07E+08 | protein_coding | Glycine max | 5 | 3.000206 | hypothetical protein |
| GLYMA_05G034400 | NA | protein_coding | Glycine max | 5 | 3.002113 | hypothetical protein |
| GLYMA_05G034500 | NA | protein_coding | Glycine max | 5 | 3.021697 | hypothetical protein |
| GLYMA_05G034600 | 1.01E+08 | protein_coding | Glycine max | 5 | 3.036859 | hypothetical protein |
| GLYMA_05G034700 | 1.01E+08 | protein_coding | Glycine max | 5 | 3.046414 | hypothetical protein |
| GLYMA_05G034800 | 1.01E+08 | protein_coding | Glycine max | 5 | 3.052781 | hypothetical protein |
| GLYMA_05G034900 | NA | protein_coding | Glycine max | 5 | 3.074078 | hypothetical protein |
| GLYMA_05G035000 | 1.01E+08 | protein_coding | Glycine max | 5 | 3.078012 | hypothetical protein |
| GLYMA_05G035100 | 1.01E+08 | protein_coding | Glycine max | 5 | 3.096999 | hypothetical protein |
| GLYMA_05G035200 | NA | protein_coding | Glycine max | 5 | 3.105274 | hypothetical protein |
| GLYMA_05G035300 | 1.01E+08 | protein_coding | Glycine max | 5 | 3.113055 | hypothetical protein |
| GLYMA_05G035400 | NA | protein_coding | Glycine max | 5 | 3.118491 | hypothetical protein |
| GLYMA_05G035500 | NA | protein_coding | Glycine max | 5 | 3.123293 | hypothetical protein |
| GLYMA_05G035600 | 1.01E+08 | protein_coding | Glycine max | 5 | 3.129627 | hypothetical protein |
| GLYMA_05G035700 | 1.01E+08 | protein_coding | Glycine max | 5 | 3.138855 | hypothetical protein |
| GLYMA_05G035800 | 1.01E+08 | protein_coding | Glycine max | 5 | 3.151303 | hypothetical protein |
| GLYMA_05G035900 | 1.01E+08 | protein_coding | Glycine max | 5 | 3.162908 | hypothetical protein |
| GLYMA_05G036000 | 1.03E+08 | protein_coding | Glycine max | 5 | 3.171382 | hypothetical protein |
| GLYMA_05G036100 | 1.01E+08 | protein_coding | Glycine max | 5 | 3.177057 | hypothetical protein |
| GLYMA_05G036200 | NA | protein_coding | Glycine max | 5 | 3.18673 | hypothetical protein |
| GLYMA_05G036300 | 1.01E+08 | protein_coding | Glycine max | 5 | 3.189678 | hypothetical protein |
| GLYMA_05G036400 | NA | protein_coding | Glycine max | 5 | 3.200781 | hypothetical protein |
| GLYMA_05G036500 | 1.01E+08 | protein_coding | Glycine max | 5 | 3.209765 | hypothetical protein |
| GLYMA_05G036600 | 1.01E+08 | protein_coding | Glycine max | 5 | 3.214335 | hypothetical protein |
| GLYMA_05G036700 | NA | protein_coding | Glycine max | 5 | 3.224675 | hypothetical protein |
| GLYMA_05G036800 | 1.01E+08 | protein_coding | Glycine max | 5 | 3.241818 | hypothetical protein |
| GLYMA_05G036900 | 1.01E+08 | protein_coding | Glycine max | 5 | 3.260922 | hypothetical protein |
| GLYMA_05G215900 | 1.01E+08 | protein_coding | Glycine max | 5 | 39.636109 | hypothetical protein |
| GLYMA_05G216000 | NA | protein_coding | Glycine max | 5 | 39.643274 | hypothetical protein |
| GLYMA_05G216100 | 1.01E+08 | protein_coding | Glycine max | 5 | 39.646634 | hypothetical protein |
| GLYMA_05G216200 | 1.01E+08 | protein_coding | Glycine max | 5 | 39.648138 | hypothetical protein |
| GLYMA_05G216300 | 1.03E+08 | protein_coding | Glycine max | 5 | 39.653906 | hypothetical protein |
| GLYMA_05G216400 | 732598 | protein_coding | Glycine max | 5 | 39.673239 | hypothetical protein |
| GLYMA_05G216500 | 1.01E+08 | protein_coding | Glycine max | 5 | 39.681205 | hypothetical protein |
| GLYMA_05G216600 | 1.01E+08 | protein_coding | Glycine max | 5 | 39.686415 | hypothetical protein |
| GLYMA_05G216700 | 1.01E+08 | protein_coding | Glycine max | 5 | 39.696647 | hypothetical protein |
| GLYMA_05G216800 | 1.03E+08 | protein_coding | Glycine max | 5 | 39.701247 | hypothetical protein |
| GLYMA_05G216900 | 1.03E+08 | protein_coding | Glycine max | 5 | 39.713295 | hypothetical protein |
| GLYMA_05G217000 | 1.01E+08 | protein_coding | Glycine max | 5 | 39.719507 | hypothetical protein |
| GLYMA_05G217100 | 1.01E+08 | protein_coding | Glycine max | 5 | 39.735139 | hypothetical protein |
| GLYMA_05G217200 | NA | protein_coding | Glycine max | 5 | 39.740133 | hypothetical protein |
| GLYMA_05G217300 | NA | protein_coding | Glycine max | 5 | 39.74436 | hypothetical protein |
| GLYMA_05G217400 | 1.01E+08 | protein_coding | Glycine max | 5 | 39.75243 | hypothetical protein |
| GLYMA_05G217500 | 1.01E+08 | protein_coding | Glycine max | 5 | 39.757555 | hypothetical protein |
| GLYMA_05G217600 | 1.01E+08 | protein_coding | Glycine max | 5 | 39.762157 | hypothetical protein |
| GLYMA_05G217700 | 1.01E+08 | protein_coding | Glycine max | 5 | 39.77022 | hypothetical protein |
| GLYMA_05G217800 | 1.01E+08 | protein_coding | Glycine max | 5 | 39.780508 | hypothetical protein |
| GLYMA_05G217900 | 1.01E+08 | protein_coding | Glycine max | 5 | 39.797657 | hypothetical protein |
| GLYMA_05G218000 | 1.01E+08 | protein_coding | Glycine max | 5 | 39.801851 | hypothetical protein |
| GLYMA_05G218100 | NA | protein_coding | Glycine max | 5 | 39.807065 | hypothetical protein |
| GLYMA_05G218200 | NA | protein_coding | Glycine max | 5 | 39.810698 | hypothetical protein |
| GLYMA_05G218300 | NA | protein_coding | Glycine max | 5 | 39.815332 | hypothetical protein |
| GLYMA_05G218400 | 1.01E+08 | protein_coding | Glycine max | 5 | 39.820569 | hypothetical protein |
| GLYMA_05G218500 | 1.01E+08 | protein_coding | Glycine max | 5 | 39.823269 | hypothetical protein |
| GLYMA_05G218600 | NA | protein_coding | Glycine max | 5 | 39.83008 | hypothetical protein |
| GLYMA_05G218700 | 1.01E+08 | protein_coding | Glycine max | 5 | 39.838913 | hypothetical protein |
| GLYMA_05G218800 | 1.01E+08 | protein_coding | Glycine max | 5 | 39.846073 | hypothetical protein |
| GLYMA_05G218900 | 1.03E+08 | protein_coding | Glycine max | 5 | 39.849879 | hypothetical protein |
| GLYMA_05G219000 | 1.01E+08 | protein_coding | Glycine max | 5 | 39.8611 | hypothetical protein |
| GLYMA_05G219100 | NA | protein_coding | Glycine max | 5 | 39.869675 | hypothetical protein |
| GLYMA_05G219200 | 1.01E+08 | protein_coding | Glycine max | 5 | 39.875137 | hypothetical protein |
| GLYMA_05G219300 | 1.01E+08 | protein_coding | Glycine max | 5 | 39.881909 | hypothetical protein |
| GLYMA_05G219400 | NA | protein_coding | Glycine max | 5 | 39.891624 | hypothetical protein |
| GLYMA_05G219500 | 1.01E+08 | protein_coding | Glycine max | 5 | 39.896794 | hypothetical protein |
| GLYMA_05G219600 | NA | protein_coding | Glycine max | 5 | 39.90798 | hypothetical protein |
| GLYMA_05G219700 | 1.01E+08 | protein_coding | Glycine max | 5 | 39.914335 | hypothetical protein |
| GLYMA_05G219800 | NA | protein_coding | Glycine max | 5 | 39.920183 | hypothetical protein |
| GLYMA_05G219900 | NA | protein_coding | Glycine max | 5 | 39.931649 | hypothetical protein |
| GLYMA_05G220000 | 1.01E+08 | protein_coding | Glycine max | 5 | 39.939315 | hypothetical protein |
| GLYMA_05G220100 | 1.01E+08 | protein_coding | Glycine max | 5 | 39.950499 | hypothetical protein |
| GLYMA_05G220200 | 1.01E+08 | protein_coding | Glycine max | 5 | 39.956901 | hypothetical protein |
| GLYMA_05G220300 | 1.01E+08 | protein_coding | Glycine max | 5 | 39.974035 | hypothetical protein |
| GLYMA_05G220400 | NA | protein_coding | Glycine max | 5 | 40.002163 | hypothetical protein |
| GLYMA_05G220500 | 1.01E+08 | protein_coding | Glycine max | 5 | 40.008032 | hypothetical protein |
| GLYMA_05G220600 | 1.01E+08 | protein_coding | Glycine max | 5 | 40.016495 | hypothetical protein |
| GLYMA_05G220700 | 1.01E+08 | protein_coding | Glycine max | 5 | 40.022979 | hypothetical protein |
| GLYMA_05G220800 | NA | protein_coding | Glycine max | 5 | 40.034916 | hypothetical protein |
| GLYMA_05G220900 | 1.01E+08 | protein_coding | Glycine max | 5 | 40.040654 | hypothetical protein |
| GLYMA_05G221000 | 1.01E+08 | protein_coding | Glycine max | 5 | 40.045658 | hypothetical protein |
| GLYMA_05G221100 | 547498 | protein_coding | Glycine max | 5 | 40.049855 | hypothetical protein |
| GLYMA_05G221200 | NA | protein_coding | Glycine max | 5 | 40.060336 | hypothetical protein |
| GLYMA_05G221300 | 1.01E+08 | protein_coding | Glycine max | 5 | 40.073989 | hypothetical protein |
| GLYMA_05G221400 | 1.01E+08 | protein_coding | Glycine max | 5 | 40.094498 | hypothetical protein |
| GLYMA_05G221500 | 1.01E+08 | protein_coding | Glycine max | 5 | 40.096703 | hypothetical protein |
| GLYMA_05G221600 | NA | protein_coding | Glycine max | 5 | 40.107183 | hypothetical protein |
| GLYMA_05G221700 | 1.01E+08 | protein_coding | Glycine max | 5 | 40.115849 | hypothetical protein |
| GLYMA_05G221800 | NA | protein_coding | Glycine max | 5 | 40.120284 | hypothetical protein |
| GLYMA_05G221900 | 1.01E+08 | protein_coding | Glycine max | 5 | 40.13488 | hypothetical protein |
| GLYMA_06G119200 | NA | protein_coding | Glycine max | 6 | 9.706114 | hypothetical protein |
| GLYMA_06G119300 | 1.01E+08 | protein_coding | Glycine max | 6 | 9.711392 | hypothetical protein |
| GLYMA_06G119400 | 1.01E+08 | protein_coding | Glycine max | 6 | 9.719172 | hypothetical protein |
| GLYMA_06G119500 | NA | protein_coding | Glycine max | 6 | 9.727943 | hypothetical protein |
| GLYMA_06G119600 | NA | protein_coding | Glycine max | 6 | 9.735603 | hypothetical protein |
| GLYMA_06G119700 | 1.01E+08 | protein_coding | Glycine max | 6 | 9.737042 | hypothetical protein |
| GLYMA_06G119800 | 1.01E+08 | protein_coding | Glycine max | 6 | 9.750547 | hypothetical protein |
| GLYMA_06G119900 | NA | protein_coding | Glycine max | 6 | 9.765201 | hypothetical protein |
| GLYMA_06G120000 | NA | protein_coding | Glycine max | 6 | 9.767737 | hypothetical protein |
| GLYMA_06G120100 | NA | protein_coding | Glycine max | 6 | 9.770109 | hypothetical protein |
| GLYMA_06G120200 | 1.01E+08 | protein_coding | Glycine max | 6 | 9.774043 | hypothetical protein |
| GLYMA_06G120300 | 1.01E+08 | protein_coding | Glycine max | 6 | 9.778588 | hypothetical protein |
| GLYMA_06G120400 | 1.01E+08 | protein_coding | Glycine max | 6 | 9.786617 | hypothetical protein |
| GLYMA_06G120500 | 1.01E+08 | protein_coding | Glycine max | 6 | 9.802985 | hypothetical protein |
| GLYMA_06G120600 | 1.01E+08 | protein_coding | Glycine max | 6 | 9.821042 | hypothetical protein |
| GLYMA_06G120700 | 1.01E+08 | protein_coding | Glycine max | 6 | 9.833598 | hypothetical protein |
| GLYMA_06G120800 | NA | protein_coding | Glycine max | 6 | 9.83969 | hypothetical protein |
| GLYMA_06G120900 | NA | protein_coding | Glycine max | 6 | 9.846148 | hypothetical protein |
| GLYMA_06G121000 | NA | protein_coding | Glycine max | 6 | 9.848464 | hypothetical protein |
| GLYMA_06G121100 | 1E+08 | protein_coding | Glycine max | 6 | 9.856604 | hypothetical protein |
| GLYMA_06G121200 | 1.01E+08 | protein_coding | Glycine max | 6 | 9.867126 | hypothetical protein |
| GLYMA_06G121300 | 1.01E+08 | protein_coding | Glycine max | 6 | 9.883992 | hypothetical protein |
| GLYMA_06G121400 | 1E+08 | protein_coding | Glycine max | 6 | 9.890758 | hypothetical protein |
| GLYMA_06G121500 | 1.01E+08 | protein_coding | Glycine max | 6 | 9.892482 | hypothetical protein |
| GLYMA_06G121600 | NA | protein_coding | Glycine max | 6 | 9.927411 | hypothetical protein |
| GLYMA_06G121700 | 1.03E+08 | protein_coding | Glycine max | 6 | 9.932544 | hypothetical protein |
| GLYMA_06G121800 | NA | protein_coding | Glycine max | 6 | 9.938429 | hypothetical protein |
| GLYMA_06G121900 | NA | protein_coding | Glycine max | 6 | 9.944421 | hypothetical protein |
| GLYMA_06G122000 | NA | protein_coding | Glycine max | 6 | 9.953495 | hypothetical protein |
| GLYMA_06G122100 | NA | protein_coding | Glycine max | 6 | 9.956928 | hypothetical protein |
| GLYMA_06G122200 | 1.01E+08 | protein_coding | Glycine max | 6 | 9.966473 | hypothetical protein |
| GLYMA_06G122300 | 1.01E+08 | protein_coding | Glycine max | 6 | 9.979512 | hypothetical protein |
| GLYMA_06G122400 | 1.01E+08 | protein_coding | Glycine max | 6 | 9.98147 | hypothetical protein |
| GLYMA_06G122500 | 1.01E+08 | protein_coding | Glycine max | 6 | 9.987698 | hypothetical protein |
| GLYMA_06G122600 | 1.01E+08 | protein_coding | Glycine max | 6 | 9.998469 | hypothetical protein |
| GLYMA_06G122700 | 1.01E+08 | protein_coding | Glycine max | 6 | 10.00229 | hypothetical protein |
| GLYMA_06G122800 | 1.01E+08 | protein_coding | Glycine max | 6 | 10.009186 | hypothetical protein |
| GLYMA_06G122900 | NA | protein_coding | Glycine max | 6 | 10.016593 | hypothetical protein |
| GLYMA_06G123000 | 1.01E+08 | protein_coding | Glycine max | 6 | 10.020449 | hypothetical protein |
| GLYMA_06G123100 | 1.01E+08 | protein_coding | Glycine max | 6 | 10.029823 | hypothetical protein |
| GLYMA_06G123200 | 1.01E+08 | protein_coding | Glycine max | 6 | 10.039273 | hypothetical protein |
| GLYMA_06G123300 | NA | protein_coding | Glycine max | 6 | 10.045565 | hypothetical protein |
| GLYMA_06G123400 | 1.01E+08 | protein_coding | Glycine max | 6 | 10.052176 | hypothetical protein |
| GLYMA_06G123500 | 1.01E+08 | protein_coding | Glycine max | 6 | 10.057556 | hypothetical protein |
| GLYMA_06G123600 | NA | protein_coding | Glycine max | 6 | 10.066855 | hypothetical protein |
| GLYMA_06G123700 | 1.01E+08 | protein_coding | Glycine max | 6 | 10.070322 | hypothetical protein |
| GLYMA_06G123800 | 1.01E+08 | protein_coding | Glycine max | 6 | 10.070358 | hypothetical protein |
| GLYMA_06G123900 | 1.01E+08 | protein_coding | Glycine max | 6 | 10.080152 | hypothetical protein |
| GLYMA_06G124000 | 1.01E+08 | protein_coding | Glycine max | 6 | 10.083602 | hypothetical protein |
| GLYMA_06G124100 | 1.01E+08 | protein_coding | Glycine max | 6 | 10.090051 | hypothetical protein |
| GLYMA_06G124200 | 1.01E+08 | protein_coding | Glycine max | 6 | 10.09871 | hypothetical protein |
| GLYMA_06G124300 | 1.01E+08 | protein_coding | Glycine max | 6 | 10.113576 | hypothetical protein |
| GLYMA_06G124400 | NA | protein_coding | Glycine max | 6 | 10.128263 | hypothetical protein |
| GLYMA_06G124500 | 1.01E+08 | protein_coding | Glycine max | 6 | 10.136573 | hypothetical protein |
| GLYMA_06G124600 | 1.01E+08 | protein_coding | Glycine max | 6 | 10.142607 | hypothetical protein |
| GLYMA_06G124700 | 1.01E+08 | protein_coding | Glycine max | 6 | 10.156836 | hypothetical protein |
| GLYMA_06G124800 | 1.01E+08 | protein_coding | Glycine max | 6 | 10.164556 | hypothetical protein |
| GLYMA_06G124900 | 1E+08 | protein_coding | Glycine max | 6 | 10.168532 | hypothetical protein |
| GLYMA_06G125000 | NA | protein_coding | Glycine max | 6 | 10.16902 | hypothetical protein |
| GLYMA_06G125100 | 1.01E+08 | protein_coding | Glycine max | 6 | 10.190469 | hypothetical protein |
| GLYMA_06G125200 | NA | protein_coding | Glycine max | 6 | 10.209787 | hypothetical protein |
| GLYMA_06G125300 | NA | protein_coding | Glycine max | 6 | 10.216506 | hypothetical protein |
| GLYMA_06G125400 | NA | protein_coding | Glycine max | 6 | 10.219813 | hypothetical protein |
| GLYMA_06G125500 | 1.01E+08 | protein_coding | Glycine max | 6 | 10.239633 | hypothetical protein |
| GLYMA_06G125600 | 1.01E+08 | protein_coding | Glycine max | 6 | 10.247993 | hypothetical protein |
| GLYMA_06G125700 | NA | protein_coding | Glycine max | 6 | 10.255309 | hypothetical protein |
| GLYMA_06G125800 | NA | protein_coding | Glycine max | 6 | 10.274916 | hypothetical protein |
| GLYMA_06G125900 | 1.01E+08 | protein_coding | Glycine max | 6 | 10.286061 | hypothetical protein |
| GLYMA_06G126000 | NA | protein_coding | Glycine max | 6 | 10.293511 | hypothetical protein |
| GLYMA_06G126100 | NA | protein_coding | Glycine max | 6 | 10.315437 | hypothetical protein |
| GLYMA_06G126200 | NA | protein_coding | Glycine max | 6 | 10.329274 | hypothetical protein |
| GLYMA_06G126300 | 1.01E+08 | protein_coding | Glycine max | 6 | 10.331312 | hypothetical protein |
| GLYMA_06G126400 | 1.01E+08 | protein_coding | Glycine max | 6 | 10.35665 | hypothetical protein |
| GLYMA_06G126500 | 1.01E+08 | protein_coding | Glycine max | 6 | 10.367536 | hypothetical protein |
| GLYMA_06G126600 | 1.01E+08 | protein_coding | Glycine max | 6 | 10.376446 | hypothetical protein |
| GLYMA_06G126700 | 1.01E+08 | protein_coding | Glycine max | 6 | 10.384771 | hypothetical protein |
| GLYMA_06G147600 | NA | protein_coding | Glycine max | 6 | 12.052122 | hypothetical protein |
| GLYMA_06G147700 | NA | protein_coding | Glycine max | 6 | 12.068339 | hypothetical protein |
| GLYMA_06G147800 | 1.01E+08 | protein_coding | Glycine max | 6 | 12.070691 | hypothetical protein |
| GLYMA_06G147900 | NA | protein_coding | Glycine max | 6 | 12.081868 | hypothetical protein |
| GLYMA_06G148000 | NA | protein_coding | Glycine max | 6 | 12.089141 | hypothetical protein |
| GLYMA_06G148100 | NA | protein_coding | Glycine max | 6 | 12.09893 | hypothetical protein |
| GLYMA_06G148200 | NA | protein_coding | Glycine max | 6 | 12.108718 | hypothetical protein |
| GLYMA_06G148300 | 1.01E+08 | protein_coding | Glycine max | 6 | 12.113168 | hypothetical protein |
| GLYMA_06G148400 | 1.01E+08 | protein_coding | Glycine max | 6 | 12.120448 | hypothetical protein |
| GLYMA_06G148500 | NA | protein_coding | Glycine max | 6 | 12.136375 | hypothetical protein |
| GLYMA_06G148600 | 1.01E+08 | protein_coding | Glycine max | 6 | 12.14395 | hypothetical protein |
| GLYMA_06G148700 | 1.01E+08 | protein_coding | Glycine max | 6 | 12.149262 | hypothetical protein |
| GLYMA_06G148800 | 1.01E+08 | protein_coding | Glycine max | 6 | 12.157484 | hypothetical protein |
| GLYMA_06G148900 | NA | protein_coding | Glycine max | 6 | 12.16154 | hypothetical protein |
| GLYMA_06G149000 | NA | protein_coding | Glycine max | 6 | 12.1662 | hypothetical protein |
| GLYMA_06G149100 | NA | protein_coding | Glycine max | 6 | 12.182441 | hypothetical protein |
| GLYMA_06G149200 | 1.01E+08 | protein_coding | Glycine max | 6 | 12.198024 | hypothetical protein |
| GLYMA_06G149300 | NA | protein_coding | Glycine max | 6 | 12.201964 | hypothetical protein |
| GLYMA_06G149400 | NA | protein_coding | Glycine max | 6 | 12.205536 | hypothetical protein |
| GLYMA_06G149500 | 1.01E+08 | protein_coding | Glycine max | 6 | 12.215002 | hypothetical protein |
| GLYMA_06G149600 | 1.01E+08 | protein_coding | Glycine max | 6 | 12.221078 | hypothetical protein |
| GLYMA_06G149700 | NA | protein_coding | Glycine max | 6 | 12.229701 | hypothetical protein |
| GLYMA_06G149800 | NA | protein_coding | Glycine max | 6 | 12.234973 | hypothetical protein |
| GLYMA_06G149900 | 1.01E+08 | protein_coding | Glycine max | 6 | 12.238755 | hypothetical protein |
| GLYMA_06G150000 | 1.01E+08 | protein_coding | Glycine max | 6 | 12.251082 | hypothetical protein |
| GLYMA_06G150100 | NA | protein_coding | Glycine max | 6 | 12.256574 | hypothetical protein |
| GLYMA_06G150200 | NA | protein_coding | Glycine max | 6 | 12.265549 | hypothetical protein |
| GLYMA_06G150300 | 1E+08 | protein_coding | Glycine max | 6 | 12.266504 | hypothetical protein |
| GLYMA_06G150400 | NA | protein_coding | Glycine max | 6 | 12.282405 | hypothetical protein |
| GLYMA_06G150500 | NA | protein_coding | Glycine max | 6 | 12.283794 | hypothetical protein |
| GLYMA_06G150600 | NA | protein_coding | Glycine max | 6 | 12.289583 | hypothetical protein |
| GLYMA_06G150700 | NA | protein_coding | Glycine max | 6 | 12.289992 | hypothetical protein |
| GLYMA_06G150800 | NA | protein_coding | Glycine max | 6 | 12.29407 | hypothetical protein |
| GLYMA_06G150900 | 1E+08 | protein_coding | Glycine max | 6 | 12.299926 | hypothetical protein |
| GLYMA_06G151000 | NA | protein_coding | Glycine max | 6 | 12.299958 | hypothetical protein |
| GLYMA_06G151100 | NA | protein_coding | Glycine max | 6 | 12.308196 | hypothetical protein |
| GLYMA_06G151200 | 1.01E+08 | protein_coding | Glycine max | 6 | 12.318784 | hypothetical protein |
| GLYMA_06G151300 | 1.01E+08 | protein_coding | Glycine max | 6 | 12.334323 | hypothetical protein |
| GLYMA_06G151400 | 1.01E+08 | protein_coding | Glycine max | 6 | 12.350255 | hypothetical protein |
| GLYMA_06G151500 | 1.01E+08 | protein_coding | Glycine max | 6 | 12.360716 | hypothetical protein |
| GLYMA_06G151600 | NA | protein_coding | Glycine max | 6 | 12.367665 | hypothetical protein |
| GLYMA_06G151700 | 1E+08 | protein_coding | Glycine max | 6 | 12.368777 | hypothetical protein |
| GLYMA_06G151800 | NA | protein_coding | Glycine max | 6 | 12.371794 | hypothetical protein |
| GLYMA_06G151900 | 1.01E+08 | protein_coding | Glycine max | 6 | 12.375941 | hypothetical protein |
| GLYMA_06G152000 | NA | protein_coding | Glycine max | 6 | 12.38437 | hypothetical protein |
| GLYMA_06G152100 | NA | protein_coding | Glycine max | 6 | 12.384666 | hypothetical protein |
| GLYMA_06G152200 | 1.01E+08 | protein_coding | Glycine max | 6 | 12.389623 | hypothetical protein |
| GLYMA_06G152300 | 1.01E+08 | protein_coding | Glycine max | 6 | 12.409701 | hypothetical protein |
| GLYMA_06G152400 | 1.01E+08 | protein_coding | Glycine max | 6 | 12.418934 | hypothetical protein |
| GLYMA_06G152500 | 1.01E+08 | protein_coding | Glycine max | 6 | 12.424219 | hypothetical protein |
| GLYMA_06G152600 | 1.01E+08 | protein_coding | Glycine max | 6 | 12.433797 | hypothetical protein |
| GLYMA_06G152700 | 1.01E+08 | protein_coding | Glycine max | 6 | 12.447761 | hypothetical protein |
| GLYMA_06G152800 | 1.01E+08 | protein_coding | Glycine max | 6 | 12.458044 | hypothetical protein |
| GLYMA_06G152900 | 1E+08 | protein_coding | Glycine max | 6 | 12.478823 | hypothetical protein |
| GLYMA_06G153000 | 1.01E+08 | protein_coding | Glycine max | 6 | 12.486501 | hypothetical protein |
| GLYMA_06G153100 | NA | protein_coding | Glycine max | 6 | 12.495442 | hypothetical protein |
| GLYMA_06G153200 | 1.01E+08 | protein_coding | Glycine max | 6 | 12.517434 | hypothetical protein |
| GLYMA_06G153300 | 1.01E+08 | protein_coding | Glycine max | 6 | 12.523802 | hypothetical protein |
| GLYMA_06G153600 | 1.01E+08 | protein_coding | Glycine max | 6 | 12.533507 | hypothetical protein |
| GLYMA_06G267800 | NA | protein_coding | Glycine max | 6 | 45.642958 | hypothetical protein |
| GLYMA_06G267900 | NA | protein_coding | Glycine max | 6 | 45.643739 | hypothetical protein |
| GLYMA_06G268000 | NA | protein_coding | Glycine max | 6 | 45.647929 | hypothetical protein |
| GLYMA_06G268100 | 1.01E+08 | protein_coding | Glycine max | 6 | 45.65742 | hypothetical protein |
| GLYMA_06G268200 | NA | protein_coding | Glycine max | 6 | 45.674556 | hypothetical protein |
| GLYMA_06G268300 | NA | protein_coding | Glycine max | 6 | 45.677814 | hypothetical protein |
| GLYMA_06G268400 | NA | protein_coding | Glycine max | 6 | 45.694175 | hypothetical protein |
| GLYMA_06G268500 | NA | protein_coding | Glycine max | 6 | 45.695883 | hypothetical protein |
| GLYMA_06G268600 | 1.01E+08 | protein_coding | Glycine max | 6 | 45.72161 | hypothetical protein |
| GLYMA_06G268700 | 1.01E+08 | protein_coding | Glycine max | 6 | 45.7254 | hypothetical protein |
| GLYMA_06G268800 | NA | protein_coding | Glycine max | 6 | 45.753991 | hypothetical protein |
| GLYMA_06G268900 | NA | protein_coding | Glycine max | 6 | 45.755827 | hypothetical protein |
| GLYMA_06G269000 | 1.01E+08 | protein_coding | Glycine max | 6 | 45.76323 | hypothetical protein |
| GLYMA_06G269100 | NA | protein_coding | Glycine max | 6 | 45.792877 | hypothetical protein |
| GLYMA_06G269200 | NA | protein_coding | Glycine max | 6 | 45.80226 | hypothetical protein |
| GLYMA_06G269300 | 1.01E+08 | protein_coding | Glycine max | 6 | 45.811991 | hypothetical protein |
| GLYMA_06G269400 | NA | protein_coding | Glycine max | 6 | 45.821105 | hypothetical protein |
| GLYMA_06G269500 | 1.01E+08 | protein_coding | Glycine max | 6 | 45.829139 | hypothetical protein |
| GLYMA_06G269600 | 1E+08 | protein_coding | Glycine max | 6 | 45.836684 | hypothetical protein |
| GLYMA_06G269700 | 547461 | protein_coding | Glycine max | 6 | 45.848185 | hypothetical protein |
| GLYMA_06G269800 | NA | protein_coding | Glycine max | 6 | 45.862997 | hypothetical protein |
| GLYMA_06G269900 | 1.01E+08 | protein_coding | Glycine max | 6 | 45.878059 | hypothetical protein |
| GLYMA_06G270000 | NA | protein_coding | Glycine max | 6 | 45.899093 | hypothetical protein |
| GLYMA_06G270100 | NA | protein_coding | Glycine max | 6 | 45.90169 | hypothetical protein |
| GLYMA_06G270200 | 1.01E+08 | protein_coding | Glycine max | 6 | 45.907256 | hypothetical protein |
| GLYMA_06G270300 | 1.01E+08 | protein_coding | Glycine max | 6 | 45.921677 | hypothetical protein |
| GLYMA_06G270400 | 1.01E+08 | protein_coding | Glycine max | 6 | 45.961564 | hypothetical protein |
| GLYMA_06G270500 | NA | protein_coding | Glycine max | 6 | 45.968696 | hypothetical protein |
| GLYMA_06G270600 | NA | protein_coding | Glycine max | 6 | 45.981416 | hypothetical protein |
| GLYMA_06G270700 | 1.01E+08 | protein_coding | Glycine max | 6 | 45.984804 | hypothetical protein |
| GLYMA_06G270800 | 1.01E+08 | protein_coding | Glycine max | 6 | 46.00336 | hypothetical protein |
| GLYMA_06G270900 | NA | protein_coding | Glycine max | 6 | 46.007621 | hypothetical protein |
| GLYMA_06G271000 | 1.01E+08 | protein_coding | Glycine max | 6 | 46.016701 | hypothetical protein |
| GLYMA_06G271100 | NA | protein_coding | Glycine max | 6 | 46.084047 | hypothetical protein |
| GLYMA_06G271200 | 1.01E+08 | protein_coding | Glycine max | 6 | 46.100462 | hypothetical protein |
| GLYMA_06G271300 | NA | protein_coding | Glycine max | 6 | 46.131735 | hypothetical protein |
| GLYMA_06G271400 | 1.01E+08 | protein_coding | Glycine max | 6 | 46.134359 | hypothetical protein |
| GLYMA_06G319400 | NA | protein_coding | Glycine max | 6 | 50.825005 | hypothetical protein |
| GLYMA_06G319500 | 1.01E+08 | protein_coding | Glycine max | 6 | 50.82893 | hypothetical protein |
| GLYMA_06G319600 | NA | protein_coding | Glycine max | 6 | 50.850357 | hypothetical protein |
| GLYMA_06G319700 | 1E+08 | protein_coding | Glycine max | 6 | 50.856253 | hypothetical protein |
| GLYMA_06G319800 | 1.01E+08 | protein_coding | Glycine max | 6 | 50.861494 | hypothetical protein |
| GLYMA_06G319900 | 1.01E+08 | protein_coding | Glycine max | 6 | 50.865413 | hypothetical protein |
| GLYMA_06G320000 | 1.01E+08 | protein_coding | Glycine max | 6 | 50.870241 | hypothetical protein |
| GLYMA_06G320100 | 1.01E+08 | protein_coding | Glycine max | 6 | 50.874167 | hypothetical protein |
| GLYMA_06G320200 | 1.01E+08 | protein_coding | Glycine max | 6 | 50.893586 | hypothetical protein |
| GLYMA_06G320300 | 1.01E+08 | protein_coding | Glycine max | 6 | 50.899704 | hypothetical protein |
| GLYMA_06G320400 | 1.01E+08 | protein_coding | Glycine max | 6 | 50.905026 | hypothetical protein |
| GLYMA_06G320500 | 1.01E+08 | protein_coding | Glycine max | 6 | 50.915085 | hypothetical protein |
| GLYMA_06G320600 | 1.01E+08 | protein_coding | Glycine max | 6 | 50.922019 | hypothetical protein |
| GLYMA_06G320700 | 1E+08 | protein_coding | Glycine max | 6 | 50.942839 | hypothetical protein |
| GLYMA_06G320800 | 1.01E+08 | protein_coding | Glycine max | 6 | 50.951025 | hypothetical protein |
| GLYMA_06G320900 | 1.01E+08 | protein_coding | Glycine max | 6 | 50.957993 | hypothetical protein |
| GLYMA_06G321000 | NA | protein_coding | Glycine max | 6 | 50.974137 | hypothetical protein |
| GLYMA_06G321100 | NA | protein_coding | Glycine max | 6 | 50.984727 | hypothetical protein |
| GLYMA_06G321200 | 1.01E+08 | protein_coding | Glycine max | 6 | 50.989288 | hypothetical protein |
| GLYMA_06G321400 | 1.01E+08 | protein_coding | Glycine max | 6 | 51.001786 | hypothetical protein |
| GLYMA_06G321500 | NA | protein_coding | Glycine max | 6 | 51.007221 | hypothetical protein |
| GLYMA_06G321600 | NA | protein_coding | Glycine max | 6 | 51.023725 | hypothetical protein |
| GLYMA_06G321700 | NA | protein_coding | Glycine max | 6 | 51.03003 | hypothetical protein |
| GLYMA_06G321800 | 1.01E+08 | protein_coding | Glycine max | 6 | 51.037094 | hypothetical protein |
| GLYMA_06G321900 | 1E+08 | protein_coding | Glycine max | 6 | 51.040796 | hypothetical protein |
| GLYMA_06G322000 | 1.01E+08 | protein_coding | Glycine max | 6 | 51.042976 | hypothetical protein |
| GLYMA_06G322100 | 1E+08 | protein_coding | Glycine max | 6 | 51.046118 | hypothetical protein |
| GLYMA_06G322200 | 1.01E+08 | protein_coding | Glycine max | 6 | 51.048637 | hypothetical protein |
| GLYMA_06G322300 | 1.01E+08 | protein_coding | Glycine max | 6 | 51.058001 | hypothetical protein |
| GLYMA_06G322400 | NA | protein_coding | Glycine max | 6 | 51.063411 | hypothetical protein |
| GLYMA_06G322500 | 1.01E+08 | protein_coding | Glycine max | 6 | 51.070623 | hypothetical protein |
| GLYMA_06G322600 | 1.01E+08 | protein_coding | Glycine max | 6 | 51.075708 | hypothetical protein |
| GLYMA_06G322700 | 1.01E+08 | protein_coding | Glycine max | 6 | 51.094342 | hypothetical protein |
| GLYMA_06G322800 | 1.01E+08 | protein_coding | Glycine max | 6 | 51.099316 | hypothetical protein |
| GLYMA_06G322900 | NA | protein_coding | Glycine max | 6 | 51.102118 | hypothetical protein |
| GLYMA_06G323000 | 1.01E+08 | protein_coding | Glycine max | 6 | 51.103666 | hypothetical protein |
| GLYMA_06G323100 | 1.01E+08 | protein_coding | Glycine max | 6 | 51.107875 | hypothetical protein |
| GLYMA_06G323200 | 1.01E+08 | protein_coding | Glycine max | 6 | 51.114431 | hypothetical protein |
| GLYMA_06G323300 | NA | protein_coding | Glycine max | 6 | 51.119276 | hypothetical protein |
| GLYMA_06G323400 | 1.01E+08 | protein_coding | Glycine max | 6 | 51.123105 | hypothetical protein |
| GLYMA_06G323500 | NA | protein_coding | Glycine max | 6 | 51.130388 | hypothetical protein |
| GLYMA_06G323600 | 1.01E+08 | protein_coding | Glycine max | 6 | 51.136822 | hypothetical protein |
| GLYMA_06G323700 | 1.01E+08 | protein_coding | Glycine max | 6 | 51.145662 | hypothetical protein |
| GLYMA_06G323800 | NA | protein_coding | Glycine max | 6 | 51.155736 | hypothetical protein |
| GLYMA_06G323900 | 1E+08 | protein_coding | Glycine max | 6 | 51.161239 | hypothetical protein |
| GLYMA_06G324000 | 1.01E+08 | protein_coding | Glycine max | 6 | 51.171477 | hypothetical protein |
| GLYMA_06G324100 | NA | protein_coding | Glycine max | 6 | 51.179208 | hypothetical protein |
| GLYMA_06G324200 | 1.03E+08 | protein_coding | Glycine max | 6 | 51.18216 | hypothetical protein |
| GLYMA_06G324300 | 1.01E+08 | protein_coding | Glycine max | 6 | 51.189321 | hypothetical protein |
| GLYMA_06G324400 | 1E+08 | protein_coding | Glycine max | 6 | 51.205072 | hypothetical protein |
| GLYMA_06G324500 | 1.01E+08 | protein_coding | Glycine max | 6 | 51.224621 | hypothetical protein |
| GLYMA_06G324600 | 1.01E+08 | protein_coding | Glycine max | 6 | 51.247542 | hypothetical protein |
| GLYMA_06G324700 | 1.01E+08 | protein_coding | Glycine max | 6 | 51.26001 | hypothetical protein |
| GLYMA_06G324800 | 1.01E+08 | protein_coding | Glycine max | 6 | 51.264461 | hypothetical protein |
| GLYMA_06G324900 | 1.01E+08 | protein_coding | Glycine max | 6 | 51.277285 | hypothetical protein |
| GLYMA_06G325000 | NA | protein_coding | Glycine max | 6 | 51.282636 | hypothetical protein |
| GLYMA_06G325100 | 1.01E+08 | protein_coding | Glycine max | 6 | 51.283876 | hypothetical protein |
| GLYMA_06G325200 | 1.01E+08 | protein_coding | Glycine max | 6 | 51.293238 | hypothetical protein |
| GLYMA_06G325300 | 1.01E+08 | protein_coding | Glycine max | 6 | 51.303384 | hypothetical protein |
| GLYMA_07G208400 | 1.01E+08 | protein_coding | Glycine max | 7 | 37.80576 | hypothetical protein |
| GLYMA_07G208500 | NA | protein_coding | Glycine max | 7 | 37.818444 | hypothetical protein |
| GLYMA_07G208600 | 1.01E+08 | protein_coding | Glycine max | 7 | 37.825191 | hypothetical protein |
| GLYMA_07G208700 | NA | protein_coding | Glycine max | 7 | 37.858748 | hypothetical protein |
| GLYMA_07G208800 | 1.01E+08 | protein_coding | Glycine max | 7 | 37.862113 | hypothetical protein |
| GLYMA_07G208900 | NA | protein_coding | Glycine max | 7 | 37.874718 | hypothetical protein |
| GLYMA_07G209000 | NA | protein_coding | Glycine max | 7 | 37.878405 | hypothetical protein |
| GLYMA_07G209100 | 1E+08 | protein_coding | Glycine max | 7 | 37.882084 | hypothetical protein |
| GLYMA_07G209200 | NA | protein_coding | Glycine max | 7 | 37.899061 | hypothetical protein |
| GLYMA_07G209300 | 1.01E+08 | protein_coding | Glycine max | 7 | 37.920004 | hypothetical protein |
| GLYMA_07G209400 | NA | protein_coding | Glycine max | 7 | 37.937966 | hypothetical protein |
| GLYMA_07G209500 | 1.01E+08 | protein_coding | Glycine max | 7 | 38.016457 | hypothetical protein |
| GLYMA_07G209600 | NA | protein_coding | Glycine max | 7 | 38.037644 | hypothetical protein |
| GLYMA_07G209700 | NA | protein_coding | Glycine max | 7 | 38.049934 | hypothetical protein |
| GLYMA_07G209800 | NA | protein_coding | Glycine max | 7 | 38.053314 | hypothetical protein |
| GLYMA_07G209900 | 1.01E+08 | protein_coding | Glycine max | 7 | 38.059457 | hypothetical protein |
| GLYMA_07G210000 | 1.01E+08 | protein_coding | Glycine max | 7 | 38.07058 | hypothetical protein |
| GLYMA_07G210100 | NA | protein_coding | Glycine max | 7 | 38.072328 | hypothetical protein |
| GLYMA_07G210200 | 1.01E+08 | protein_coding | Glycine max | 7 | 38.085486 | hypothetical protein |
| GLYMA_07G210300 | NA | protein_coding | Glycine max | 7 | 38.09356 | hypothetical protein |
| GLYMA_07G210400 | NA | protein_coding | Glycine max | 7 | 38.126869 | hypothetical protein |
| GLYMA_07G210500 | NA | protein_coding | Glycine max | 7 | 38.130614 | hypothetical protein |
| GLYMA_07G210600 | 1.01E+08 | protein_coding | Glycine max | 7 | 38.136094 | hypothetical protein |
| GLYMA_07G210700 | 1.01E+08 | protein_coding | Glycine max | 7 | 38.16271 | hypothetical protein |
| GLYMA_07G210800 | 1.01E+08 | protein_coding | Glycine max | 7 | 38.176736 | hypothetical protein |
| GLYMA_07G210900 | 1.01E+08 | protein_coding | Glycine max | 7 | 38.192697 | hypothetical protein |
| GLYMA_07G211000 | 1.01E+08 | protein_coding | Glycine max | 7 | 38.220165 | hypothetical protein |
| GLYMA_07G211100 | 1.01E+08 | protein_coding | Glycine max | 7 | 38.231734 | hypothetical protein |
| GLYMA_07G211200 | NA | protein_coding | Glycine max | 7 | 38.247956 | hypothetical protein |
| GLYMA_07G211300 | 1.01E+08 | protein_coding | Glycine max | 7 | 38.265217 | hypothetical protein |
| GLYMA_07G211400 | 1.01E+08 | protein_coding | Glycine max | 7 | 38.277619 | hypothetical protein |
| GLYMA_07G211500 | NA | protein_coding | Glycine max | 7 | 38.297414 | hypothetical protein |
| GLYMA_08G017100 | NA | protein_coding | Glycine max | 8 | 1.385005 | hypothetical protein |
| GLYMA_08G017200 | 1.01E+08 | protein_coding | Glycine max | 8 | 1.396256 | hypothetical protein |
| GLYMA_08G017300 | 1.01E+08 | protein_coding | Glycine max | 8 | 1.401842 | hypothetical protein |
| GLYMA_08G017400 | 1.01E+08 | protein_coding | Glycine max | 8 | 1.404725 | hypothetical protein |
| GLYMA_08G017500 | NA | protein_coding | Glycine max | 8 | 1.412195 | hypothetical protein |
| GLYMA_08G017600 | NA | protein_coding | Glycine max | 8 | 1.417055 | hypothetical protein |
| GLYMA_08G017700 | 1.01E+08 | protein_coding | Glycine max | 8 | 1.429408 | hypothetical protein |
| GLYMA_08G017800 | 1.01E+08 | protein_coding | Glycine max | 8 | 1.434223 | hypothetical protein |
| GLYMA_08G017900 | 1.01E+08 | protein_coding | Glycine max | 8 | 1.443726 | hypothetical protein |
| GLYMA_08G018000 | NA | protein_coding | Glycine max | 8 | 1.450278 | hypothetical protein |
| GLYMA_08G018100 | NA | protein_coding | Glycine max | 8 | 1.464874 | hypothetical protein |
| GLYMA_08G018200 | NA | protein_coding | Glycine max | 8 | 1.466789 | hypothetical protein |
| GLYMA_08G018300 | 1.03E+08 | protein_coding | Glycine max | 8 | 1.475056 | hypothetical protein |
| GLYMA_08G018400 | NA | protein_coding | Glycine max | 8 | 1.485057 | hypothetical protein |
| GLYMA_08G018600 | NA | protein_coding | Glycine max | 8 | 1.501407 | hypothetical protein |
| GLYMA_08G018700 | 1.01E+08 | protein_coding | Glycine max | 8 | 1.510381 | hypothetical protein |
| GLYMA_08G018800 | NA | protein_coding | Glycine max | 8 | 1.515199 | hypothetical protein |
| GLYMA_08G018900 | 1.01E+08 | protein_coding | Glycine max | 8 | 1.537491 | hypothetical protein |
| GLYMA_08G019000 | 1.01E+08 | protein_coding | Glycine max | 8 | 1.54913 | hypothetical protein |
| GLYMA_08G019100 | NA | protein_coding | Glycine max | 8 | 1.555092 | hypothetical protein |
| GLYMA_08G019200 | NA | protein_coding | Glycine max | 8 | 1.56256 | hypothetical protein |
| GLYMA_08G019300 | 1.01E+08 | protein_coding | Glycine max | 8 | 1.566418 | hypothetical protein |
| GLYMA_08G019400 | 1.01E+08 | protein_coding | Glycine max | 8 | 1.572616 | hypothetical protein |
| GLYMA_08G019500 | NA | protein_coding | Glycine max | 8 | 1.582134 | hypothetical protein |
| GLYMA_08G019600 | 1.01E+08 | protein_coding | Glycine max | 8 | 1.593437 | hypothetical protein |
| GLYMA_08G019700 | NA | protein_coding | Glycine max | 8 | 1.607735 | hypothetical protein |
| GLYMA_08G019800 | 1.01E+08 | protein_coding | Glycine max | 8 | 1.613128 | hypothetical protein |
| GLYMA_08G019900 | NA | protein_coding | Glycine max | 8 | 1.632985 | hypothetical protein |
| GLYMA_08G020000 | 1.01E+08 | protein_coding | Glycine max | 8 | 1.639578 | hypothetical protein |
| GLYMA_08G020100 | NA | protein_coding | Glycine max | 8 | 1.642877 | hypothetical protein |
| GLYMA_08G020200 | NA | protein_coding | Glycine max | 8 | 1.645078 | hypothetical protein |
| GLYMA_08G020300 | 1.01E+08 | protein_coding | Glycine max | 8 | 1.659833 | hypothetical protein |
| GLYMA_08G020400 | 1.01E+08 | protein_coding | Glycine max | 8 | 1.664765 | hypothetical protein |
| GLYMA_08G020500 | 1.01E+08 | protein_coding | Glycine max | 8 | 1.666878 | hypothetical protein |
| GLYMA_08G020600 | 1.07E+08 | protein_coding | Glycine max | 8 | 1.66992 | hypothetical protein |
| GLYMA_08G020700 | NA | protein_coding | Glycine max | 8 | 1.67646 | hypothetical protein |
| GLYMA_08G020800 | 1.01E+08 | protein_coding | Glycine max | 8 | 1.683328 | hypothetical protein |
| GLYMA_08G020900 | 1.01E+08 | protein_coding | Glycine max | 8 | 1.696326 | hypothetical protein |
| GLYMA_08G021000 | NA | protein_coding | Glycine max | 8 | 1.702978 | hypothetical protein |
| GLYMA_08G021100 | NA | protein_coding | Glycine max | 8 | 1.709026 | hypothetical protein |
| GLYMA_08G021200 | 1.01E+08 | protein_coding | Glycine max | 8 | 1.710191 | hypothetical protein |
| GLYMA_08G021300 | NA | protein_coding | Glycine max | 8 | 1.724709 | hypothetical protein |
| GLYMA_08G021400 | 1.01E+08 | protein_coding | Glycine max | 8 | 1.730291 | hypothetical protein |
| GLYMA_08G021500 | 1.01E+08 | protein_coding | Glycine max | 8 | 1.741823 | hypothetical protein |
| GLYMA_08G021600 | 1.01E+08 | protein_coding | Glycine max | 8 | 1.746451 | hypothetical protein |
| GLYMA_08G021700 | 1.01E+08 | protein_coding | Glycine max | 8 | 1.750378 | hypothetical protein |
| GLYMA_08G021800 | 1.01E+08 | protein_coding | Glycine max | 8 | 1.760152 | hypothetical protein |
| GLYMA_08G021900 | NA | protein_coding | Glycine max | 8 | 1.762687 | hypothetical protein |
| GLYMA_08G022000 | 1.01E+08 | protein_coding | Glycine max | 8 | 1.768628 | hypothetical protein |
| GLYMA_08G022100 | NA | protein_coding | Glycine max | 8 | 1.770534 | hypothetical protein |
| GLYMA_08G022200 | 1.03E+08 | protein_coding | Glycine max | 8 | 1.778549 | hypothetical protein |
| GLYMA_08G022300 | NA | protein_coding | Glycine max | 8 | 1.797953 | hypothetical protein |
| GLYMA_08G022400 | NA | protein_coding | Glycine max | 8 | 1.804529 | hypothetical protein |
| GLYMA_08G022500 | NA | protein_coding | Glycine max | 8 | 1.811134 | hypothetical protein |
| GLYMA_08G022600 | NA | protein_coding | Glycine max | 8 | 1.8158 | hypothetical protein |
| GLYMA_08G022700 | NA | protein_coding | Glycine max | 8 | 1.822439 | hypothetical protein |
| GLYMA_08G022800 | 1.03E+08 | protein_coding | Glycine max | 8 | 1.829963 | hypothetical protein |
| GLYMA_08G022900 | 1.03E+08 | protein_coding | Glycine max | 8 | 1.836104 | hypothetical protein |
| GLYMA_08G023000 | NA | protein_coding | Glycine max | 8 | 1.840361 | hypothetical protein |
| GLYMA_08G023100 | 1.01E+08 | protein_coding | Glycine max | 8 | 1.852652 | hypothetical protein |
| GLYMA_08G023200 | NA | protein_coding | Glycine max | 8 | 1.857877 | hypothetical protein |
| GLYMA_08G023300 | 1E+08 | protein_coding | Glycine max | 8 | 1.861918 | hypothetical protein |
| GLYMA_08G023400 | 1.01E+08 | protein_coding | Glycine max | 8 | 1.869318 | hypothetical protein |
| GLYMA_08G023500 | 1.01E+08 | protein_coding | Glycine max | 8 | 1.87367 | hypothetical protein |
| GLYMA_08G023600 | 1.01E+08 | protein_coding | Glycine max | 8 | 1.87725 | hypothetical protein |
| GLYMA_08G043000 | NA | protein_coding | Glycine max | 8 | 3.400493 | hypothetical protein |
| GLYMA_08G043100 | 1.01E+08 | protein_coding | Glycine max | 8 | 3.408821 | hypothetical protein |
| GLYMA_08G043200 | NA | protein_coding | Glycine max | 8 | 3.414089 | hypothetical protein |
| GLYMA_08G043300 | 1E+08 | protein_coding | Glycine max | 8 | 3.416302 | hypothetical protein |
| GLYMA_08G043400 | NA | protein_coding | Glycine max | 8 | 3.420318 | hypothetical protein |
| GLYMA_08G043500 | NA | protein_coding | Glycine max | 8 | 3.426497 | hypothetical protein |
| GLYMA_08G043600 | 1.01E+08 | protein_coding | Glycine max | 8 | 3.43064 | hypothetical protein |
| GLYMA_08G043700 | NA | protein_coding | Glycine max | 8 | 3.438606 | hypothetical protein |
| GLYMA_08G043800 | 1.01E+08 | protein_coding | Glycine max | 8 | 3.450235 | hypothetical protein |
| GLYMA_08G043900 | 1.01E+08 | protein_coding | Glycine max | 8 | 3.458488 | hypothetical protein |
| GLYMA_08G044000 | 1.01E+08 | protein_coding | Glycine max | 8 | 3.474223 | hypothetical protein |
| GLYMA_08G044100 | 1.01E+08 | protein_coding | Glycine max | 8 | 3.486866 | hypothetical protein |
| GLYMA_08G044200 | 1.01E+08 | protein_coding | Glycine max | 8 | 3.500382 | hypothetical protein |
| GLYMA_08G044300 | 1.01E+08 | protein_coding | Glycine max | 8 | 3.507334 | hypothetical protein |
| GLYMA_08G044400 | 1.01E+08 | protein_coding | Glycine max | 8 | 3.511249 | hypothetical protein |
| GLYMA_08G044500 | NA | protein_coding | Glycine max | 8 | 3.517759 | hypothetical protein |
| GLYMA_08G044600 | 1.01E+08 | protein_coding | Glycine max | 8 | 3.526526 | hypothetical protein |
| GLYMA_08G044700 | 1.01E+08 | protein_coding | Glycine max | 8 | 3.539033 | hypothetical protein |
| GLYMA_08G044800 | 1.01E+08 | protein_coding | Glycine max | 8 | 3.554043 | hypothetical protein |
| GLYMA_08G044900 | 1.01E+08 | protein_coding | Glycine max | 8 | 3.559628 | hypothetical protein |
| GLYMA_08G045000 | 1.01E+08 | protein_coding | Glycine max | 8 | 3.567453 | hypothetical protein |
| GLYMA_08G045100 | 1.01E+08 | protein_coding | Glycine max | 8 | 3.575179 | hypothetical protein |
| GLYMA_08G045200 | 1.01E+08 | protein_coding | Glycine max | 8 | 3.581815 | hypothetical protein |
| GLYMA_08G045300 | NA | protein_coding | Glycine max | 8 | 3.58938 | hypothetical protein |
| GLYMA_08G045400 | 1.01E+08 | protein_coding | Glycine max | 8 | 3.590764 | hypothetical protein |
| GLYMA_08G045500 | 1.01E+08 | protein_coding | Glycine max | 8 | 3.594728 | hypothetical protein |
| GLYMA_08G045600 | NA | protein_coding | Glycine max | 8 | 3.595031 | hypothetical protein |
| GLYMA_08G045700 | 1.01E+08 | protein_coding | Glycine max | 8 | 3.603957 | hypothetical protein |
| GLYMA_08G045800 | NA | protein_coding | Glycine max | 8 | 3.608149 | hypothetical protein |
| GLYMA_08G045900 | 1.01E+08 | protein_coding | Glycine max | 8 | 3.619845 | hypothetical protein |
| GLYMA_08G046000 | 1.01E+08 | protein_coding | Glycine max | 8 | 3.630817 | hypothetical protein |
| GLYMA_08G046100 | 1.01E+08 | protein_coding | Glycine max | 8 | 3.636492 | hypothetical protein |
| GLYMA_08G046200 | 1.01E+08 | protein_coding | Glycine max | 8 | 3.640184 | hypothetical protein |
| GLYMA_08G046300 | 1.01E+08 | protein_coding | Glycine max | 8 | 3.646545 | hypothetical protein |
| GLYMA_08G046400 | 1.01E+08 | protein_coding | Glycine max | 8 | 3.648645 | hypothetical protein |
| GLYMA_08G046500 | 1.01E+08 | protein_coding | Glycine max | 8 | 3.658444 | hypothetical protein |
| GLYMA_08G046600 | NA | protein_coding | Glycine max | 8 | 3.663262 | hypothetical protein |
| GLYMA_08G046700 | NA | protein_coding | Glycine max | 8 | 3.667933 | hypothetical protein |
| GLYMA_08G046800 | NA | protein_coding | Glycine max | 8 | 3.671513 | hypothetical protein |
| GLYMA_08G046900 | NA | protein_coding | Glycine max | 8 | 3.676434 | hypothetical protein |
| GLYMA_08G047000 | 1.01E+08 | protein_coding | Glycine max | 8 | 3.678412 | hypothetical protein |
| GLYMA_08G047100 | 1.01E+08 | protein_coding | Glycine max | 8 | 3.68107 | hypothetical protein |
| GLYMA_08G047200 | 1E+08 | protein_coding | Glycine max | 8 | 3.68949 | hypothetical protein |
| GLYMA_08G047300 | 1.01E+08 | protein_coding | Glycine max | 8 | 3.694731 | hypothetical protein |
| GLYMA_08G047400 | NA | protein_coding | Glycine max | 8 | 3.707496 | hypothetical protein |
| GLYMA_08G047500 | 1.01E+08 | protein_coding | Glycine max | 8 | 3.71509 | hypothetical protein |
| GLYMA_08G047600 | NA | protein_coding | Glycine max | 8 | 3.71933 | hypothetical protein |
| GLYMA_08G047700 | NA | protein_coding | Glycine max | 8 | 3.71985 | hypothetical protein |
| GLYMA_08G047800 | 1.01E+08 | protein_coding | Glycine max | 8 | 3.72355 | hypothetical protein |
| GLYMA_08G047900 | 1.01E+08 | protein_coding | Glycine max | 8 | 3.731133 | hypothetical protein |
| GLYMA_08G048000 | 1.01E+08 | protein_coding | Glycine max | 8 | 3.741969 | hypothetical protein |
| GLYMA_08G048100 | 1.01E+08 | protein_coding | Glycine max | 8 | 3.748084 | hypothetical protein |
| GLYMA_08G048200 | 1.01E+08 | protein_coding | Glycine max | 8 | 3.758072 | hypothetical protein |
| GLYMA_08G048300 | NA | protein_coding | Glycine max | 8 | 3.765082 | hypothetical protein |
| GLYMA_08G048400 | 1.01E+08 | protein_coding | Glycine max | 8 | 3.766456 | hypothetical protein |
| GLYMA_08G048500 | NA | protein_coding | Glycine max | 8 | 3.775995 | hypothetical protein |
| GLYMA_08G048600 | NA | protein_coding | Glycine max | 8 | 3.786523 | hypothetical protein |
| GLYMA_08G048700 | 1.01E+08 | protein_coding | Glycine max | 8 | 3.794812 | hypothetical protein |
| GLYMA_08G048800 | 1.01E+08 | protein_coding | Glycine max | 8 | 3.800029 | hypothetical protein |
| GLYMA_08G048900 | 1.03E+08 | protein_coding | Glycine max | 8 | 3.808128 | hypothetical protein |
| GLYMA_08G049000 | 1.01E+08 | protein_coding | Glycine max | 8 | 3.809172 | hypothetical protein |
| GLYMA_08G049100 | NA | protein_coding | Glycine max | 8 | 3.826572 | hypothetical protein |
| GLYMA_08G049200 | 1.01E+08 | protein_coding | Glycine max | 8 | 3.836003 | hypothetical protein |
| GLYMA_08G049300 | 1.01E+08 | protein_coding | Glycine max | 8 | 3.844413 | hypothetical protein |
| GLYMA_08G049400 | 1.01E+08 | protein_coding | Glycine max | 8 | 3.854843 | hypothetical protein |
| GLYMA_08G049500 | NA | protein_coding | Glycine max | 8 | 3.868579 | hypothetical protein |
| GLYMA_08G049600 | NA | protein_coding | Glycine max | 8 | 3.880646 | hypothetical protein |
| GLYMA_08G111900 | 1.01E+08 | protein_coding | Glycine max | 8 | 8.612676 | hypothetical protein |
| GLYMA_08G112000 | 1.01E+08 | protein_coding | Glycine max | 8 | 8.624137 | hypothetical protein |
| GLYMA_08G112100 | 1.01E+08 | protein_coding | Glycine max | 8 | 8.631891 | hypothetical protein |
| GLYMA_08G112200 | NA | protein_coding | Glycine max | 8 | 8.639468 | hypothetical protein |
| GLYMA_08G112300 | 1E+08 | protein_coding | Glycine max | 8 | 8.648286 | hypothetical protein |
| GLYMA_08G112400 | 1.01E+08 | protein_coding | Glycine max | 8 | 8.651985 | hypothetical protein |
| GLYMA_08G112500 | NA | protein_coding | Glycine max | 8 | 8.659541 | hypothetical protein |
| GLYMA_08G112600 | 1.01E+08 | protein_coding | Glycine max | 8 | 8.66067 | hypothetical protein |
| GLYMA_08G112700 | NA | protein_coding | Glycine max | 8 | 8.661027 | hypothetical protein |
| GLYMA_08G112800 | NA | protein_coding | Glycine max | 8 | 8.664809 | hypothetical protein |
| GLYMA_08G112900 | 1.01E+08 | protein_coding | Glycine max | 8 | 8.66574 | hypothetical protein |
| GLYMA_08G113000 | 1.01E+08 | protein_coding | Glycine max | 8 | 8.672927 | hypothetical protein |
| GLYMA_08G113100 | 1.01E+08 | protein_coding | Glycine max | 8 | 8.681998 | hypothetical protein |
| GLYMA_08G113200 | 1.01E+08 | protein_coding | Glycine max | 8 | 8.688329 | hypothetical protein |
| GLYMA_08G113300 | 1.01E+08 | protein_coding | Glycine max | 8 | 8.696443 | hypothetical protein |
| GLYMA_08G113400 | 1.01E+08 | protein_coding | Glycine max | 8 | 8.703339 | hypothetical protein |
| GLYMA_08G113500 | 1.01E+08 | protein_coding | Glycine max | 8 | 8.707827 | hypothetical protein |
| GLYMA_08G113600 | 1.01E+08 | protein_coding | Glycine max | 8 | 8.713767 | hypothetical protein |
| GLYMA_08G113700 | 1.01E+08 | protein_coding | Glycine max | 8 | 8.723285 | hypothetical protein |
| GLYMA_08G113800 | 1.01E+08 | protein_coding | Glycine max | 8 | 8.72836 | hypothetical protein |
| GLYMA_08G113900 | 1.01E+08 | protein_coding | Glycine max | 8 | 8.730526 | hypothetical protein |
| GLYMA_08G114000 | 1.01E+08 | protein_coding | Glycine max | 8 | 8.737686 | hypothetical protein |
| GLYMA_08G114100 | 1.01E+08 | protein_coding | Glycine max | 8 | 8.740815 | hypothetical protein |
| GLYMA_08G114200 | 1.01E+08 | protein_coding | Glycine max | 8 | 8.743328 | hypothetical protein |
| GLYMA_08G114300 | NA | protein_coding | Glycine max | 8 | 8.746643 | hypothetical protein |
| GLYMA_08G114400 | 1.01E+08 | protein_coding | Glycine max | 8 | 8.750828 | hypothetical protein |
| GLYMA_08G114500 | 1.01E+08 | protein_coding | Glycine max | 8 | 8.757033 | hypothetical protein |
| GLYMA_08G114600 | 1.01E+08 | protein_coding | Glycine max | 8 | 8.763388 | hypothetical protein |
| GLYMA_08G114700 | 1.01E+08 | protein_coding | Glycine max | 8 | 8.768854 | hypothetical protein |
| GLYMA_08G114800 | 1.01E+08 | protein_coding | Glycine max | 8 | 8.792663 | hypothetical protein |
| GLYMA_08G114900 | 1.01E+08 | protein_coding | Glycine max | 8 | 8.800031 | hypothetical protein |
| GLYMA_08G115000 | NA | protein_coding | Glycine max | 8 | 8.811074 | hypothetical protein |
| GLYMA_08G115100 | NA | protein_coding | Glycine max | 8 | 8.813513 | hypothetical protein |
| GLYMA_08G115200 | 1.01E+08 | protein_coding | Glycine max | 8 | 8.831228 | hypothetical protein |
| GLYMA_08G115300 | NA | protein_coding | Glycine max | 8 | 8.843785 | hypothetical protein |
| GLYMA_08G115400 | NA | protein_coding | Glycine max | 8 | 8.848959 | hypothetical protein |
| GLYMA_08G115500 | 1.01E+08 | protein_coding | Glycine max | 8 | 8.852265 | hypothetical protein |
| GLYMA_08G115600 | 1.01E+08 | protein_coding | Glycine max | 8 | 8.85886 | hypothetical protein |
| GLYMA_08G115700 | 1.01E+08 | protein_coding | Glycine max | 8 | 8.868075 | hypothetical protein |
| GLYMA_08G115800 | NA | protein_coding | Glycine max | 8 | 8.877809 | hypothetical protein |
| GLYMA_08G115900 | NA | protein_coding | Glycine max | 8 | 8.885582 | hypothetical protein |
| GLYMA_08G116000 | 1.01E+08 | protein_coding | Glycine max | 8 | 8.891285 | hypothetical protein |
| GLYMA_08G116100 | 1.01E+08 | protein_coding | Glycine max | 8 | 8.919428 | hypothetical protein |
| GLYMA_08G116200 | 1.01E+08 | protein_coding | Glycine max | 8 | 8.930267 | hypothetical protein |
| GLYMA_08G116300 | 548062 | protein_coding | Glycine max | 8 | 8.935918 | hypothetical protein |
| GLYMA_08G116400 | NA | protein_coding | Glycine max | 8 | 8.947598 | hypothetical protein |
| GLYMA_08G116500 | 1.01E+08 | protein_coding | Glycine max | 8 | 8.961418 | hypothetical protein |
| GLYMA_08G116600 | 1.01E+08 | protein_coding | Glycine max | 8 | 8.968563 | hypothetical protein |
| GLYMA_08G116700 | 1.01E+08 | protein_coding | Glycine max | 8 | 8.997512 | hypothetical protein |
| GLYMA_08G116800 | 1.03E+08 | protein_coding | Glycine max | 8 | 9.006479 | hypothetical protein |
| GLYMA_08G116900 | 1.01E+08 | protein_coding | Glycine max | 8 | 9.013669 | hypothetical protein |
| GLYMA_08G117000 | 1.01E+08 | protein_coding | Glycine max | 8 | 9.0198 | hypothetical protein |
| GLYMA_08G117100 | NA | protein_coding | Glycine max | 8 | 9.032236 | hypothetical protein |
| GLYMA_08G117200 | 1.01E+08 | protein_coding | Glycine max | 8 | 9.038325 | hypothetical protein |
| GLYMA_08G117300 | 1.01E+08 | protein_coding | Glycine max | 8 | 9.048858 | hypothetical protein |
| GLYMA_08G117400 | NA | protein_coding | Glycine max | 8 | 9.05406 | hypothetical protein |
| GLYMA_08G117500 | NA | protein_coding | Glycine max | 8 | 9.065033 | hypothetical protein |
| GLYMA_08G117600 | NA | protein_coding | Glycine max | 8 | 9.073699 | hypothetical protein |
| GLYMA_08G117700 | NA | protein_coding | Glycine max | 8 | 9.080318 | hypothetical protein |
| GLYMA_08G117800 | NA | protein_coding | Glycine max | 8 | 9.090365 | hypothetical protein |
| GLYMA_08G117900 | NA | protein_coding | Glycine max | 8 | 9.093315 | hypothetical protein |
| GLYMA_08G118000 | 1.01E+08 | protein_coding | Glycine max | 8 | 9.100408 | hypothetical protein |
| GLYMA_09G097100 | NA | protein_coding | Glycine max | 9 | 15.237041 | hypothetical protein |
| GLYMA_09G097200 | 1.01E+08 | protein_coding | Glycine max | 9 | 15.244158 | hypothetical protein |
| GLYMA_09G097300 | 1.01E+08 | protein_coding | Glycine max | 9 | 15.538384 | hypothetical protein |
| GLYMA_09G097400 | NA | protein_coding | Glycine max | 9 | 15.544618 | hypothetical protein |
| GLYMA_09G097500 | 1.01E+08 | protein_coding | Glycine max | 9 | 15.65316 | hypothetical protein |
| GLYMA_09G100400 | NA | protein_coding | Glycine max | 9 | 18.2404 | hypothetical protein |
| GLYMA_09G100500 | 1.03E+08 | protein_coding | Glycine max | 9 | 18.264677 | hypothetical protein |
| GLYMA_09G100600 | NA | protein_coding | Glycine max | 9 | 18.294605 | hypothetical protein |
| GLYMA_09G100700 | NA | protein_coding | Glycine max | 9 | 18.31587 | hypothetical protein |
| GLYMA_09G100800 | NA | protein_coding | Glycine max | 9 | 18.324275 | hypothetical protein |
| GLYMA_09G100900 | NA | protein_coding | Glycine max | 9 | 18.342406 | hypothetical protein |
| GLYMA_09G101000 | 1.07E+08 | protein_coding | Glycine max | 9 | 18.386272 | hypothetical protein |
| GLYMA_09G101100 | NA | protein_coding | Glycine max | 9 | 18.409565 | hypothetical protein |
| GLYMA_09G101200 | 1.01E+08 | protein_coding | Glycine max | 9 | 18.41971 | hypothetical protein |
| GLYMA_09G101300 | 1.03E+08 | protein_coding | Glycine max | 9 | 18.437617 | hypothetical protein |
| GLYMA_09G101400 | NA | protein_coding | Glycine max | 9 | 18.45435 | hypothetical protein |
| GLYMA_09G101500 | NA | protein_coding | Glycine max | 9 | 18.458234 | hypothetical protein |
| GLYMA_09G101600 | NA | protein_coding | Glycine max | 9 | 18.459696 | hypothetical protein |
| GLYMA_09G101700 | NA | protein_coding | Glycine max | 9 | 18.461347 | hypothetical protein |
| GLYMA_09G101800 | NA | protein_coding | Glycine max | 9 | 18.477175 | hypothetical protein |
| GLYMA_09G101900 | NA | protein_coding | Glycine max | 9 | 18.480469 | hypothetical protein |
| GLYMA_09G102000 | NA | protein_coding | Glycine max | 9 | 18.505907 | hypothetical protein |
| GLYMA_09G102100 | 1.01E+08 | protein_coding | Glycine max | 9 | 18.517715 | hypothetical protein |
| GLYMA_09G102200 | NA | protein_coding | Glycine max | 9 | 18.535132 | hypothetical protein |
| GLYMA_09G102300 | 1.03E+08 | protein_coding | Glycine max | 9 | 18.560854 | hypothetical protein |
| GLYMA_09G102400 | NA | protein_coding | Glycine max | 9 | 18.604265 | hypothetical protein |
| GLYMA_09G102500 | NA | protein_coding | Glycine max | 9 | 18.683935 | hypothetical protein |
| GLYMA_09G116000 | NA | protein_coding | Glycine max | 9 | 25.611243 | hypothetical protein |
| GLYMA_09G162400 | NA | protein_coding | Glycine max | 9 | 38.660406 | hypothetical protein |
| GLYMA_09G162500 | 1.01E+08 | protein_coding | Glycine max | 9 | 38.692452 | hypothetical protein |
| GLYMA_09G162600 | NA | protein_coding | Glycine max | 9 | 38.704614 | hypothetical protein |
| GLYMA_09G162700 | 1.01E+08 | protein_coding | Glycine max | 9 | 38.716389 | hypothetical protein |
| GLYMA_09G162800 | 1.01E+08 | protein_coding | Glycine max | 9 | 38.750467 | hypothetical protein |
| GLYMA_09G162900 | NA | protein_coding | Glycine max | 9 | 38.751643 | hypothetical protein |
| GLYMA_09G163000 | 1.01E+08 | protein_coding | Glycine max | 9 | 38.764237 | hypothetical protein |
| GLYMA_09G163100 | 1.01E+08 | protein_coding | Glycine max | 9 | 38.77583 | hypothetical protein |
| GLYMA_09G163200 | 1.01E+08 | protein_coding | Glycine max | 9 | 38.784214 | hypothetical protein |
| GLYMA_09G163300 | 1.01E+08 | protein_coding | Glycine max | 9 | 38.786274 | hypothetical protein |
| GLYMA_09G163400 | NA | protein_coding | Glycine max | 9 | 38.790337 | hypothetical protein |
| GLYMA_09G163500 | NA | protein_coding | Glycine max | 9 | 38.802173 | hypothetical protein |
| GLYMA_09G163600 | 1.01E+08 | protein_coding | Glycine max | 9 | 38.80645 | hypothetical protein |
| GLYMA_09G163700 | 1.01E+08 | protein_coding | Glycine max | 9 | 38.808617 | hypothetical protein |
| GLYMA_09G163800 | 1.01E+08 | protein_coding | Glycine max | 9 | 38.812459 | hypothetical protein |
| GLYMA_09G163900 | 1.01E+08 | protein_coding | Glycine max | 9 | 38.8218 | hypothetical protein |
| GLYMA_09G164000 | NA | protein_coding | Glycine max | 9 | 38.824209 | hypothetical protein |
| GLYMA_09G164100 | NA | protein_coding | Glycine max | 9 | 38.82456 | hypothetical protein |
| GLYMA_09G164200 | 1.01E+08 | protein_coding | Glycine max | 9 | 38.829862 | hypothetical protein |
| GLYMA_09G164300 | 1.01E+08 | protein_coding | Glycine max | 9 | 38.840179 | hypothetical protein |
| GLYMA_09G164400 | NA | protein_coding | Glycine max | 9 | 38.847349 | hypothetical protein |
| GLYMA_09G164500 | 1.01E+08 | protein_coding | Glycine max | 9 | 38.857251 | hypothetical protein |
| GLYMA_09G164600 | 1.01E+08 | protein_coding | Glycine max | 9 | 38.863733 | hypothetical protein |
| GLYMA_09G164700 | 1.01E+08 | protein_coding | Glycine max | 9 | 38.87333 | hypothetical protein |
| GLYMA_09G164800 | NA | protein_coding | Glycine max | 9 | 38.892526 | hypothetical protein |
| GLYMA_09G164900 | 1.01E+08 | protein_coding | Glycine max | 9 | 38.902488 | hypothetical protein |
| GLYMA_09G165000 | 1.01E+08 | protein_coding | Glycine max | 9 | 38.908984 | hypothetical protein |
| GLYMA_09G165100 | 1.01E+08 | protein_coding | Glycine max | 9 | 38.915737 | hypothetical protein |
| GLYMA_09G165200 | 1.01E+08 | protein_coding | Glycine max | 9 | 38.923789 | hypothetical protein |
| GLYMA_09G165300 | 1.01E+08 | protein_coding | Glycine max | 9 | 38.939659 | hypothetical protein |
| GLYMA_09G165400 | 1.01E+08 | protein_coding | Glycine max | 9 | 38.942389 | hypothetical protein |
| GLYMA_09G165500 | NA | protein_coding | Glycine max | 9 | 38.944407 | hypothetical protein |
| GLYMA_09G165600 | 1.01E+08 | protein_coding | Glycine max | 9 | 38.950444 | hypothetical protein |
| GLYMA_09G165700 | 1.01E+08 | protein_coding | Glycine max | 9 | 38.956622 | hypothetical protein |
| GLYMA_09G165800 | NA | protein_coding | Glycine max | 9 | 38.964228 | hypothetical protein |
| GLYMA_09G165900 | 1.01E+08 | protein_coding | Glycine max | 9 | 38.968695 | hypothetical protein |
| GLYMA_09G166000 | NA | protein_coding | Glycine max | 9 | 38.993322 | hypothetical protein |
| GLYMA_09G166100 | 1.01E+08 | protein_coding | Glycine max | 9 | 39.003017 | hypothetical protein |
| GLYMA_09G166200 | 1.01E+08 | protein_coding | Glycine max | 9 | 39.011549 | hypothetical protein |
| GLYMA_09G166300 | 1.01E+08 | protein_coding | Glycine max | 9 | 39.014889 | hypothetical protein |
| GLYMA_09G166400 | 1.01E+08 | protein_coding | Glycine max | 9 | 39.034658 | hypothetical protein |
| GLYMA_09G166500 | 1E+08 | protein_coding | Glycine max | 9 | 39.05047 | hypothetical protein |
| GLYMA_09G166600 | 1.01E+08 | protein_coding | Glycine max | 9 | 39.055156 | hypothetical protein |
| GLYMA_09G166700 | NA | protein_coding | Glycine max | 9 | 39.069813 | hypothetical protein |
| GLYMA_09G166800 | NA | protein_coding | Glycine max | 9 | 39.073166 | hypothetical protein |
| GLYMA_09G166900 | NA | protein_coding | Glycine max | 9 | 39.077338 | hypothetical protein |
| GLYMA_09G167000 | 1.01E+08 | protein_coding | Glycine max | 9 | 39.103764 | hypothetical protein |
| GLYMA_09G167100 | 1.01E+08 | protein_coding | Glycine max | 9 | 39.114301 | hypothetical protein |
| GLYMA_09G167200 | NA | protein_coding | Glycine max | 9 | 39.137136 | hypothetical protein |
| GLYMA_09G167300 | NA | protein_coding | Glycine max | 9 | 39.139395 | hypothetical protein |
| GLYMA_09G167400 | 1.01E+08 | protein_coding | Glycine max | 9 | 39.166685 | hypothetical protein |
| GLYMA_09G167500 | NA | protein_coding | Glycine max | 9 | 39.174197 | hypothetical protein |
| GLYMA_09G167600 | NA | protein_coding | Glycine max | 9 | 39.187078 | hypothetical protein |
| GLYMA_09G167700 | 1.01E+08 | protein_coding | Glycine max | 9 | 39.18814 | hypothetical protein |
| GLYMA_09G167800 | NA | protein_coding | Glycine max | 9 | 39.199969 | hypothetical protein |
| GLYMA_09G167900 | 1E+08 | protein_coding | Glycine max | 9 | 39.203315 | hypothetical protein |
| GLYMA_09G168000 | 1.01E+08 | protein_coding | Glycine max | 9 | 39.235917 | hypothetical protein |
| GLYMA_09G168100 | NA | protein_coding | Glycine max | 9 | 39.248017 | hypothetical protein |
| GLYMA_09G168200 | NA | protein_coding | Glycine max | 9 | 39.287034 | hypothetical protein |
| GLYMA_09G168300 | 1.01E+08 | protein_coding | Glycine max | 9 | 39.28929 | hypothetical protein |
| GLYMA_09G168400 | NA | protein_coding | Glycine max | 9 | 39.295753 | hypothetical protein |
| GLYMA_09G168500 | NA | protein_coding | Glycine max | 9 | 39.311579 | hypothetical protein |
| GLYMA_09G168600 | 1.01E+08 | protein_coding | Glycine max | 9 | 39.328214 | hypothetical protein |
| GLYMA_09G168700 | 1.01E+08 | protein_coding | Glycine max | 9 | 39.338715 | hypothetical protein |
| GLYMA_09G168800 | NA | protein_coding | Glycine max | 9 | 39.356627 | hypothetical protein |
| GLYMA_09G168900 | 1.01E+08 | protein_coding | Glycine max | 9 | 39.359163 | hypothetical protein |
| GLYMA_09G169000 | NA | protein_coding | Glycine max | 9 | 39.364678 | hypothetical protein |
| GLYMA_09G169100 | NA | protein_coding | Glycine max | 9 | 39.370346 | hypothetical protein |
| GLYMA_09G169200 | NA | protein_coding | Glycine max | 9 | 39.374789 | hypothetical protein |
| GLYMA_09G169300 | 1.01E+08 | protein_coding | Glycine max | 9 | 39.386161 | hypothetical protein |
| GLYMA_09G169400 | NA | protein_coding | Glycine max | 9 | 39.398732 | hypothetical protein |
| GLYMA_09G169500 | 1.01E+08 | protein_coding | Glycine max | 9 | 39.409999 | hypothetical protein |
| GLYMA_09G169600 | 1.01E+08 | protein_coding | Glycine max | 9 | 39.420294 | hypothetical protein |
| GLYMA_09G169700 | NA | protein_coding | Glycine max | 9 | 39.437065 | hypothetical protein |
| GLYMA_09G169800 | NA | protein_coding | Glycine max | 9 | 39.439452 | hypothetical protein |
| GLYMA_09G169900 | NA | protein_coding | Glycine max | 9 | 39.441495 | hypothetical protein |
| GLYMA_09G170000 | 1.01E+08 | protein_coding | Glycine max | 9 | 39.453516 | hypothetical protein |
| GLYMA_09G170100 | 1.03E+08 | protein_coding | Glycine max | 9 | 39.462236 | hypothetical protein |
| GLYMA_09G170200 | 1.01E+08 | protein_coding | Glycine max | 9 | 39.503742 | hypothetical protein |
| GLYMA_09G170300 | 1.01E+08 | protein_coding | Glycine max | 9 | 39.52512 | hypothetical protein |
| GLYMA_09G170400 | 606296 | protein_coding | Glycine max | 9 | 39.525194 | hypothetical protein |
| GLYMA_09G170500 | NA | protein_coding | Glycine max | 9 | 39.547233 | hypothetical protein |
| GLYMA_09G170600 | 1.01E+08 | protein_coding | Glycine max | 9 | 39.553322 | hypothetical protein |
| GLYMA_09G170700 | NA | protein_coding | Glycine max | 9 | 39.562228 | hypothetical protein |
| GLYMA_09G170800 | NA | protein_coding | Glycine max | 9 | 39.573543 | hypothetical protein |
| GLYMA_09G170900 | 1.03E+08 | protein_coding | Glycine max | 9 | 39.577103 | hypothetical protein |
| GLYMA_09G171000 | 1.01E+08 | protein_coding | Glycine max | 9 | 39.588317 | hypothetical protein |
| GLYMA_09G171100 | 1.01E+08 | protein_coding | Glycine max | 9 | 39.607686 | hypothetical protein |
| GLYMA_09G171200 | NA | protein_coding | Glycine max | 9 | 39.623177 | hypothetical protein |
| GLYMA_09G171400 | NA | protein_coding | Glycine max | 9 | 39.627087 | hypothetical protein |
| GLYMA_09G171500 | NA | protein_coding | Glycine max | 9 | 39.630511 | hypothetical protein |
| GLYMA_09G171600 | NA | protein_coding | Glycine max | 9 | 39.644737 | hypothetical protein |
| GLYMA_09G171700 | NA | protein_coding | Glycine max | 9 | 39.651499 | hypothetical protein |
| GLYMA_09G171800 | NA | protein_coding | Glycine max | 9 | 39.653896 | hypothetical protein |
| GLYMA_09G171900 | 1.01E+08 | protein_coding | Glycine max | 9 | 39.656849 | hypothetical protein |
| GLYMA_09G172000 | 1.01E+08 | protein_coding | Glycine max | 9 | 39.664672 | hypothetical protein |
| GLYMA_10G062700 | 1.01E+08 | protein_coding | Glycine max | 10 | 5.90331 | hypothetical protein |
| GLYMA_10G062800 | NA | protein_coding | Glycine max | 10 | 5.932914 | hypothetical protein |
| GLYMA_10G062900 | 1.01E+08 | protein_coding | Glycine max | 10 | 5.944827 | hypothetical protein |
| GLYMA_10G063000 | 1.01E+08 | protein_coding | Glycine max | 10 | 5.954818 | hypothetical protein |
| GLYMA_10G063100 | NA | protein_coding | Glycine max | 10 | 5.977612 | hypothetical protein |
| GLYMA_10G063200 | 1.01E+08 | protein_coding | Glycine max | 10 | 5.987307 | hypothetical protein |
| GLYMA_10G063300 | NA | protein_coding | Glycine max | 10 | 5.991476 | hypothetical protein |
| GLYMA_10G063400 | 1.01E+08 | protein_coding | Glycine max | 10 | 6.000293 | hypothetical protein |
| GLYMA_10G063500 | NA | protein_coding | Glycine max | 10 | 6.003921 | hypothetical protein |
| GLYMA_10G063600 | NA | protein_coding | Glycine max | 10 | 6.009368 | hypothetical protein |
| GLYMA_10G063700 | 1.01E+08 | protein_coding | Glycine max | 10 | 6.02729 | hypothetical protein |
| GLYMA_10G063800 | NA | protein_coding | Glycine max | 10 | 6.031694 | hypothetical protein |
| GLYMA_10G063900 | NA | protein_coding | Glycine max | 10 | 6.037856 | hypothetical protein |
| GLYMA_10G064000 | 1.01E+08 | protein_coding | Glycine max | 10 | 6.063426 | hypothetical protein |
| GLYMA_10G064100 | NA | protein_coding | Glycine max | 10 | 6.070406 | hypothetical protein |
| GLYMA_10G064200 | 1.01E+08 | protein_coding | Glycine max | 10 | 6.077042 | hypothetical protein |
| GLYMA_10G064300 | NA | protein_coding | Glycine max | 10 | 6.110216 | hypothetical protein |
| GLYMA_10G064400 | 1.01E+08 | protein_coding | Glycine max | 10 | 6.115939 | hypothetical protein |
| GLYMA_10G064500 | 1.01E+08 | protein_coding | Glycine max | 10 | 6.140338 | hypothetical protein |
| GLYMA_10G064600 | NA | protein_coding | Glycine max | 10 | 6.146718 | hypothetical protein |
| GLYMA_10G064700 | 1.01E+08 | protein_coding | Glycine max | 10 | 6.157225 | hypothetical protein |
| GLYMA_10G064800 | 1.01E+08 | protein_coding | Glycine max | 10 | 6.178462 | hypothetical protein |
| GLYMA_10G064900 | NA | protein_coding | Glycine max | 10 | 6.204815 | hypothetical protein |
| GLYMA_10G065000 | 1.01E+08 | protein_coding | Glycine max | 10 | 6.209769 | hypothetical protein |
| GLYMA_10G065100 | 1.01E+08 | protein_coding | Glycine max | 10 | 6.219832 | hypothetical protein |
| GLYMA_10G065200 | 1.01E+08 | protein_coding | Glycine max | 10 | 6.225456 | hypothetical protein |
| GLYMA_10G065300 | 1.01E+08 | protein_coding | Glycine max | 10 | 6.236296 | hypothetical protein |
| GLYMA_10G065400 | 1.01E+08 | protein_coding | Glycine max | 10 | 6.242662 | hypothetical protein |
| GLYMA_10G065500 | 1.01E+08 | protein_coding | Glycine max | 10 | 6.273279 | hypothetical protein |
| GLYMA_10G065600 | 1.01E+08 | protein_coding | Glycine max | 10 | 6.289213 | hypothetical protein |
| GLYMA_10G065800 | 1.01E+08 | protein_coding | Glycine max | 10 | 6.348198 | hypothetical protein |
| GLYMA_10G065900 | 1.01E+08 | protein_coding | Glycine max | 10 | 6.363826 | hypothetical protein |
| GLYMA_10G066000 | 1.01E+08 | protein_coding | Glycine max | 10 | 6.387821 | hypothetical protein |
| GLYMA_10G066100 | 1.01E+08 | protein_coding | Glycine max | 10 | 6.399365 | hypothetical protein |
| GLYMA_10G066200 | 1.01E+08 | protein_coding | Glycine max | 10 | 6.403969 | hypothetical protein |
| GLYMA_10G066300 | 1.01E+08 | protein_coding | Glycine max | 10 | 6.420465 | hypothetical protein |
| GLYMA_10G066400 | NA | protein_coding | Glycine max | 10 | 6.429348 | hypothetical protein |
| GLYMA_10G066500 | NA | protein_coding | Glycine max | 10 | 6.458374 | hypothetical protein |
| GLYMA_10G066600 | 1.01E+08 | protein_coding | Glycine max | 10 | 6.461628 | hypothetical protein |
| GLYMA_10G066700 | NA | protein_coding | Glycine max | 10 | 6.46991 | hypothetical protein |
| GLYMA_10G066800 | 1.01E+08 | protein_coding | Glycine max | 10 | 6.494688 | hypothetical protein |
| GLYMA_10G066900 | 1.01E+08 | protein_coding | Glycine max | 10 | 6.536855 | hypothetical protein |
| GLYMA_10G067000 | 1.01E+08 | protein_coding | Glycine max | 10 | 6.572361 | hypothetical protein |
| GLYMA_10G067100 | 1.01E+08 | protein_coding | Glycine max | 10 | 6.584854 | hypothetical protein |
| GLYMA_10G067200 | 1.01E+08 | protein_coding | Glycine max | 10 | 6.610956 | hypothetical protein |
| GLYMA_10G067300 | NA | protein_coding | Glycine max | 10 | 6.616254 | hypothetical protein |
| GLYMA_10G067400 | 1.01E+08 | protein_coding | Glycine max | 10 | 6.620521 | hypothetical protein |
| GLYMA_10G067500 | NA | protein_coding | Glycine max | 10 | 6.647053 | hypothetical protein |
| GLYMA_10G067600 | NA | protein_coding | Glycine max | 10 | 6.652099 | hypothetical protein |
| GLYMA_10G067700 | NA | protein_coding | Glycine max | 10 | 6.658921 | hypothetical protein |
| GLYMA_10G067800 | 1.01E+08 | protein_coding | Glycine max | 10 | 6.668829 | hypothetical protein |
| GLYMA_10G067900 | NA | protein_coding | Glycine max | 10 | 6.697335 | hypothetical protein |
| GLYMA_10G068000 | NA | protein_coding | Glycine max | 10 | 6.705094 | hypothetical protein |
| GLYMA_10G068100 | 1.01E+08 | protein_coding | Glycine max | 10 | 6.714929 | hypothetical protein |
| GLYMA_10G228000 | NA | protein_coding | Glycine max | 10 | 45.840732 | hypothetical protein |
| GLYMA_10G228100 | 1.03E+08 | protein_coding | Glycine max | 10 | 45.85463 | hypothetical protein |
| GLYMA_10G228200 | NA | protein_coding | Glycine max | 10 | 45.862564 | hypothetical protein |
| GLYMA_10G228300 | 1.01E+08 | protein_coding | Glycine max | 10 | 45.873599 | hypothetical protein |
| GLYMA_10G228400 | NA | protein_coding | Glycine max | 10 | 45.874708 | hypothetical protein |
| GLYMA_10G228500 | NA | protein_coding | Glycine max | 10 | 45.87695 | hypothetical protein |
| GLYMA_10G228600 | 1.01E+08 | protein_coding | Glycine max | 10 | 45.880533 | hypothetical protein |
| GLYMA_10G228700 | 1.01E+08 | protein_coding | Glycine max | 10 | 45.886382 | hypothetical protein |
| GLYMA_10G228800 | NA | protein_coding | Glycine max | 10 | 45.893094 | hypothetical protein |
| GLYMA_10G228900 | 1.01E+08 | protein_coding | Glycine max | 10 | 45.897641 | hypothetical protein |
| GLYMA_10G229000 | 1.01E+08 | protein_coding | Glycine max | 10 | 45.906187 | hypothetical protein |
| GLYMA_10G229100 | NA | protein_coding | Glycine max | 10 | 45.911687 | hypothetical protein |
| GLYMA_10G229200 | NA | protein_coding | Glycine max | 10 | 45.914342 | hypothetical protein |
| GLYMA_10G229300 | 1.01E+08 | protein_coding | Glycine max | 10 | 45.923278 | hypothetical protein |
| GLYMA_10G229400 | NA | protein_coding | Glycine max | 10 | 45.951812 | hypothetical protein |
| GLYMA_10G229500 | NA | protein_coding | Glycine max | 10 | 45.961076 | hypothetical protein |
| GLYMA_10G229600 | 1.01E+08 | protein_coding | Glycine max | 10 | 45.968793 | hypothetical protein |
| GLYMA_10G229700 | 1.03E+08 | protein_coding | Glycine max | 10 | 45.984999 | hypothetical protein |
| GLYMA_10G229800 | NA | protein_coding | Glycine max | 10 | 45.996215 | hypothetical protein |
| GLYMA_10G229900 | 1.01E+08 | protein_coding | Glycine max | 10 | 46.001071 | hypothetical protein |
| GLYMA_10G230000 | NA | protein_coding | Glycine max | 10 | 46.013366 | hypothetical protein |
| GLYMA_10G230100 | 1.01E+08 | protein_coding | Glycine max | 10 | 46.018669 | hypothetical protein |
| GLYMA_10G230200 | 1E+08 | protein_coding | Glycine max | 10 | 46.024155 | hypothetical protein |
| GLYMA_10G230300 | NA | protein_coding | Glycine max | 10 | 46.036884 | hypothetical protein |
| GLYMA_10G230400 | 1.01E+08 | protein_coding | Glycine max | 10 | 46.039692 | hypothetical protein |
| GLYMA_10G230500 | 1.01E+08 | protein_coding | Glycine max | 10 | 46.044438 | hypothetical protein |
| GLYMA_10G230600 | 1E+08 | protein_coding | Glycine max | 10 | 46.047209 | hypothetical protein |
| GLYMA_10G230700 | 1.01E+08 | protein_coding | Glycine max | 10 | 46.060022 | hypothetical protein |
| GLYMA_10G230800 | NA | protein_coding | Glycine max | 10 | 46.065779 | hypothetical protein |
| GLYMA_10G230900 | 1.01E+08 | protein_coding | Glycine max | 10 | 46.072235 | hypothetical protein |
| GLYMA_10G231000 | NA | protein_coding | Glycine max | 10 | 46.077416 | hypothetical protein |
| GLYMA_10G231100 | NA | protein_coding | Glycine max | 10 | 46.08515 | hypothetical protein |
| GLYMA_10G231200 | 547758 | protein_coding | Glycine max | 10 | 46.093079 | hypothetical protein |
| GLYMA_10G231300 | NA | protein_coding | Glycine max | 10 | 46.101504 | hypothetical protein |
| GLYMA_10G231400 | 1.01E+08 | protein_coding | Glycine max | 10 | 46.106023 | hypothetical protein |
| GLYMA_10G231500 | 1.01E+08 | protein_coding | Glycine max | 10 | 46.111104 | hypothetical protein |
| GLYMA_10G231600 | NA | protein_coding | Glycine max | 10 | 46.121774 | hypothetical protein |
| GLYMA_10G231700 | 1.01E+08 | protein_coding | Glycine max | 10 | 46.133598 | hypothetical protein |
| GLYMA_10G231800 | NA | protein_coding | Glycine max | 10 | 46.138896 | hypothetical protein |
| GLYMA_10G231900 | 1.01E+08 | protein_coding | Glycine max | 10 | 46.142748 | hypothetical protein |
| GLYMA_10G232000 | 1.01E+08 | protein_coding | Glycine max | 10 | 46.154299 | hypothetical protein |
| GLYMA_10G232100 | NA | protein_coding | Glycine max | 10 | 46.167448 | hypothetical protein |
| GLYMA_10G232200 | 1.01E+08 | protein_coding | Glycine max | 10 | 46.17028 | hypothetical protein |
| GLYMA_10G232300 | 1.01E+08 | protein_coding | Glycine max | 10 | 46.18012 | hypothetical protein |
| GLYMA_10G232400 | 1.01E+08 | protein_coding | Glycine max | 10 | 46.187567 | hypothetical protein |
| GLYMA_10G232500 | 1.01E+08 | protein_coding | Glycine max | 10 | 46.200481 | hypothetical protein |
| GLYMA_10G232600 | 1.01E+08 | protein_coding | Glycine max | 10 | 46.208026 | hypothetical protein |
| GLYMA_10G232700 | 1.01E+08 | protein_coding | Glycine max | 10 | 46.217284 | hypothetical protein |
| GLYMA_10G232800 | 1.01E+08 | protein_coding | Glycine max | 10 | 46.221921 | hypothetical protein |
| GLYMA_10G232900 | NA | protein_coding | Glycine max | 10 | 46.231714 | hypothetical protein |
| GLYMA_10G233000 | NA | protein_coding | Glycine max | 10 | 46.233655 | hypothetical protein |
| GLYMA_10G233100 | 1.01E+08 | protein_coding | Glycine max | 10 | 46.237132 | hypothetical protein |
| GLYMA_10G233200 | 1.01E+08 | protein_coding | Glycine max | 10 | 46.244187 | hypothetical protein |
| GLYMA_10G233300 | 1.01E+08 | protein_coding | Glycine max | 10 | 46.253499 | hypothetical protein |
| GLYMA_10G233400 | 1.01E+08 | protein_coding | Glycine max | 10 | 46.256167 | hypothetical protein |
| GLYMA_10G233500 | 1.01E+08 | protein_coding | Glycine max | 10 | 46.266298 | hypothetical protein |
| GLYMA_10G233600 | NA | protein_coding | Glycine max | 10 | 46.281387 | hypothetical protein |
| GLYMA_10G233700 | 1.01E+08 | protein_coding | Glycine max | 10 | 46.285252 | hypothetical protein |
| GLYMA_10G233800 | NA | protein_coding | Glycine max | 10 | 46.291715 | hypothetical protein |
| GLYMA_10G233900 | 1.01E+08 | protein_coding | Glycine max | 10 | 46.295074 | hypothetical protein |
| GLYMA_10G234000 | 1.01E+08 | protein_coding | Glycine max | 10 | 46.296828 | hypothetical protein |
| GLYMA_10G234100 | 1.01E+08 | protein_coding | Glycine max | 10 | 46.304593 | hypothetical protein |
| GLYMA_10G234200 | 1.01E+08 | protein_coding | Glycine max | 10 | 46.331672 | hypothetical protein |
| GLYMA_10G234300 | NA | protein_coding | Glycine max | 10 | 46.335347 | hypothetical protein |
| GLYMA_11G140800 | 1.01E+08 | protein_coding | Glycine max | 11 | 10.751702 | hypothetical protein |
| GLYMA_11G140900 | 1E+08 | protein_coding | Glycine max | 11 | 10.763326 | hypothetical protein |
| GLYMA_11G141000 | 1.01E+08 | protein_coding | Glycine max | 11 | 10.770348 | hypothetical protein |
| GLYMA_11G141100 | 1.01E+08 | protein_coding | Glycine max | 11 | 10.775216 | hypothetical protein |
| GLYMA_11G141200 | NA | protein_coding | Glycine max | 11 | 10.78363 | hypothetical protein |
| GLYMA_11G141300 | 1.01E+08 | protein_coding | Glycine max | 11 | 10.790718 | hypothetical protein |
| GLYMA_11G141400 | NA | protein_coding | Glycine max | 11 | 10.79659 | hypothetical protein |
| GLYMA_11G141500 | 1.01E+08 | protein_coding | Glycine max | 11 | 10.802687 | hypothetical protein |
| GLYMA_11G141600 | 1E+08 | protein_coding | Glycine max | 11 | 10.815466 | hypothetical protein |
| GLYMA_11G141700 | 1E+08 | protein_coding | Glycine max | 11 | 10.819724 | hypothetical protein |
| GLYMA_11G141800 | 1.01E+08 | protein_coding | Glycine max | 11 | 10.825602 | hypothetical protein |
| GLYMA_11G141900 | NA | protein_coding | Glycine max | 11 | 10.83209 | hypothetical protein |
| GLYMA_11G142000 | NA | protein_coding | Glycine max | 11 | 10.833308 | hypothetical protein |
| GLYMA_11G142100 | 1E+08 | protein_coding | Glycine max | 11 | 10.835513 | hypothetical protein |
| GLYMA_11G142200 | NA | protein_coding | Glycine max | 11 | 10.841923 | hypothetical protein |
| GLYMA_11G142300 | 1.01E+08 | protein_coding | Glycine max | 11 | 10.855872 | hypothetical protein |
| GLYMA_11G142400 | 1.01E+08 | protein_coding | Glycine max | 11 | 10.860992 | hypothetical protein |
| GLYMA_11G142500 | NA | protein_coding | Glycine max | 11 | 10.865597 | hypothetical protein |
| GLYMA_11G142600 | 1.01E+08 | protein_coding | Glycine max | 11 | 10.872527 | hypothetical protein |
| GLYMA_11G142700 | 1.01E+08 | protein_coding | Glycine max | 11 | 10.889488 | hypothetical protein |
| GLYMA_11G142800 | NA | protein_coding | Glycine max | 11 | 10.909742 | hypothetical protein |
| GLYMA_11G142900 | NA | protein_coding | Glycine max | 11 | 10.915815 | hypothetical protein |
| GLYMA_11G143000 | 1.01E+08 | protein_coding | Glycine max | 11 | 10.919661 | hypothetical protein |
| GLYMA_11G143100 | NA | protein_coding | Glycine max | 11 | 10.924084 | hypothetical protein |
| GLYMA_11G143200 | 1.01E+08 | protein_coding | Glycine max | 11 | 10.929223 | hypothetical protein |
| GLYMA_11G143300 | 1.01E+08 | protein_coding | Glycine max | 11 | 10.947238 | hypothetical protein |
| GLYMA_11G143400 | 1.01E+08 | protein_coding | Glycine max | 11 | 10.950967 | hypothetical protein |
| GLYMA_11G143500 | NA | protein_coding | Glycine max | 11 | 10.953838 | hypothetical protein |
| GLYMA_11G143600 | NA | protein_coding | Glycine max | 11 | 10.96609 | hypothetical protein |
| GLYMA_11G143700 | 1.03E+08 | protein_coding | Glycine max | 11 | 10.972434 | hypothetical protein |
| GLYMA_11G143800 | 1.01E+08 | protein_coding | Glycine max | 11 | 10.973787 | hypothetical protein |
| GLYMA_11G144000 | 1.01E+08 | protein_coding | Glycine max | 11 | 10.984327 | hypothetical protein |
| GLYMA_11G144100 | NA | protein_coding | Glycine max | 11 | 10.994674 | hypothetical protein |
| GLYMA_11G144200 | NA | protein_coding | Glycine max | 11 | 11.00062 | hypothetical protein |
| GLYMA_11G144300 | 1.01E+08 | protein_coding | Glycine max | 11 | 11.00382 | hypothetical protein |
| GLYMA_11G144400 | NA | protein_coding | Glycine max | 11 | 11.017331 | hypothetical protein |
| GLYMA_11G144500 | 1.01E+08 | protein_coding | Glycine max | 11 | 11.040836 | hypothetical protein |
| GLYMA_11G144600 | 1.01E+08 | protein_coding | Glycine max | 11 | 11.057446 | hypothetical protein |
| GLYMA_11G144700 | NA | protein_coding | Glycine max | 11 | 11.09039 | hypothetical protein |
| GLYMA_11G144800 | 1E+08 | protein_coding | Glycine max | 11 | 11.09695 | hypothetical protein |
| GLYMA_11G144900 | 1.01E+08 | protein_coding | Glycine max | 11 | 11.111175 | hypothetical protein |
| GLYMA_11G145000 | 1.01E+08 | protein_coding | Glycine max | 11 | 11.11776 | hypothetical protein |
| GLYMA_11G145100 | 1.01E+08 | protein_coding | Glycine max | 11 | 11.144477 | hypothetical protein |
| GLYMA_11G145200 | 1.01E+08 | protein_coding | Glycine max | 11 | 11.158223 | hypothetical protein |
| GLYMA_11G145400 | 1.01E+08 | protein_coding | Glycine max | 11 | 11.173794 | hypothetical protein |
| GLYMA_11G145500 | 1.01E+08 | protein_coding | Glycine max | 11 | 11.195651 | hypothetical protein |
| GLYMA_11G145600 | NA | protein_coding | Glycine max | 11 | 11.20488 | hypothetical protein |
| GLYMA_11G145700 | 1.01E+08 | protein_coding | Glycine max | 11 | 11.222052 | hypothetical protein |
| GLYMA_11G145800 | 1.01E+08 | protein_coding | Glycine max | 11 | 11.229131 | hypothetical protein |
| GLYMA_11G230100 | NA | protein_coding | Glycine max | 11 | 32.53625 | hypothetical protein |
| GLYMA_11G230200 | NA | protein_coding | Glycine max | 11 | 32.542067 | hypothetical protein |
| GLYMA_11G230300 | NA | protein_coding | Glycine max | 11 | 32.550584 | hypothetical protein |
| GLYMA_11G230400 | 1.01E+08 | protein_coding | Glycine max | 11 | 32.560337 | hypothetical protein |
| GLYMA_11G230500 | 1.01E+08 | protein_coding | Glycine max | 11 | 32.578091 | hypothetical protein |
| GLYMA_11G230600 | 1.01E+08 | protein_coding | Glycine max | 11 | 32.585898 | hypothetical protein |
| GLYMA_11G230700 | 1.01E+08 | protein_coding | Glycine max | 11 | 32.618499 | hypothetical protein |
| GLYMA_11G230800 | 1.01E+08 | protein_coding | Glycine max | 11 | 32.632906 | hypothetical protein |
| GLYMA_11G230900 | 1.01E+08 | protein_coding | Glycine max | 11 | 32.657915 | hypothetical protein |
| GLYMA_11G231000 | 1E+08 | protein_coding | Glycine max | 11 | 32.666235 | hypothetical protein |
| GLYMA_11G231100 | 1.01E+08 | protein_coding | Glycine max | 11 | 32.671043 | hypothetical protein |
| GLYMA_11G231200 | 1.01E+08 | protein_coding | Glycine max | 11 | 32.684103 | hypothetical protein |
| GLYMA_11G231300 | NA | protein_coding | Glycine max | 11 | 32.690044 | hypothetical protein |
| GLYMA_11G231400 | 1.01E+08 | protein_coding | Glycine max | 11 | 32.701815 | hypothetical protein |
| GLYMA_11G231500 | 1E+08 | protein_coding | Glycine max | 11 | 32.716751 | hypothetical protein |
| GLYMA_11G231600 | 1.01E+08 | protein_coding | Glycine max | 11 | 32.72684 | hypothetical protein |
| GLYMA_11G231700 | 1.01E+08 | protein_coding | Glycine max | 11 | 32.73434 | hypothetical protein |
| GLYMA_11G231800 | 1.01E+08 | protein_coding | Glycine max | 11 | 32.74862 | hypothetical protein |
| GLYMA_11G231900 | NA | protein_coding | Glycine max | 11 | 32.750522 | hypothetical protein |
| GLYMA_11G232000 | 1.01E+08 | protein_coding | Glycine max | 11 | 32.75482 | hypothetical protein |
| GLYMA_11G232100 | 1.01E+08 | protein_coding | Glycine max | 11 | 32.757585 | hypothetical protein |
| GLYMA_11G232200 | 1.01E+08 | protein_coding | Glycine max | 11 | 32.761439 | hypothetical protein |
| GLYMA_11G232300 | 1.01E+08 | protein_coding | Glycine max | 11 | 32.775891 | hypothetical protein |
| GLYMA_11G232400 | 1.01E+08 | protein_coding | Glycine max | 11 | 32.780782 | hypothetical protein |
| GLYMA_11G232500 | 1.01E+08 | protein_coding | Glycine max | 11 | 32.786193 | hypothetical protein |
| GLYMA_11G232600 | 547476 | protein_coding | Glycine max | 11 | 32.791011 | hypothetical protein |
| GLYMA_11G232700 | 1.01E+08 | protein_coding | Glycine max | 11 | 32.811723 | hypothetical protein |
| GLYMA_11G232800 | NA | protein_coding | Glycine max | 11 | 32.812928 | hypothetical protein |
| GLYMA_11G232900 | 1.01E+08 | protein_coding | Glycine max | 11 | 32.82443 | hypothetical protein |
| GLYMA_11G233000 | NA | protein_coding | Glycine max | 11 | 32.831791 | hypothetical protein |
| GLYMA_11G233100 | 1.01E+08 | protein_coding | Glycine max | 11 | 32.840573 | hypothetical protein |
| GLYMA_11G233200 | NA | protein_coding | Glycine max | 11 | 32.846247 | hypothetical protein |
| GLYMA_11G233300 | 1.01E+08 | protein_coding | Glycine max | 11 | 32.849168 | hypothetical protein |
| GLYMA_11G233400 | 1.01E+08 | protein_coding | Glycine max | 11 | 32.873226 | hypothetical protein |
| GLYMA_11G233500 | 1.01E+08 | protein_coding | Glycine max | 11 | 32.884338 | hypothetical protein |
| GLYMA_11G233600 | NA | protein_coding | Glycine max | 11 | 32.893576 | hypothetical protein |
| GLYMA_11G233700 | 1.01E+08 | protein_coding | Glycine max | 11 | 32.902842 | hypothetical protein |
| GLYMA_11G233800 | 1.01E+08 | protein_coding | Glycine max | 11 | 32.914382 | hypothetical protein |
| GLYMA_11G233900 | 1.01E+08 | protein_coding | Glycine max | 11 | 32.921203 | hypothetical protein |
| GLYMA_11G234000 | 1.01E+08 | protein_coding | Glycine max | 11 | 32.930219 | hypothetical protein |
| GLYMA_11G234100 | 1.01E+08 | protein_coding | Glycine max | 11 | 32.934453 | hypothetical protein |
| GLYMA_11G234200 | 1.01E+08 | protein_coding | Glycine max | 11 | 32.937569 | hypothetical protein |
| GLYMA_11G234300 | NA | protein_coding | Glycine max | 11 | 32.957014 | hypothetical protein |
| GLYMA_11G234400 | 1.01E+08 | protein_coding | Glycine max | 11 | 32.964027 | hypothetical protein |
| GLYMA_11G234500 | NA | protein_coding | Glycine max | 11 | 32.967943 | hypothetical protein |
| GLYMA_11G234600 | 1.01E+08 | protein_coding | Glycine max | 11 | 32.979921 | hypothetical protein |
| GLYMA_11G234700 | 1.01E+08 | protein_coding | Glycine max | 11 | 32.984275 | hypothetical protein |
| GLYMA_11G234800 | 1.01E+08 | protein_coding | Glycine max | 11 | 32.993579 | hypothetical protein |
| GLYMA_11G234900 | 1.01E+08 | protein_coding | Glycine max | 11 | 32.998464 | hypothetical protein |
| GLYMA_11G235000 | 1.01E+08 | protein_coding | Glycine max | 11 | 33.004038 | hypothetical protein |
| GLYMA_11G235100 | NA | protein_coding | Glycine max | 11 | 33.013266 | hypothetical protein |
| GLYMA_11G235200 | 1.01E+08 | protein_coding | Glycine max | 11 | 33.022628 | hypothetical protein |
| GLYMA_11G235300 | 1.01E+08 | protein_coding | Glycine max | 11 | 33.02924 | hypothetical protein |
| GLYMA_12G011200 | 1.01E+08 | protein_coding | Glycine max | 12 | 0.822453 | hypothetical protein |
| GLYMA_12G011300 | NA | protein_coding | Glycine max | 12 | 0.829956 | hypothetical protein |
| GLYMA_12G011400 | 1.01E+08 | protein_coding | Glycine max | 12 | 0.831481 | hypothetical protein |
| GLYMA_12G011500 | NA | protein_coding | Glycine max | 12 | 0.833322 | hypothetical protein |
| GLYMA_12G011600 | NA | protein_coding | Glycine max | 12 | 0.847958 | hypothetical protein |
| GLYMA_12G011700 | 1.01E+08 | protein_coding | Glycine max | 12 | 0.853112 | hypothetical protein |
| GLYMA_12G011800 | 1.01E+08 | protein_coding | Glycine max | 12 | 0.857542 | hypothetical protein |
| GLYMA_12G011900 | NA | protein_coding | Glycine max | 12 | 0.870008 | hypothetical protein |
| GLYMA_12G012000 | 1.01E+08 | protein_coding | Glycine max | 12 | 0.873907 | hypothetical protein |
| GLYMA_12G012100 | NA | protein_coding | Glycine max | 12 | 0.890075 | hypothetical protein |
| GLYMA_12G012200 | 732596 | protein_coding | Glycine max | 12 | 0.895961 | hypothetical protein |
| GLYMA_12G012300 | NA | protein_coding | Glycine max | 12 | 0.900156 | hypothetical protein |
| GLYMA_12G012400 | 1.01E+08 | protein_coding | Glycine max | 12 | 0.904135 | hypothetical protein |
| GLYMA_12G012500 | 1.01E+08 | protein_coding | Glycine max | 12 | 0.915303 | hypothetical protein |
| GLYMA_12G012600 | NA | protein_coding | Glycine max | 12 | 0.918419 | hypothetical protein |
| GLYMA_12G012700 | 1E+08 | protein_coding | Glycine max | 12 | 0.920004 | hypothetical protein |
| GLYMA_12G012800 | 1.01E+08 | protein_coding | Glycine max | 12 | 0.925017 | hypothetical protein |
| GLYMA_12G012900 | NA | protein_coding | Glycine max | 12 | 0.928448 | hypothetical protein |
| GLYMA_12G013000 | 1.01E+08 | protein_coding | Glycine max | 12 | 0.934229 | hypothetical protein |
| GLYMA_12G013100 | NA | protein_coding | Glycine max | 12 | 0.946483 | hypothetical protein |
| GLYMA_12G013200 | 1.01E+08 | protein_coding | Glycine max | 12 | 0.955725 | hypothetical protein |
| GLYMA_12G013300 | 1.01E+08 | protein_coding | Glycine max | 12 | 0.964404 | hypothetical protein |
| GLYMA_12G013400 | 1.01E+08 | protein_coding | Glycine max | 12 | 0.970258 | hypothetical protein |
| GLYMA_12G013500 | NA | protein_coding | Glycine max | 12 | 0.976612 | hypothetical protein |
| GLYMA_12G013600 | 1.01E+08 | protein_coding | Glycine max | 12 | 0.980833 | hypothetical protein |
| GLYMA_12G013700 | NA | protein_coding | Glycine max | 12 | 0.996838 | hypothetical protein |
| GLYMA_12G013800 | 1.01E+08 | protein_coding | Glycine max | 12 | 0.999082 | hypothetical protein |
| GLYMA_12G013900 | 1.03E+08 | protein_coding | Glycine max | 12 | 1.005754 | hypothetical protein |
| GLYMA_12G014000 | 1.01E+08 | protein_coding | Glycine max | 12 | 1.010585 | hypothetical protein |
| GLYMA_12G014100 | 1.01E+08 | protein_coding | Glycine max | 12 | 1.018924 | hypothetical protein |
| GLYMA_12G014200 | NA | protein_coding | Glycine max | 12 | 1.025584 | hypothetical protein |
| GLYMA_12G014300 | 732543 | protein_coding | Glycine max | 12 | 1.033151 | hypothetical protein |
| GLYMA_12G014400 | 1.01E+08 | protein_coding | Glycine max | 12 | 1.039859 | hypothetical protein |
| GLYMA_12G014500 | NA | protein_coding | Glycine max | 12 | 1.042638 | hypothetical protein |
| GLYMA_12G014700 | 1.01E+08 | protein_coding | Glycine max | 12 | 1.066217 | hypothetical protein |
| GLYMA_12G014800 | 1.01E+08 | protein_coding | Glycine max | 12 | 1.071368 | hypothetical protein |
| GLYMA_12G014900 | 1.01E+08 | protein_coding | Glycine max | 12 | 1.080511 | hypothetical protein |
| GLYMA_12G015000 | NA | protein_coding | Glycine max | 12 | 1.090328 | hypothetical protein |
| GLYMA_12G015100 | 1.01E+08 | protein_coding | Glycine max | 12 | 1.092653 | hypothetical protein |
| GLYMA_12G015200 | NA | protein_coding | Glycine max | 12 | 1.097666 | hypothetical protein |
| GLYMA_12G015300 | NA | protein_coding | Glycine max | 12 | 1.100879 | hypothetical protein |
| GLYMA_12G015400 | 1.01E+08 | protein_coding | Glycine max | 12 | 1.105883 | hypothetical protein |
| GLYMA_12G015500 | NA | protein_coding | Glycine max | 12 | 1.112166 | hypothetical protein |
| GLYMA_12G015600 | NA | protein_coding | Glycine max | 12 | 1.117363 | hypothetical protein |
| GLYMA_12G015700 | 1.01E+08 | protein_coding | Glycine max | 12 | 1.122541 | hypothetical protein |
| GLYMA_12G015800 | 1.01E+08 | protein_coding | Glycine max | 12 | 1.12628 | hypothetical protein |
| GLYMA_12G015900 | 1.01E+08 | protein_coding | Glycine max | 12 | 1.1369 | hypothetical protein |
| GLYMA_12G016000 | NA | protein_coding | Glycine max | 12 | 1.142873 | hypothetical protein |
| GLYMA_12G016100 | 1.01E+08 | protein_coding | Glycine max | 12 | 1.144398 | hypothetical protein |
| GLYMA_12G016200 | NA | protein_coding | Glycine max | 12 | 1.144638 | hypothetical protein |
| GLYMA_12G016300 | 1.01E+08 | protein_coding | Glycine max | 12 | 1.153697 | hypothetical protein |
| GLYMA_12G016400 | 1.01E+08 | protein_coding | Glycine max | 12 | 1.162423 | hypothetical protein |
| GLYMA_12G016500 | 1.01E+08 | protein_coding | Glycine max | 12 | 1.169676 | hypothetical protein |
| GLYMA_12G016600 | 1.01E+08 | protein_coding | Glycine max | 12 | 1.175266 | hypothetical protein |
| GLYMA_12G016700 | 1.01E+08 | protein_coding | Glycine max | 12 | 1.184026 | hypothetical protein |
| GLYMA_12G016800 | NA | protein_coding | Glycine max | 12 | 1.184312 | hypothetical protein |
| GLYMA_12G016900 | 1.01E+08 | protein_coding | Glycine max | 12 | 1.193194 | hypothetical protein |
| GLYMA_12G017000 | NA | protein_coding | Glycine max | 12 | 1.199781 | hypothetical protein |
| GLYMA_12G017100 | NA | protein_coding | Glycine max | 12 | 1.206937 | hypothetical protein |
| GLYMA_12G017200 | NA | protein_coding | Glycine max | 12 | 1.212436 | hypothetical protein |
| GLYMA_12G017400 | NA | protein_coding | Glycine max | 12 | 1.22306 | hypothetical protein |
| GLYMA_12G017500 | 1.01E+08 | protein_coding | Glycine max | 12 | 1.226333 | hypothetical protein |
| GLYMA_12G017600 | NA | protein_coding | Glycine max | 12 | 1.232608 | hypothetical protein |
| GLYMA_12G017700 | 1.01E+08 | protein_coding | Glycine max | 12 | 1.240464 | hypothetical protein |
| GLYMA_12G017800 | NA | protein_coding | Glycine max | 12 | 1.245635 | hypothetical protein |
| GLYMA_12G017900 | NA | protein_coding | Glycine max | 12 | 1.249545 | hypothetical protein |
| GLYMA_12G018000 | 1.01E+08 | protein_coding | Glycine max | 12 | 1.252709 | hypothetical protein |
| GLYMA_12G018100 | 1.01E+08 | protein_coding | Glycine max | 12 | 1.265071 | hypothetical protein |
| GLYMA_12G018200 | 1.01E+08 | protein_coding | Glycine max | 12 | 1.271081 | hypothetical protein |
| GLYMA_12G018300 | 1.01E+08 | protein_coding | Glycine max | 12 | 1.285063 | hypothetical protein |
| GLYMA_12G018400 | 1.01E+08 | protein_coding | Glycine max | 12 | 1.291197 | hypothetical protein |
| GLYMA_12G018500 | 1.01E+08 | protein_coding | Glycine max | 12 | 1.299362 | hypothetical protein |
| GLYMA_12G018600 | 1.01E+08 | protein_coding | Glycine max | 12 | 1.312778 | hypothetical protein |
| GLYMA_12G018700 | NA | protein_coding | Glycine max | 12 | 1.317977 | hypothetical protein |
| GLYMA_12G018800 | NA | protein_coding | Glycine max | 12 | 1.321415 | hypothetical protein |
| GLYMA_12G079600 | NA | protein_coding | Glycine max | 12 | 6.236413 | hypothetical protein |
| GLYMA_12G079700 | NA | protein_coding | Glycine max | 12 | 6.24194 | hypothetical protein |
| GLYMA_12G079800 | NA | protein_coding | Glycine max | 12 | 6.254464 | hypothetical protein |
| GLYMA_12G079900 | 1.01E+08 | protein_coding | Glycine max | 12 | 6.268361 | hypothetical protein |
| GLYMA_12G080000 | 1.01E+08 | protein_coding | Glycine max | 12 | 6.283385 | hypothetical protein |
| GLYMA_12G080100 | 1.01E+08 | protein_coding | Glycine max | 12 | 6.295519 | hypothetical protein |
| GLYMA_12G080200 | NA | protein_coding | Glycine max | 12 | 6.309349 | hypothetical protein |
| GLYMA_12G080300 | 1.01E+08 | protein_coding | Glycine max | 12 | 6.311283 | hypothetical protein |
| GLYMA_12G080400 | NA | protein_coding | Glycine max | 12 | 6.325267 | hypothetical protein |
| GLYMA_12G080500 | 1.01E+08 | protein_coding | Glycine max | 12 | 6.330004 | hypothetical protein |
| GLYMA_12G080600 | 1.01E+08 | protein_coding | Glycine max | 12 | 6.349138 | hypothetical protein |
| GLYMA_12G080700 | 1.01E+08 | protein_coding | Glycine max | 12 | 6.35614 | hypothetical protein |
| GLYMA_12G080800 | 1.01E+08 | protein_coding | Glycine max | 12 | 6.376727 | hypothetical protein |
| GLYMA_12G080900 | 1.01E+08 | protein_coding | Glycine max | 12 | 6.380239 | hypothetical protein |
| GLYMA_12G081000 | NA | protein_coding | Glycine max | 12 | 6.396346 | hypothetical protein |
| GLYMA_12G081100 | NA | protein_coding | Glycine max | 12 | 6.410431 | hypothetical protein |
| GLYMA_12G081200 | NA | protein_coding | Glycine max | 12 | 6.419681 | hypothetical protein |
| GLYMA_12G081300 | 1.01E+08 | protein_coding | Glycine max | 12 | 6.423237 | hypothetical protein |
| GLYMA_12G081400 | 1.01E+08 | protein_coding | Glycine max | 12 | 6.444959 | hypothetical protein |
| GLYMA_12G081500 | NA | protein_coding | Glycine max | 12 | 6.451078 | hypothetical protein |
| GLYMA_12G081600 | NA | protein_coding | Glycine max | 12 | 6.457418 | hypothetical protein |
| GLYMA_12G081700 | 1.01E+08 | protein_coding | Glycine max | 12 | 6.461033 | hypothetical protein |
| GLYMA_12G081800 | NA | protein_coding | Glycine max | 12 | 6.468179 | hypothetical protein |
| GLYMA_12G081900 | 732642 | protein_coding | Glycine max | 12 | 6.477881 | hypothetical protein |
| GLYMA_12G082000 | NA | protein_coding | Glycine max | 12 | 6.491205 | hypothetical protein |
| GLYMA_12G082100 | 778121 | protein_coding | Glycine max | 12 | 6.503492 | hypothetical protein |
| GLYMA_12G082200 | 1.01E+08 | protein_coding | Glycine max | 12 | 6.512661 | hypothetical protein |
| GLYMA_12G082300 | 1.01E+08 | protein_coding | Glycine max | 12 | 6.515173 | hypothetical protein |
| GLYMA_12G082400 | 1.01E+08 | protein_coding | Glycine max | 12 | 6.534333 | hypothetical protein |
| GLYMA_12G082500 | NA | protein_coding | Glycine max | 12 | 6.5408 | hypothetical protein |
| GLYMA_12G082600 | NA | protein_coding | Glycine max | 12 | 6.54954 | hypothetical protein |
| GLYMA_12G082700 | 1.01E+08 | protein_coding | Glycine max | 12 | 6.554176 | hypothetical protein |
| GLYMA_12G082800 | NA | protein_coding | Glycine max | 12 | 6.558581 | hypothetical protein |
| GLYMA_12G082900 | NA | protein_coding | Glycine max | 12 | 6.561398 | hypothetical protein |
| GLYMA_12G083000 | 1.01E+08 | protein_coding | Glycine max | 12 | 6.566877 | hypothetical protein |
| GLYMA_12G083100 | 1.01E+08 | protein_coding | Glycine max | 12 | 6.586692 | hypothetical protein |
| GLYMA_12G083200 | 1.01E+08 | protein_coding | Glycine max | 12 | 6.594911 | hypothetical protein |
| GLYMA_12G083300 | 1.01E+08 | protein_coding | Glycine max | 12 | 6.617631 | hypothetical protein |
| GLYMA_12G083400 | NA | protein_coding | Glycine max | 12 | 6.625486 | hypothetical protein |
| GLYMA_12G083500 | 1.01E+08 | protein_coding | Glycine max | 12 | 6.649319 | hypothetical protein |
| GLYMA_12G083600 | 1.01E+08 | protein_coding | Glycine max | 12 | 6.657383 | hypothetical protein |
| GLYMA_12G083800 | 1.01E+08 | protein_coding | Glycine max | 12 | 6.687696 | hypothetical protein |
| GLYMA_12G083900 | NA | protein_coding | Glycine max | 12 | 6.695874 | hypothetical protein |
| GLYMA_12G084000 | 1.01E+08 | protein_coding | Glycine max | 12 | 6.700667 | hypothetical protein |
| GLYMA_12G084100 | NA | protein_coding | Glycine max | 12 | 6.71953 | hypothetical protein |
| GLYMA_13G044100 | 1.01E+08 | protein_coding | Glycine max | 13 | 13.758232 | hypothetical protein |
| GLYMA_13G044200 | 1.01E+08 | protein_coding | Glycine max | 13 | 13.768892 | hypothetical protein |
| GLYMA_13G044300 | 1.01E+08 | protein_coding | Glycine max | 13 | 13.774555 | hypothetical protein |
| GLYMA_13G044400 | NA | protein_coding | Glycine max | 13 | 13.780647 | hypothetical protein |
| GLYMA_13G044500 | 1.01E+08 | protein_coding | Glycine max | 13 | 13.792197 | hypothetical protein |
| GLYMA_13G044600 | 1.01E+08 | protein_coding | Glycine max | 13 | 13.814004 | hypothetical protein |
| GLYMA_13G044700 | NA | protein_coding | Glycine max | 13 | 13.826416 | hypothetical protein |
| GLYMA_13G044800 | 1.01E+08 | protein_coding | Glycine max | 13 | 13.848454 | hypothetical protein |
| GLYMA_13G044900 | 1.01E+08 | protein_coding | Glycine max | 13 | 13.85913 | hypothetical protein |
| GLYMA_13G045000 | 1.01E+08 | protein_coding | Glycine max | 13 | 13.860998 | hypothetical protein |
| GLYMA_13G045100 | 1.01E+08 | protein_coding | Glycine max | 13 | 13.883277 | hypothetical protein |
| GLYMA_13G045200 | 1.01E+08 | protein_coding | Glycine max | 13 | 13.891291 | hypothetical protein |
| GLYMA_13G045300 | NA | protein_coding | Glycine max | 13 | 13.911395 | hypothetical protein |
| GLYMA_13G045400 | 1.01E+08 | protein_coding | Glycine max | 13 | 13.925441 | hypothetical protein |
| GLYMA_13G045500 | NA | protein_coding | Glycine max | 13 | 13.944035 | hypothetical protein |
| GLYMA_13G045600 | 1.01E+08 | protein_coding | Glycine max | 13 | 13.947013 | hypothetical protein |
| GLYMA_13G045700 | 1.01E+08 | protein_coding | Glycine max | 13 | 13.984814 | hypothetical protein |
| GLYMA_13G045800 | 1.01E+08 | protein_coding | Glycine max | 13 | 13.992003 | hypothetical protein |
| GLYMA_13G045900 | 1.01E+08 | protein_coding | Glycine max | 13 | 14.009127 | hypothetical protein |
| GLYMA_13G046000 | 1.01E+08 | protein_coding | Glycine max | 13 | 14.021814 | hypothetical protein |
| GLYMA_13G046100 | 1E+08 | protein_coding | Glycine max | 13 | 14.035748 | hypothetical protein |
| GLYMA_13G046200 | 1.01E+08 | protein_coding | Glycine max | 13 | 14.043433 | hypothetical protein |
| GLYMA_13G046300 | 1.01E+08 | protein_coding | Glycine max | 13 | 14.064352 | hypothetical protein |
| GLYMA_13G046400 | 1.01E+08 | protein_coding | Glycine max | 13 | 14.071533 | hypothetical protein |
| GLYMA_13G046500 | 1.01E+08 | protein_coding | Glycine max | 13 | 14.089339 | hypothetical protein |
| GLYMA_13G046600 | 1.01E+08 | protein_coding | Glycine max | 13 | 14.105161 | hypothetical protein |
| GLYMA_13G046700 | 1.01E+08 | protein_coding | Glycine max | 13 | 14.127899 | hypothetical protein |
| GLYMA_13G046800 | 1.01E+08 | protein_coding | Glycine max | 13 | 14.134396 | hypothetical protein |
| GLYMA_13G046900 | 1.01E+08 | protein_coding | Glycine max | 13 | 14.162698 | hypothetical protein |
| GLYMA_13G047000 | NA | protein_coding | Glycine max | 13 | 14.170795 | hypothetical protein |
| GLYMA_13G047100 | NA | protein_coding | Glycine max | 13 | 14.172381 | hypothetical protein |
| GLYMA_13G047200 | NA | protein_coding | Glycine max | 13 | 14.190107 | hypothetical protein |
| GLYMA_13G047300 | 1.01E+08 | protein_coding | Glycine max | 13 | 14.193855 | hypothetical protein |
| GLYMA_13G082400 | 1.01E+08 | protein_coding | Glycine max | 13 | 19.195731 | hypothetical protein |
| GLYMA_13G082500 | NA | protein_coding | Glycine max | 13 | 19.214373 | hypothetical protein |
| GLYMA_13G082600 | NA | protein_coding | Glycine max | 13 | 19.219866 | hypothetical protein |
| GLYMA_13G082700 | 1.01E+08 | protein_coding | Glycine max | 13 | 19.225672 | hypothetical protein |
| GLYMA_13G082800 | NA | protein_coding | Glycine max | 13 | 19.239515 | hypothetical protein |
| GLYMA_13G082900 | NA | protein_coding | Glycine max | 13 | 19.241622 | hypothetical protein |
| GLYMA_13G083000 | 1.01E+08 | protein_coding | Glycine max | 13 | 19.257367 | hypothetical protein |
| GLYMA_13G083100 | 1.01E+08 | protein_coding | Glycine max | 13 | 19.285544 | hypothetical protein |
| GLYMA_13G083200 | NA | protein_coding | Glycine max | 13 | 19.303452 | hypothetical protein |
| GLYMA_13G083300 | NA | protein_coding | Glycine max | 13 | 19.306837 | hypothetical protein |
| GLYMA_13G083400 | 1.01E+08 | protein_coding | Glycine max | 13 | 19.399107 | hypothetical protein |
| GLYMA_13G083500 | 1.03E+08 | protein_coding | Glycine max | 13 | 19.425108 | hypothetical protein |
| GLYMA_13G083600 | NA | protein_coding | Glycine max | 13 | 19.436261 | hypothetical protein |
| GLYMA_13G083700 | 1.01E+08 | protein_coding | Glycine max | 13 | 19.442707 | hypothetical protein |
| GLYMA_13G083800 | NA | protein_coding | Glycine max | 13 | 19.444299 | hypothetical protein |
| GLYMA_13G083900 | NA | protein_coding | Glycine max | 13 | 19.460488 | hypothetical protein |
| GLYMA_13G084000 | NA | protein_coding | Glycine max | 13 | 19.466653 | hypothetical protein |
| GLYMA_13G084100 | 1.01E+08 | protein_coding | Glycine max | 13 | 19.516514 | hypothetical protein |
| GLYMA_13G084200 | NA | protein_coding | Glycine max | 13 | 19.526937 | hypothetical protein |
| GLYMA_13G084300 | 1.01E+08 | protein_coding | Glycine max | 13 | 19.543443 | hypothetical protein |
| GLYMA_13G084400 | NA | protein_coding | Glycine max | 13 | 19.549504 | hypothetical protein |
| GLYMA_13G084500 | NA | protein_coding | Glycine max | 13 | 19.562938 | hypothetical protein |
| GLYMA_13G084600 | 1.01E+08 | protein_coding | Glycine max | 13 | 19.574202 | hypothetical protein |
| GLYMA_13G084700 | 1.01E+08 | protein_coding | Glycine max | 13 | 19.586384 | hypothetical protein |
| GLYMA_13G084800 | 1.01E+08 | protein_coding | Glycine max | 13 | 19.591484 | hypothetical protein |
| GLYMA_13G084900 | NA | protein_coding | Glycine max | 13 | 19.610372 | hypothetical protein |
| GLYMA_13G085000 | NA | protein_coding | Glycine max | 13 | 19.615152 | hypothetical protein |
| GLYMA_13G085100 | 1.01E+08 | protein_coding | Glycine max | 13 | 19.659235 | hypothetical protein |
| GLYMA_13G085200 | NA | protein_coding | Glycine max | 13 | 19.667765 | hypothetical protein |
| GLYMA_14G009300 | NA | protein_coding | Glycine max | 14 | 0.728111 | hypothetical protein |
| GLYMA_14G009400 | 1.01E+08 | protein_coding | Glycine max | 14 | 0.73632 | hypothetical protein |
| GLYMA_14G009500 | NA | protein_coding | Glycine max | 14 | 0.741433 | hypothetical protein |
| GLYMA_14G009600 | 1.01E+08 | protein_coding | Glycine max | 14 | 0.745925 | hypothetical protein |
| GLYMA_14G009700 | 1.01E+08 | protein_coding | Glycine max | 14 | 0.754004 | hypothetical protein |
| GLYMA_14G009800 | NA | protein_coding | Glycine max | 14 | 0.762881 | hypothetical protein |
| GLYMA_14G010000 | NA | protein_coding | Glycine max | 14 | 0.773013 | hypothetical protein |
| GLYMA_14G010100 | NA | protein_coding | Glycine max | 14 | 0.777671 | hypothetical protein |
| GLYMA_14G010200 | 1.01E+08 | protein_coding | Glycine max | 14 | 0.788305 | hypothetical protein |
| GLYMA_14G010300 | NA | protein_coding | Glycine max | 14 | 0.793295 | hypothetical protein |
| GLYMA_14G010400 | 1.01E+08 | protein_coding | Glycine max | 14 | 0.802172 | hypothetical protein |
| GLYMA_14G010500 | 1.01E+08 | protein_coding | Glycine max | 14 | 0.807239 | hypothetical protein |
| GLYMA_14G010600 | 1.01E+08 | protein_coding | Glycine max | 14 | 0.817937 | hypothetical protein |
| GLYMA_14G010700 | 1.03E+08 | protein_coding | Glycine max | 14 | 0.82033 | hypothetical protein |
| GLYMA_14G010800 | 1.01E+08 | protein_coding | Glycine max | 14 | 0.824392 | hypothetical protein |
| GLYMA_14G010900 | 1.01E+08 | protein_coding | Glycine max | 14 | 0.827469 | hypothetical protein |
| GLYMA_14G011000 | 1.01E+08 | protein_coding | Glycine max | 14 | 0.830247 | hypothetical protein |
| GLYMA_14G011100 | 1.01E+08 | protein_coding | Glycine max | 14 | 0.84767 | hypothetical protein |
| GLYMA_14G011200 | 1.07E+08 | protein_coding | Glycine max | 14 | 0.858328 | hypothetical protein |
| GLYMA_14G011300 | 1.01E+08 | protein_coding | Glycine max | 14 | 0.860396 | hypothetical protein |
| GLYMA_14G011400 | NA | protein_coding | Glycine max | 14 | 0.863299 | hypothetical protein |
| GLYMA_14G011500 | 1.01E+08 | protein_coding | Glycine max | 14 | 0.868065 | hypothetical protein |
| GLYMA_14G011600 | 1.01E+08 | protein_coding | Glycine max | 14 | 0.887156 | hypothetical protein |
| GLYMA_14G011700 | NA | protein_coding | Glycine max | 14 | 0.891533 | hypothetical protein |
| GLYMA_14G011800 | 1.01E+08 | protein_coding | Glycine max | 14 | 0.893299 | hypothetical protein |
| GLYMA_14G011900 | 1.01E+08 | protein_coding | Glycine max | 14 | 0.902341 | hypothetical protein |
| GLYMA_14G012000 | 1.01E+08 | protein_coding | Glycine max | 14 | 0.906742 | hypothetical protein |
| GLYMA_14G012100 | 1.01E+08 | protein_coding | Glycine max | 14 | 0.914315 | hypothetical protein |
| GLYMA_14G012200 | 1.01E+08 | protein_coding | Glycine max | 14 | 0.920087 | hypothetical protein |
| GLYMA_14G012300 | 1E+08 | protein_coding | Glycine max | 14 | 0.929001 | hypothetical protein |
| GLYMA_14G012400 | 1.01E+08 | protein_coding | Glycine max | 14 | 0.93181 | hypothetical protein |
| GLYMA_14G012500 | NA | protein_coding | Glycine max | 14 | 0.941248 | hypothetical protein |
| GLYMA_14G012600 | 1.01E+08 | protein_coding | Glycine max | 14 | 0.94648 | hypothetical protein |
| GLYMA_14G012700 | 1.01E+08 | protein_coding | Glycine max | 14 | 0.950377 | hypothetical protein |
| GLYMA_14G012800 | NA | protein_coding | Glycine max | 14 | 0.96229 | hypothetical protein |
| GLYMA_14G012900 | NA | protein_coding | Glycine max | 14 | 0.971984 | hypothetical protein |
| GLYMA_14G013000 | 1.01E+08 | protein_coding | Glycine max | 14 | 0.979943 | hypothetical protein |
| GLYMA_14G013100 | NA | protein_coding | Glycine max | 14 | 0.983795 | hypothetical protein |
| GLYMA_14G013200 | NA | protein_coding | Glycine max | 14 | 0.990726 | hypothetical protein |
| GLYMA_14G013300 | NA | protein_coding | Glycine max | 14 | 0.998854 | hypothetical protein |
| GLYMA_14G013400 | 1.01E+08 | protein_coding | Glycine max | 14 | 1.000834 | hypothetical protein |
| GLYMA_14G013500 | NA | protein_coding | Glycine max | 14 | 1.009519 | hypothetical protein |
| GLYMA_14G013600 | NA | protein_coding | Glycine max | 14 | 1.015115 | hypothetical protein |
| GLYMA_14G013700 | 1.01E+08 | protein_coding | Glycine max | 14 | 1.018079 | hypothetical protein |
| GLYMA_14G013800 | NA | protein_coding | Glycine max | 14 | 1.02352 | hypothetical protein |
| GLYMA_14G013900 | 1.01E+08 | protein_coding | Glycine max | 14 | 1.027501 | hypothetical protein |
| GLYMA_14G014000 | 1.01E+08 | protein_coding | Glycine max | 14 | 1.029711 | hypothetical protein |
| GLYMA_14G014100 | 1.01E+08 | protein_coding | Glycine max | 14 | 1.033723 | hypothetical protein |
| GLYMA_14G014200 | 1.01E+08 | protein_coding | Glycine max | 14 | 1.042883 | hypothetical protein |
| GLYMA_14G014300 | NA | protein_coding | Glycine max | 14 | 1.045063 | hypothetical protein |
| GLYMA_14G014400 | 1.01E+08 | protein_coding | Glycine max | 14 | 1.049877 | hypothetical protein |
| GLYMA_14G014500 | NA | protein_coding | Glycine max | 14 | 1.05771 | hypothetical protein |
| GLYMA_14G014600 | NA | protein_coding | Glycine max | 14 | 1.062756 | hypothetical protein |
| GLYMA_14G014700 | 1.01E+08 | protein_coding | Glycine max | 14 | 1.06872 | hypothetical protein |
| GLYMA_14G014800 | 1.01E+08 | protein_coding | Glycine max | 14 | 1.071887 | hypothetical protein |
| GLYMA_14G014900 | NA | protein_coding | Glycine max | 14 | 1.077772 | hypothetical protein |
| GLYMA_14G015000 | NA | protein_coding | Glycine max | 14 | 1.081537 | hypothetical protein |
| GLYMA_14G015100 | 1.01E+08 | protein_coding | Glycine max | 14 | 1.085839 | hypothetical protein |
| GLYMA_14G015200 | 1.01E+08 | protein_coding | Glycine max | 14 | 1.089998 | hypothetical protein |
| GLYMA_14G015300 | 1.01E+08 | protein_coding | Glycine max | 14 | 1.100698 | hypothetical protein |
| GLYMA_14G015400 | NA | protein_coding | Glycine max | 14 | 1.109556 | hypothetical protein |
| GLYMA_14G015500 | NA | protein_coding | Glycine max | 14 | 1.110972 | hypothetical protein |
| GLYMA_14G015600 | 1.01E+08 | protein_coding | Glycine max | 14 | 1.115792 | hypothetical protein |
| GLYMA_14G015700 | 1.01E+08 | protein_coding | Glycine max | 14 | 1.130025 | hypothetical protein |
| GLYMA_14G015800 | 1.03E+08 | protein_coding | Glycine max | 14 | 1.138621 | hypothetical protein |
| GLYMA_14G015900 | 1.01E+08 | protein_coding | Glycine max | 14 | 1.141714 | hypothetical protein |
| GLYMA_14G016000 | 1.01E+08 | protein_coding | Glycine max | 14 | 1.147572 | hypothetical protein |
| GLYMA_14G016100 | 1.01E+08 | protein_coding | Glycine max | 14 | 1.15098 | hypothetical protein |
| GLYMA_14G016200 | NA | protein_coding | Glycine max | 14 | 1.154838 | hypothetical protein |
| GLYMA_14G016300 | 1.01E+08 | protein_coding | Glycine max | 14 | 1.170626 | hypothetical protein |
| GLYMA_14G016400 | 1.01E+08 | protein_coding | Glycine max | 14 | 1.177818 | hypothetical protein |
| GLYMA_14G016500 | NA | protein_coding | Glycine max | 14 | 1.183339 | hypothetical protein |
| GLYMA_14G016600 | 1.01E+08 | protein_coding | Glycine max | 14 | 1.188616 | hypothetical protein |
| GLYMA_14G016700 | 1.01E+08 | protein_coding | Glycine max | 14 | 1.196571 | hypothetical protein |
| GLYMA_14G016800 | 1.01E+08 | protein_coding | Glycine max | 14 | 1.206662 | hypothetical protein |
| GLYMA_14G016900 | NA | protein_coding | Glycine max | 14 | 1.213715 | hypothetical protein |
| GLYMA_14G017000 | 1.01E+08 | protein_coding | Glycine max | 14 | 1.218416 | hypothetical protein |
| GLYMA_14G066800 | NA | protein_coding | Glycine max | 14 | 5.511222 | hypothetical protein |
| GLYMA_14G066900 | NA | protein_coding | Glycine max | 14 | 5.528647 | hypothetical protein |
| GLYMA_14G067000 | NA | protein_coding | Glycine max | 14 | 5.536332 | hypothetical protein |
| GLYMA_14G067100 | 1.01E+08 | protein_coding | Glycine max | 14 | 5.552993 | hypothetical protein |
| GLYMA_14G067200 | 1.01E+08 | protein_coding | Glycine max | 14 | 5.56799 | hypothetical protein |
| GLYMA_14G067300 | 1.01E+08 | protein_coding | Glycine max | 14 | 5.574711 | hypothetical protein |
| GLYMA_14G067400 | 1.01E+08 | protein_coding | Glycine max | 14 | 5.582925 | hypothetical protein |
| GLYMA_14G067500 | 1.01E+08 | protein_coding | Glycine max | 14 | 5.596952 | hypothetical protein |
| GLYMA_14G067600 | NA | protein_coding | Glycine max | 14 | 5.658765 | hypothetical protein |
| GLYMA_14G067700 | NA | protein_coding | Glycine max | 14 | 5.672578 | hypothetical protein |
| GLYMA_14G067800 | 1.01E+08 | protein_coding | Glycine max | 14 | 5.675792 | hypothetical protein |
| GLYMA_14G067900 | 1.01E+08 | protein_coding | Glycine max | 14 | 5.680978 | hypothetical protein |
| GLYMA_14G068000 | 1.01E+08 | protein_coding | Glycine max | 14 | 5.697534 | hypothetical protein |
| GLYMA_14G068100 | 1.01E+08 | protein_coding | Glycine max | 14 | 5.701618 | hypothetical protein |
| GLYMA_14G068200 | 1.01E+08 | protein_coding | Glycine max | 14 | 5.713033 | hypothetical protein |
| GLYMA_14G068300 | 1.01E+08 | protein_coding | Glycine max | 14 | 5.723609 | hypothetical protein |
| GLYMA_14G068400 | 1.01E+08 | protein_coding | Glycine max | 14 | 5.732278 | hypothetical protein |
| GLYMA_14G068500 | 1.01E+08 | protein_coding | Glycine max | 14 | 5.741909 | hypothetical protein |
| GLYMA_14G068600 | 1.01E+08 | protein_coding | Glycine max | 14 | 5.748827 | hypothetical protein |
| GLYMA_14G068700 | NA | protein_coding | Glycine max | 14 | 5.754217 | hypothetical protein |
| GLYMA_14G068800 | 1.01E+08 | protein_coding | Glycine max | 14 | 5.764497 | hypothetical protein |
| GLYMA_14G068900 | 1.01E+08 | protein_coding | Glycine max | 14 | 5.775416 | hypothetical protein |
| GLYMA_14G069000 | NA | protein_coding | Glycine max | 14 | 5.780212 | hypothetical protein |
| GLYMA_14G069100 | NA | protein_coding | Glycine max | 14 | 5.788089 | hypothetical protein |
| GLYMA_14G069200 | 1.01E+08 | protein_coding | Glycine max | 14 | 5.801924 | hypothetical protein |
| GLYMA_14G069300 | 1E+08 | protein_coding | Glycine max | 14 | 5.811019 | hypothetical protein |
| GLYMA_14G069400 | NA | protein_coding | Glycine max | 14 | 5.821099 | hypothetical protein |
| GLYMA_14G069500 | 1.01E+08 | protein_coding | Glycine max | 14 | 5.822246 | hypothetical protein |
| GLYMA_14G069600 | 1.01E+08 | protein_coding | Glycine max | 14 | 5.828443 | hypothetical protein |
| GLYMA_14G069700 | 1.07E+08 | protein_coding | Glycine max | 14 | 5.843959 | hypothetical protein |
| GLYMA_14G069800 | 1.01E+08 | protein_coding | Glycine max | 14 | 5.85494 | hypothetical protein |
| GLYMA_14G069900 | 1.03E+08 | protein_coding | Glycine max | 14 | 5.869557 | hypothetical protein |
| GLYMA_14G070000 | 1.01E+08 | protein_coding | Glycine max | 14 | 5.884058 | hypothetical protein |
| GLYMA_14G070100 | 1.01E+08 | protein_coding | Glycine max | 14 | 5.887077 | hypothetical protein |
| GLYMA_14G070200 | 1.03E+08 | protein_coding | Glycine max | 14 | 5.89945 | hypothetical protein |
| GLYMA_14G070300 | 1.01E+08 | protein_coding | Glycine max | 14 | 5.905698 | hypothetical protein |
| GLYMA_14G070400 | 1.01E+08 | protein_coding | Glycine max | 14 | 5.910263 | hypothetical protein |
| GLYMA_14G070500 | NA | protein_coding | Glycine max | 14 | 5.918611 | hypothetical protein |
| GLYMA_14G070600 | 1.01E+08 | protein_coding | Glycine max | 14 | 5.921265 | hypothetical protein |
| GLYMA_14G070700 | 1.01E+08 | protein_coding | Glycine max | 14 | 5.948468 | hypothetical protein |
| GLYMA_14G070800 | NA | protein_coding | Glycine max | 14 | 5.962564 | hypothetical protein |
| GLYMA_14G070900 | NA | protein_coding | Glycine max | 14 | 5.966147 | hypothetical protein |
| GLYMA_14G071000 | 1.01E+08 | protein_coding | Glycine max | 14 | 5.972251 | hypothetical protein |
| GLYMA_14G071100 | 1.01E+08 | protein_coding | Glycine max | 14 | 5.975838 | hypothetical protein |
| GLYMA_14G071200 | NA | protein_coding | Glycine max | 14 | 5.981031 | hypothetical protein |
| GLYMA_14G071300 | 1.01E+08 | protein_coding | Glycine max | 14 | 5.986366 | hypothetical protein |
| GLYMA_14G071400 | 1E+08 | protein_coding | Glycine max | 14 | 5.992881 | hypothetical protein |
| GLYMA_14G071500 | NA | protein_coding | Glycine max | 14 | 5.994186 | hypothetical protein |
| GLYMA_14G120900 | 1.01E+08 | protein_coding | Glycine max | 14 | 17.400748 | hypothetical protein |
| GLYMA_14G121000 | NA | protein_coding | Glycine max | 14 | 17.429115 | hypothetical protein |
| GLYMA_14G121100 | NA | protein_coding | Glycine max | 14 | 17.454361 | hypothetical protein |
| GLYMA_14G121200 | 1.01E+08 | protein_coding | Glycine max | 14 | 17.460374 | hypothetical protein |
| GLYMA_14G121300 | NA | protein_coding | Glycine max | 14 | 17.472203 | hypothetical protein |
| GLYMA_14G121400 | NA | protein_coding | Glycine max | 14 | 17.499717 | hypothetical protein |
| GLYMA_14G121500 | NA | protein_coding | Glycine max | 14 | 17.529809 | hypothetical protein |
| GLYMA_14G121600 | NA | protein_coding | Glycine max | 14 | 17.544093 | hypothetical protein |
| GLYMA_14G121700 | NA | protein_coding | Glycine max | 14 | 17.550558 | hypothetical protein |
| GLYMA_14G121800 | 1.01E+08 | protein_coding | Glycine max | 14 | 17.612743 | hypothetical protein |
| GLYMA_14G121900 | NA | protein_coding | Glycine max | 14 | 17.673707 | hypothetical protein |
| GLYMA_14G122000 | NA | protein_coding | Glycine max | 14 | 17.678837 | hypothetical protein |
| GLYMA_14G122100 | NA | protein_coding | Glycine max | 14 | 17.7782 | hypothetical protein |
| GLYMA_14G122200 | 1.01E+08 | protein_coding | Glycine max | 14 | 17.801426 | hypothetical protein |
| GLYMA_15G115400 | 1.01E+08 | protein_coding | Glycine max | 15 | 9.081677 | hypothetical protein |
| GLYMA_15G115500 | NA | protein_coding | Glycine max | 15 | 9.087902 | hypothetical protein |
| GLYMA_15G115600 | 1.01E+08 | protein_coding | Glycine max | 15 | 9.093454 | hypothetical protein |
| GLYMA_15G115700 | 1.03E+08 | protein_coding | Glycine max | 15 | 9.108067 | hypothetical protein |
| GLYMA_15G115800 | 1.01E+08 | protein_coding | Glycine max | 15 | 9.118834 | hypothetical protein |
| GLYMA_15G115900 | NA | protein_coding | Glycine max | 15 | 9.121807 | hypothetical protein |
| GLYMA_15G116000 | 1.01E+08 | protein_coding | Glycine max | 15 | 9.130584 | hypothetical protein |
| GLYMA_15G116100 | NA | protein_coding | Glycine max | 15 | 9.135197 | hypothetical protein |
| GLYMA_15G116200 | 1.01E+08 | protein_coding | Glycine max | 15 | 9.136796 | hypothetical protein |
| GLYMA_15G116300 | 1.01E+08 | protein_coding | Glycine max | 15 | 9.141617 | hypothetical protein |
| GLYMA_15G116400 | NA | protein_coding | Glycine max | 15 | 9.144027 | hypothetical protein |
| GLYMA_15G116500 | 1.01E+08 | protein_coding | Glycine max | 15 | 9.160515 | hypothetical protein |
| GLYMA_15G116600 | NA | protein_coding | Glycine max | 15 | 9.163979 | hypothetical protein |
| GLYMA_15G116700 | NA | protein_coding | Glycine max | 15 | 9.176466 | hypothetical protein |
| GLYMA_15G116800 | 1.01E+08 | protein_coding | Glycine max | 15 | 9.183826 | hypothetical protein |
| GLYMA_15G116900 | 1.01E+08 | protein_coding | Glycine max | 15 | 9.190898 | hypothetical protein |
| GLYMA_15G117000 | 1.01E+08 | protein_coding | Glycine max | 15 | 9.192797 | hypothetical protein |
| GLYMA_15G117100 | 1.01E+08 | protein_coding | Glycine max | 15 | 9.200056 | hypothetical protein |
| GLYMA_15G117200 | 1.01E+08 | protein_coding | Glycine max | 15 | 9.209252 | hypothetical protein |
| GLYMA_15G117300 | NA | protein_coding | Glycine max | 15 | 9.215314 | hypothetical protein |
| GLYMA_15G117400 | NA | protein_coding | Glycine max | 15 | 9.221901 | hypothetical protein |
| GLYMA_15G117500 | 1.01E+08 | protein_coding | Glycine max | 15 | 9.225089 | hypothetical protein |
| GLYMA_15G117600 | 1.03E+08 | protein_coding | Glycine max | 15 | 9.233721 | hypothetical protein |
| GLYMA_15G117700 | NA | protein_coding | Glycine max | 15 | 9.239652 | hypothetical protein |
| GLYMA_15G117800 | NA | protein_coding | Glycine max | 15 | 9.242343 | hypothetical protein |
| GLYMA_15G117900 | NA | protein_coding | Glycine max | 15 | 9.249424 | hypothetical protein |
| GLYMA_15G118000 | 1.01E+08 | protein_coding | Glycine max | 15 | 9.255443 | hypothetical protein |
| GLYMA_15G118100 | 1.01E+08 | protein_coding | Glycine max | 15 | 9.266014 | hypothetical protein |
| GLYMA_15G118200 | 1.07E+08 | protein_coding | Glycine max | 15 | 9.269125 | hypothetical protein |
| GLYMA_15G118300 | NA | protein_coding | Glycine max | 15 | 9.273007 | hypothetical protein |
| GLYMA_15G118400 | 1.01E+08 | protein_coding | Glycine max | 15 | 9.289526 | hypothetical protein |
| GLYMA_15G118500 | 1.01E+08 | protein_coding | Glycine max | 15 | 9.299024 | hypothetical protein |
| GLYMA_15G118600 | 1.01E+08 | protein_coding | Glycine max | 15 | 9.305534 | hypothetical protein |
| GLYMA_15G118700 | 1.01E+08 | protein_coding | Glycine max | 15 | 9.311086 | hypothetical protein |
| GLYMA_15G118800 | 1.01E+08 | protein_coding | Glycine max | 15 | 9.317982 | hypothetical protein |
| GLYMA_15G118900 | 1.01E+08 | protein_coding | Glycine max | 15 | 9.322973 | hypothetical protein |
| GLYMA_15G119000 | 1.01E+08 | protein_coding | Glycine max | 15 | 9.328462 | hypothetical protein |
| GLYMA_15G119100 | 1.01E+08 | protein_coding | Glycine max | 15 | 9.340682 | hypothetical protein |
| GLYMA_15G119200 | NA | protein_coding | Glycine max | 15 | 9.38445 | hypothetical protein |
| GLYMA_15G119300 | NA | protein_coding | Glycine max | 15 | 9.4025 | hypothetical protein |
| GLYMA_15G119400 | NA | protein_coding | Glycine max | 15 | 9.404175 | hypothetical protein |
| GLYMA_15G119500 | 1.01E+08 | protein_coding | Glycine max | 15 | 9.410716 | hypothetical protein |
| GLYMA_15G119600 | 1.01E+08 | protein_coding | Glycine max | 15 | 9.42628 | hypothetical protein |
| GLYMA_15G119700 | NA | protein_coding | Glycine max | 15 | 9.433821 | hypothetical protein |
| GLYMA_15G119800 | 1.01E+08 | protein_coding | Glycine max | 15 | 9.442775 | hypothetical protein |
| GLYMA_15G119900 | NA | protein_coding | Glycine max | 15 | 9.473098 | hypothetical protein |
| GLYMA_15G120000 | NA | protein_coding | Glycine max | 15 | 9.483962 | hypothetical protein |
| GLYMA_15G120100 | 1.01E+08 | protein_coding | Glycine max | 15 | 9.502825 | hypothetical protein |
| GLYMA_15G120200 | 1.03E+08 | protein_coding | Glycine max | 15 | 9.5063 | hypothetical protein |
| GLYMA_15G120300 | 1.01E+08 | protein_coding | Glycine max | 15 | 9.512577 | hypothetical protein |
| GLYMA_15G120400 | 1.01E+08 | protein_coding | Glycine max | 15 | 9.531897 | hypothetical protein |
| GLYMA_15G120500 | NA | protein_coding | Glycine max | 15 | 9.536856 | hypothetical protein |
| GLYMA_15G120600 | NA | protein_coding | Glycine max | 15 | 9.540774 | hypothetical protein |
| GLYMA_15G120700 | 1.01E+08 | protein_coding | Glycine max | 15 | 9.54824 | hypothetical protein |
| GLYMA_15G120800 | 1.01E+08 | protein_coding | Glycine max | 15 | 9.552374 | hypothetical protein |
| GLYMA_15G120900 | NA | protein_coding | Glycine max | 15 | 9.553113 | hypothetical protein |
| GLYMA_15G121000 | 1.01E+08 | protein_coding | Glycine max | 15 | 9.563579 | hypothetical protein |
| GLYMA_15G121100 | 1.01E+08 | protein_coding | Glycine max | 15 | 9.567797 | hypothetical protein |
| GLYMA_15G121200 | NA | protein_coding | Glycine max | 15 | 9.573092 | hypothetical protein |
| GLYMA_15G272100 | 1.01E+08 | protein_coding | Glycine max | 15 | 50.944579 | hypothetical protein |
| GLYMA_15G272200 | NA | protein_coding | Glycine max | 15 | 50.951794 | hypothetical protein |
| GLYMA_15G272300 | 1.01E+08 | protein_coding | Glycine max | 15 | 50.965263 | hypothetical protein |
| GLYMA_15G272400 | 1.01E+08 | protein_coding | Glycine max | 15 | 50.978413 | hypothetical protein |
| GLYMA_15G272500 | NA | protein_coding | Glycine max | 15 | 50.993941 | hypothetical protein |
| GLYMA_15G272600 | NA | protein_coding | Glycine max | 15 | 51.00025 | hypothetical protein |
| GLYMA_15G272700 | NA | protein_coding | Glycine max | 15 | 51.00852 | hypothetical protein |
| GLYMA_15G272800 | NA | protein_coding | Glycine max | 15 | 51.021936 | hypothetical protein |
| GLYMA_15G272900 | NA | protein_coding | Glycine max | 15 | 51.030895 | hypothetical protein |
| GLYMA_15G273000 | 1.01E+08 | protein_coding | Glycine max | 15 | 51.048375 | hypothetical protein |
| GLYMA_15G273100 | NA | protein_coding | Glycine max | 15 | 51.057301 | hypothetical protein |
| GLYMA_15G273200 | NA | protein_coding | Glycine max | 15 | 51.060151 | hypothetical protein |
| GLYMA_15G273300 | 1.01E+08 | protein_coding | Glycine max | 15 | 51.069984 | hypothetical protein |
| GLYMA_15G273400 | 1.01E+08 | protein_coding | Glycine max | 15 | 51.080478 | hypothetical protein |
| GLYMA_15G273500 | 1.01E+08 | protein_coding | Glycine max | 15 | 51.101848 | hypothetical protein |
| GLYMA_15G273600 | 1.01E+08 | protein_coding | Glycine max | 15 | 51.130117 | hypothetical protein |
| GLYMA_15G273700 | 1.01E+08 | protein_coding | Glycine max | 15 | 51.138129 | hypothetical protein |
| GLYMA_15G273800 | NA | protein_coding | Glycine max | 15 | 51.142368 | hypothetical protein |
| GLYMA_15G273900 | 1.01E+08 | protein_coding | Glycine max | 15 | 51.148949 | hypothetical protein |
| GLYMA_15G274000 | 1.01E+08 | protein_coding | Glycine max | 15 | 51.156044 | hypothetical protein |
| GLYMA_15G274100 | NA | protein_coding | Glycine max | 15 | 51.18107 | hypothetical protein |
| GLYMA_15G274200 | 1.01E+08 | protein_coding | Glycine max | 15 | 51.187965 | hypothetical protein |
| GLYMA_15G274300 | 1.03E+08 | protein_coding | Glycine max | 15 | 51.194531 | hypothetical protein |
| GLYMA_15G274400 | 1.01E+08 | protein_coding | Glycine max | 15 | 51.221837 | hypothetical protein |
| GLYMA_15G274500 | NA | protein_coding | Glycine max | 15 | 51.235879 | hypothetical protein |
| GLYMA_15G274600 | 1E+08 | protein_coding | Glycine max | 15 | 51.258793 | hypothetical protein |
| GLYMA_15G274700 | NA | protein_coding | Glycine max | 15 | 51.289862 | hypothetical protein |
| GLYMA_15G274800 | 1.01E+08 | protein_coding | Glycine max | 15 | 51.291261 | hypothetical protein |
| GLYMA_15G274900 | NA | protein_coding | Glycine max | 15 | 51.30069 | hypothetical protein |
| GLYMA_15G275000 | 1.01E+08 | protein_coding | Glycine max | 15 | 51.338547 | hypothetical protein |
| GLYMA_15G275100 | NA | protein_coding | Glycine max | 15 | 51.350998 | hypothetical protein |
| GLYMA_15G275200 | NA | protein_coding | Glycine max | 15 | 51.364671 | hypothetical protein |
| GLYMA_15G275300 | 1E+08 | protein_coding | Glycine max | 15 | 51.371577 | hypothetical protein |
| GLYMA_15G275400 | 1.01E+08 | protein_coding | Glycine max | 15 | 51.403963 | hypothetical protein |
| GLYMA_15G275500 | 1.01E+08 | protein_coding | Glycine max | 15 | 51.432802 | hypothetical protein |
| GLYMA_16G026700 | 1.01E+08 | protein_coding | Glycine max | 16 | 2.586529 | hypothetical protein |
| GLYMA_16G026800 | NA | protein_coding | Glycine max | 16 | 2.60022 | hypothetical protein |
| GLYMA_16G026900 | 1.01E+08 | protein_coding | Glycine max | 16 | 2.607621 | hypothetical protein |
| GLYMA_16G027000 | NA | protein_coding | Glycine max | 16 | 2.608313 | hypothetical protein |
| GLYMA_16G027100 | 1.01E+08 | protein_coding | Glycine max | 16 | 2.633174 | hypothetical protein |
| GLYMA_16G027200 | 1.01E+08 | protein_coding | Glycine max | 16 | 2.640389 | hypothetical protein |
| GLYMA_16G027300 | 1.01E+08 | protein_coding | Glycine max | 16 | 2.654447 | hypothetical protein |
| GLYMA_16G027400 | 1.01E+08 | protein_coding | Glycine max | 16 | 2.66431 | hypothetical protein |
| GLYMA_16G027500 | 1.01E+08 | protein_coding | Glycine max | 16 | 2.689265 | hypothetical protein |
| GLYMA_16G027600 | 1.01E+08 | protein_coding | Glycine max | 16 | 2.697412 | hypothetical protein |
| GLYMA_16G027700 | NA | protein_coding | Glycine max | 16 | 2.709421 | hypothetical protein |
| GLYMA_16G027800 | NA | protein_coding | Glycine max | 16 | 2.712534 | hypothetical protein |
| GLYMA_16G027900 | NA | protein_coding | Glycine max | 16 | 2.721468 | hypothetical protein |
| GLYMA_16G028000 | NA | protein_coding | Glycine max | 16 | 2.724038 | hypothetical protein |
| GLYMA_16G028100 | 1.01E+08 | protein_coding | Glycine max | 16 | 2.725294 | hypothetical protein |
| GLYMA_16G028200 | NA | protein_coding | Glycine max | 16 | 2.733624 | hypothetical protein |
| GLYMA_16G028300 | NA | protein_coding | Glycine max | 16 | 2.742488 | hypothetical protein |
| GLYMA_16G028400 | NA | protein_coding | Glycine max | 16 | 2.74646 | hypothetical protein |
| GLYMA_16G028500 | 1.01E+08 | protein_coding | Glycine max | 16 | 2.749678 | hypothetical protein |
| GLYMA_16G028600 | NA | protein_coding | Glycine max | 16 | 2.75305 | hypothetical protein |
| GLYMA_16G028700 | 1.01E+08 | protein_coding | Glycine max | 16 | 2.755872 | hypothetical protein |
| GLYMA_16G028800 | NA | protein_coding | Glycine max | 16 | 2.762204 | hypothetical protein |
| GLYMA_16G028900 | NA | protein_coding | Glycine max | 16 | 2.766779 | hypothetical protein |
| GLYMA_16G029000 | NA | protein_coding | Glycine max | 16 | 2.77173 | hypothetical protein |
| GLYMA_16G029100 | 1.01E+08 | protein_coding | Glycine max | 16 | 2.789647 | hypothetical protein |
| GLYMA_16G029200 | 1.03E+08 | protein_coding | Glycine max | 16 | 2.793203 | hypothetical protein |
| GLYMA_16G029300 | 1.01E+08 | protein_coding | Glycine max | 16 | 2.796473 | hypothetical protein |
| GLYMA_16G029400 | 1.01E+08 | protein_coding | Glycine max | 16 | 2.800976 | hypothetical protein |
| GLYMA_16G029500 | 1.01E+08 | protein_coding | Glycine max | 16 | 2.805237 | hypothetical protein |
| GLYMA_16G029600 | 1.01E+08 | protein_coding | Glycine max | 16 | 2.809358 | hypothetical protein |
| GLYMA_16G029700 | NA | protein_coding | Glycine max | 16 | 2.829345 | hypothetical protein |
| GLYMA_16G029800 | NA | protein_coding | Glycine max | 16 | 2.83255 | hypothetical protein |
| GLYMA_16G029900 | 1.01E+08 | protein_coding | Glycine max | 16 | 2.836159 | hypothetical protein |
| GLYMA_16G030000 | NA | protein_coding | Glycine max | 16 | 2.854583 | hypothetical protein |
| GLYMA_16G030100 | 1.01E+08 | protein_coding | Glycine max | 16 | 2.864649 | hypothetical protein |
| GLYMA_16G030200 | 1.01E+08 | protein_coding | Glycine max | 16 | 2.87893 | hypothetical protein |
| GLYMA_16G030300 | 1.01E+08 | protein_coding | Glycine max | 16 | 2.892574 | hypothetical protein |
| GLYMA_16G030400 | NA | protein_coding | Glycine max | 16 | 2.896864 | hypothetical protein |
| GLYMA_16G030500 | NA | protein_coding | Glycine max | 16 | 2.900323 | hypothetical protein |
| GLYMA_16G030600 | 1.01E+08 | protein_coding | Glycine max | 16 | 2.902495 | hypothetical protein |
| GLYMA_16G030700 | 1.01E+08 | protein_coding | Glycine max | 16 | 2.917258 | hypothetical protein |
| GLYMA_16G030800 | NA | protein_coding | Glycine max | 16 | 2.92643 | hypothetical protein |
| GLYMA_16G030900 | NA | protein_coding | Glycine max | 16 | 2.928913 | hypothetical protein |
| GLYMA_16G031000 | NA | protein_coding | Glycine max | 16 | 2.934876 | hypothetical protein |
| GLYMA_16G031100 | 1.01E+08 | protein_coding | Glycine max | 16 | 2.937515 | hypothetical protein |
| GLYMA_16G031200 | NA | protein_coding | Glycine max | 16 | 2.942564 | hypothetical protein |
| GLYMA_16G031300 | 547533 | protein_coding | Glycine max | 16 | 2.949165 | hypothetical protein |
| GLYMA_16G031400 | 1.01E+08 | protein_coding | Glycine max | 16 | 2.951733 | hypothetical protein |
| GLYMA_16G031500 | 1.01E+08 | protein_coding | Glycine max | 16 | 2.956024 | hypothetical protein |
| GLYMA_16G031600 | NA | protein_coding | Glycine max | 16 | 2.964569 | hypothetical protein |
| GLYMA_16G031700 | NA | protein_coding | Glycine max | 16 | 2.990306 | hypothetical protein |
| GLYMA_16G031800 | 1.01E+08 | protein_coding | Glycine max | 16 | 3.017265 | hypothetical protein |
| GLYMA_16G031900 | 1.01E+08 | protein_coding | Glycine max | 16 | 3.020537 | hypothetical protein |
| GLYMA_16G032000 | 1.01E+08 | protein_coding | Glycine max | 16 | 3.031286 | hypothetical protein |
| GLYMA_16G032100 | NA | protein_coding | Glycine max | 16 | 3.036064 | hypothetical protein |
| GLYMA_16G032200 | 1.01E+08 | protein_coding | Glycine max | 16 | 3.050719 | hypothetical protein |
| GLYMA_16G032300 | NA | protein_coding | Glycine max | 16 | 3.062615 | hypothetical protein |
| GLYMA_16G032400 | 1.01E+08 | protein_coding | Glycine max | 16 | 3.064249 | hypothetical protein |
| GLYMA_16G032500 | 1.01E+08 | protein_coding | Glycine max | 16 | 3.072502 | hypothetical protein |
| GLYMA_16G032600 | 778090 | protein_coding | Glycine max | 16 | 3.082737 | hypothetical protein |
| GLYMA_16G037500 | 1.01E+08 | protein_coding | Glycine max | 16 | 3.542455 | hypothetical protein |
| GLYMA_16G037600 | 1.01E+08 | protein_coding | Glycine max | 16 | 3.558978 | hypothetical protein |
| GLYMA_16G037700 | 1.01E+08 | protein_coding | Glycine max | 16 | 3.56735 | hypothetical protein |
| GLYMA_16G037800 | 1.01E+08 | protein_coding | Glycine max | 16 | 3.575465 | hypothetical protein |
| GLYMA_16G037900 | NA | protein_coding | Glycine max | 16 | 3.584655 | hypothetical protein |
| GLYMA_16G038000 | 1.01E+08 | protein_coding | Glycine max | 16 | 3.586151 | hypothetical protein |
| GLYMA_16G038100 | NA | protein_coding | Glycine max | 16 | 3.590425 | hypothetical protein |
| GLYMA_16G038200 | 1.01E+08 | protein_coding | Glycine max | 16 | 3.593536 | hypothetical protein |
| GLYMA_16G038300 | 547643 | protein_coding | Glycine max | 16 | 3.61728 | hypothetical protein |
| GLYMA_16G038400 | 1.01E+08 | protein_coding | Glycine max | 16 | 3.626031 | hypothetical protein |
| GLYMA_16G038500 | 1.01E+08 | protein_coding | Glycine max | 16 | 3.636938 | hypothetical protein |
| GLYMA_16G038600 | NA | protein_coding | Glycine max | 16 | 3.640231 | hypothetical protein |
| GLYMA_16G038700 | NA | protein_coding | Glycine max | 16 | 3.645973 | hypothetical protein |
| GLYMA_16G038800 | 1.01E+08 | protein_coding | Glycine max | 16 | 3.652966 | hypothetical protein |
| GLYMA_16G038900 | 1.01E+08 | protein_coding | Glycine max | 16 | 3.666547 | hypothetical protein |
| GLYMA_16G039000 | NA | protein_coding | Glycine max | 16 | 3.67567 | hypothetical protein |
| GLYMA_16G039100 | NA | protein_coding | Glycine max | 16 | 3.686448 | hypothetical protein |
| GLYMA_16G039200 | NA | protein_coding | Glycine max | 16 | 3.686448 | hypothetical protein |
| GLYMA_16G039300 | NA | protein_coding | Glycine max | 16 | 3.694876 | hypothetical protein |
| GLYMA_16G039400 | 1.01E+08 | protein_coding | Glycine max | 16 | 3.702139 | hypothetical protein |
| GLYMA_16G039500 | 1.01E+08 | protein_coding | Glycine max | 16 | 3.707142 | hypothetical protein |
| GLYMA_16G039600 | 1.01E+08 | protein_coding | Glycine max | 16 | 3.712651 | hypothetical protein |
| GLYMA_16G039700 | 1.01E+08 | protein_coding | Glycine max | 16 | 3.722874 | hypothetical protein |
| GLYMA_16G039800 | 1.01E+08 | protein_coding | Glycine max | 16 | 3.730538 | hypothetical protein |
| GLYMA_16G039900 | 1.01E+08 | protein_coding | Glycine max | 16 | 3.738539 | hypothetical protein |
| GLYMA_16G040000 | 1.01E+08 | protein_coding | Glycine max | 16 | 3.753129 | hypothetical protein |
| GLYMA_16G040100 | 1.01E+08 | protein_coding | Glycine max | 16 | 3.757811 | hypothetical protein |
| GLYMA_16G040200 | 1.01E+08 | protein_coding | Glycine max | 16 | 3.769048 | hypothetical protein |
| GLYMA_16G040300 | 1.01E+08 | protein_coding | Glycine max | 16 | 3.787774 | hypothetical protein |
| GLYMA_16G040400 | NA | protein_coding | Glycine max | 16 | 3.796321 | hypothetical protein |
| GLYMA_16G040500 | 1.01E+08 | protein_coding | Glycine max | 16 | 3.819854 | hypothetical protein |
| GLYMA_16G040600 | NA | protein_coding | Glycine max | 16 | 3.824844 | hypothetical protein |
| GLYMA_16G040700 | NA | protein_coding | Glycine max | 16 | 3.825551 | hypothetical protein |
| GLYMA_16G040800 | NA | protein_coding | Glycine max | 16 | 3.83674 | hypothetical protein |
| GLYMA_16G040900 | 1.01E+08 | protein_coding | Glycine max | 16 | 3.838642 | hypothetical protein |
| GLYMA_16G041000 | NA | protein_coding | Glycine max | 16 | 3.852109 | hypothetical protein |
| GLYMA_16G041100 | NA | protein_coding | Glycine max | 16 | 3.856191 | hypothetical protein |
| GLYMA_16G041200 | 547687 | protein_coding | Glycine max | 16 | 3.861427 | hypothetical protein |
| GLYMA_16G041300 | NA | protein_coding | Glycine max | 16 | 3.873566 | hypothetical protein |
| GLYMA_16G041400 | 1.01E+08 | protein_coding | Glycine max | 16 | 3.879903 | hypothetical protein |
| GLYMA_16G041500 | 1.01E+08 | protein_coding | Glycine max | 16 | 3.888459 | hypothetical protein |
| GLYMA_16G041600 | 1.01E+08 | protein_coding | Glycine max | 16 | 3.904131 | hypothetical protein |
| GLYMA_16G041700 | 1.01E+08 | protein_coding | Glycine max | 16 | 3.909907 | hypothetical protein |
| GLYMA_16G041800 | 1.01E+08 | protein_coding | Glycine max | 16 | 3.914214 | hypothetical protein |
| GLYMA_16G041900 | NA | protein_coding | Glycine max | 16 | 3.920997 | hypothetical protein |
| GLYMA_16G042000 | 1.01E+08 | protein_coding | Glycine max | 16 | 3.925957 | hypothetical protein |
| GLYMA_16G042100 | NA | protein_coding | Glycine max | 16 | 3.93167 | hypothetical protein |
| GLYMA_16G042200 | 1.01E+08 | protein_coding | Glycine max | 16 | 3.933842 | hypothetical protein |
| GLYMA_16G042300 | 1.01E+08 | protein_coding | Glycine max | 16 | 3.947147 | hypothetical protein |
| GLYMA_16G042400 | NA | protein_coding | Glycine max | 16 | 3.948915 | hypothetical protein |
| GLYMA_16G042500 | 1.01E+08 | protein_coding | Glycine max | 16 | 3.956698 | hypothetical protein |
| GLYMA_16G042600 | 1.01E+08 | protein_coding | Glycine max | 16 | 3.964264 | hypothetical protein |
| GLYMA_16G042700 | 1.01E+08 | protein_coding | Glycine max | 16 | 3.982381 | hypothetical protein |
| GLYMA_16G042800 | 1.01E+08 | protein_coding | Glycine max | 16 | 3.991872 | hypothetical protein |
| GLYMA_16G042900 | 1.01E+08 | protein_coding | Glycine max | 16 | 4.011699 | hypothetical protein |
| GLYMA_16G043000 | 1.01E+08 | protein_coding | Glycine max | 16 | 4.02494 | hypothetical protein |
| GLYMA_16G043100 | 1.01E+08 | protein_coding | Glycine max | 16 | 4.02898 | hypothetical protein |
| GLYMA_16G043200 | NA | protein_coding | Glycine max | 16 | 4.037637 | hypothetical protein |
| GLYMA_16G076700 | 1.01E+08 | protein_coding | Glycine max | 16 | 7.832417 | hypothetical protein |
| GLYMA_16G076800 | 1.01E+08 | protein_coding | Glycine max | 16 | 7.84605 | hypothetical protein |
| GLYMA_16G076900 | 1.01E+08 | protein_coding | Glycine max | 16 | 7.862192 | hypothetical protein |
| GLYMA_16G077000 | NA | protein_coding | Glycine max | 16 | 7.88644 | hypothetical protein |
| GLYMA_16G077100 | 1.01E+08 | protein_coding | Glycine max | 16 | 7.902269 | hypothetical protein |
| GLYMA_16G077200 | 1.01E+08 | protein_coding | Glycine max | 16 | 7.914345 | hypothetical protein |
| GLYMA_16G077300 | 1.01E+08 | protein_coding | Glycine max | 16 | 7.923562 | hypothetical protein |
| GLYMA_16G077400 | 1.01E+08 | protein_coding | Glycine max | 16 | 7.949438 | hypothetical protein |
| GLYMA_16G077500 | NA | protein_coding | Glycine max | 16 | 7.959904 | hypothetical protein |
| GLYMA_16G077600 | 1E+08 | protein_coding | Glycine max | 16 | 7.985004 | hypothetical protein |
| GLYMA_16G077700 | NA | protein_coding | Glycine max | 16 | 8.001027 | hypothetical protein |
| GLYMA_16G077800 | 1.01E+08 | protein_coding | Glycine max | 16 | 8.017406 | hypothetical protein |
| GLYMA_16G077900 | 1.03E+08 | protein_coding | Glycine max | 16 | 8.023101 | hypothetical protein |
| GLYMA_16G078000 | NA | protein_coding | Glycine max | 16 | 8.034739 | hypothetical protein |
| GLYMA_16G078100 | 1.01E+08 | protein_coding | Glycine max | 16 | 8.03974 | hypothetical protein |
| GLYMA_16G078200 | 1.01E+08 | protein_coding | Glycine max | 16 | 8.060944 | hypothetical protein |
| GLYMA_16G078300 | 1.01E+08 | protein_coding | Glycine max | 16 | 8.071647 | hypothetical protein |
| GLYMA_16G078400 | 1.01E+08 | protein_coding | Glycine max | 16 | 8.075263 | hypothetical protein |
| GLYMA_16G078500 | 1.01E+08 | protein_coding | Glycine max | 16 | 8.084011 | hypothetical protein |
| GLYMA_16G078600 | NA | protein_coding | Glycine max | 16 | 8.087561 | hypothetical protein |
| GLYMA_16G078700 | NA | protein_coding | Glycine max | 16 | 8.088302 | hypothetical protein |
| GLYMA_16G078800 | 1.01E+08 | protein_coding | Glycine max | 16 | 8.103196 | hypothetical protein |
| GLYMA_16G078900 | 1.01E+08 | protein_coding | Glycine max | 16 | 8.118602 | hypothetical protein |
| GLYMA_16G079000 | NA | protein_coding | Glycine max | 16 | 8.126768 | hypothetical protein |
| GLYMA_16G079100 | 1.01E+08 | protein_coding | Glycine max | 16 | 8.135056 | hypothetical protein |
| GLYMA_16G079200 | 1E+08 | protein_coding | Glycine max | 16 | 8.18366 | hypothetical protein |
| GLYMA_16G079300 | NA | protein_coding | Glycine max | 16 | 8.187731 | hypothetical protein |
| GLYMA_16G079400 | NA | protein_coding | Glycine max | 16 | 8.196845 | hypothetical protein |
| GLYMA_16G079500 | NA | protein_coding | Glycine max | 16 | 8.26359 | hypothetical protein |
| GLYMA_16G079600 | 1.01E+08 | protein_coding | Glycine max | 16 | 8.280664 | hypothetical protein |
| GLYMA_17G097200 | NA | protein_coding | Glycine max | 17 | 7.660368 | hypothetical protein |
| GLYMA_17G097400 | NA | protein_coding | Glycine max | 17 | 7.664108 | hypothetical protein |
| GLYMA_17G097500 | 1.01E+08 | protein_coding | Glycine max | 17 | 7.667059 | hypothetical protein |
| GLYMA_17G097600 | 1.01E+08 | protein_coding | Glycine max | 17 | 7.676548 | hypothetical protein |
| GLYMA_17G097700 | NA | protein_coding | Glycine max | 17 | 7.685599 | hypothetical protein |
| GLYMA_17G097800 | 1.01E+08 | protein_coding | Glycine max | 17 | 7.69184 | hypothetical protein |
| GLYMA_17G097900 | 1.01E+08 | protein_coding | Glycine max | 17 | 7.706604 | hypothetical protein |
| GLYMA_17G098000 | NA | protein_coding | Glycine max | 17 | 7.720344 | hypothetical protein |
| GLYMA_17G098100 | NA | protein_coding | Glycine max | 17 | 7.726116 | hypothetical protein |
| GLYMA_17G098200 | 1.01E+08 | protein_coding | Glycine max | 17 | 7.732994 | hypothetical protein |
| GLYMA_17G098300 | 1.01E+08 | protein_coding | Glycine max | 17 | 7.744245 | hypothetical protein |
| GLYMA_17G098400 | 1.01E+08 | protein_coding | Glycine max | 17 | 7.756424 | hypothetical protein |
| GLYMA_17G098500 | 1.01E+08 | protein_coding | Glycine max | 17 | 7.765666 | hypothetical protein |
| GLYMA_17G098600 | 1.01E+08 | protein_coding | Glycine max | 17 | 7.779339 | hypothetical protein |
| GLYMA_17G098700 | 1.01E+08 | protein_coding | Glycine max | 17 | 7.787376 | hypothetical protein |
| GLYMA_17G098800 | 1.01E+08 | protein_coding | Glycine max | 17 | 7.796575 | hypothetical protein |
| GLYMA_17G098900 | 1.01E+08 | protein_coding | Glycine max | 17 | 7.800159 | hypothetical protein |
| GLYMA_17G099000 | NA | protein_coding | Glycine max | 17 | 7.805371 | hypothetical protein |
| GLYMA_17G099100 | 1.01E+08 | protein_coding | Glycine max | 17 | 7.81194 | hypothetical protein |
| GLYMA_17G099200 | 1.01E+08 | protein_coding | Glycine max | 17 | 7.832052 | hypothetical protein |
| GLYMA_17G099300 | 1.01E+08 | protein_coding | Glycine max | 17 | 7.838198 | hypothetical protein |
| GLYMA_17G099400 | 1.01E+08 | protein_coding | Glycine max | 17 | 7.841906 | hypothetical protein |
| GLYMA_17G099500 | NA | protein_coding | Glycine max | 17 | 7.846494 | hypothetical protein |
| GLYMA_17G099600 | NA | protein_coding | Glycine max | 17 | 7.850694 | hypothetical protein |
| GLYMA_17G099700 | NA | protein_coding | Glycine max | 17 | 7.85628 | hypothetical protein |
| GLYMA_17G099800 | 1.01E+08 | protein_coding | Glycine max | 17 | 7.861702 | hypothetical protein |
| GLYMA_17G099900 | NA | protein_coding | Glycine max | 17 | 7.87883 | hypothetical protein |
| GLYMA_17G100000 | NA | protein_coding | Glycine max | 17 | 7.88456 | hypothetical protein |
| GLYMA_17G100100 | 1.01E+08 | protein_coding | Glycine max | 17 | 7.899983 | hypothetical protein |
| GLYMA_17G100200 | NA | protein_coding | Glycine max | 17 | 7.902958 | hypothetical protein |
| GLYMA_17G100300 | 1.01E+08 | protein_coding | Glycine max | 17 | 7.907773 | hypothetical protein |
| GLYMA_17G100400 | 1.01E+08 | protein_coding | Glycine max | 17 | 7.911866 | hypothetical protein |
| GLYMA_17G100500 | NA | protein_coding | Glycine max | 17 | 7.914881 | hypothetical protein |
| GLYMA_17G100600 | 1.01E+08 | protein_coding | Glycine max | 17 | 7.917204 | hypothetical protein |
| GLYMA_17G100700 | 1.01E+08 | protein_coding | Glycine max | 17 | 7.921514 | hypothetical protein |
| GLYMA_17G100800 | 1.01E+08 | protein_coding | Glycine max | 17 | 7.92619 | hypothetical protein |
| GLYMA_17G100900 | 1.01E+08 | protein_coding | Glycine max | 17 | 7.930378 | hypothetical protein |
| GLYMA_17G101000 | NA | protein_coding | Glycine max | 17 | 7.93795 | hypothetical protein |
| GLYMA_17G101100 | NA | protein_coding | Glycine max | 17 | 7.94515 | hypothetical protein |
| GLYMA_17G101200 | NA | protein_coding | Glycine max | 17 | 7.946441 | hypothetical protein |
| GLYMA_17G101300 | NA | protein_coding | Glycine max | 17 | 7.951772 | hypothetical protein |
| GLYMA_17G101400 | 1.01E+08 | protein_coding | Glycine max | 17 | 7.956697 | hypothetical protein |
| GLYMA_17G101500 | 1E+08 | protein_coding | Glycine max | 17 | 7.978357 | hypothetical protein |
| GLYMA_17G101600 | NA | protein_coding | Glycine max | 17 | 7.987483 | hypothetical protein |
| GLYMA_17G101700 | 1.01E+08 | protein_coding | Glycine max | 17 | 7.99329 | hypothetical protein |
| GLYMA_17G101800 | 1E+08 | protein_coding | Glycine max | 17 | 7.998166 | hypothetical protein |
| GLYMA_17G101900 | NA | protein_coding | Glycine max | 17 | 8.004316 | hypothetical protein |
| GLYMA_17G102000 | 1.01E+08 | protein_coding | Glycine max | 17 | 8.007473 | hypothetical protein |
| GLYMA_17G102100 | 1.01E+08 | protein_coding | Glycine max | 17 | 8.01319 | hypothetical protein |
| GLYMA_17G102200 | 1.01E+08 | protein_coding | Glycine max | 17 | 8.01819 | hypothetical protein |
| GLYMA_17G102300 | 1.01E+08 | protein_coding | Glycine max | 17 | 8.024107 | hypothetical protein |
| GLYMA_17G102400 | 1.01E+08 | protein_coding | Glycine max | 17 | 8.027482 | hypothetical protein |
| GLYMA_17G102500 | 1.03E+08 | protein_coding | Glycine max | 17 | 8.030863 | hypothetical protein |
| GLYMA_17G102600 | 1.01E+08 | protein_coding | Glycine max | 17 | 8.034007 | hypothetical protein |
| GLYMA_17G102700 | 1.01E+08 | protein_coding | Glycine max | 17 | 8.041215 | hypothetical protein |
| GLYMA_17G102800 | NA | protein_coding | Glycine max | 17 | 8.045209 | hypothetical protein |
| GLYMA_17G102900 | 1.01E+08 | protein_coding | Glycine max | 17 | 8.07498 | hypothetical protein |
| GLYMA_17G103000 | NA | protein_coding | Glycine max | 17 | 8.088583 | hypothetical protein |
| GLYMA_17G103100 | 1E+08 | protein_coding | Glycine max | 17 | 8.093285 | hypothetical protein |
| GLYMA_17G103200 | NA | protein_coding | Glycine max | 17 | 8.096158 | hypothetical protein |
| GLYMA_17G103300 | 1.03E+08 | protein_coding | Glycine max | 17 | 8.102348 | hypothetical protein |
| GLYMA_17G103400 | 1.01E+08 | protein_coding | Glycine max | 17 | 8.118176 | hypothetical protein |
| GLYMA_17G103500 | 1.01E+08 | protein_coding | Glycine max | 17 | 8.127907 | hypothetical protein |
| GLYMA_17G103600 | NA | protein_coding | Glycine max | 17 | 8.13058 | hypothetical protein |
| GLYMA_17G103700 | 1.01E+08 | protein_coding | Glycine max | 17 | 8.138957 | hypothetical protein |
| GLYMA_17G103800 | NA | protein_coding | Glycine max | 17 | 8.145246 | hypothetical protein |
| GLYMA_17G103900 | 1.01E+08 | protein_coding | Glycine max | 17 | 8.149107 | hypothetical protein |
| GLYMA_17G104000 | 1.01E+08 | protein_coding | Glycine max | 17 | 8.153582 | hypothetical protein |
| GLYMA_17G104100 | 1.01E+08 | protein_coding | Glycine max | 17 | 8.160933 | hypothetical protein |
| GLYMA_18G178400 | NA | protein_coding | Glycine max | 18 | 42.730833 | hypothetical protein |
| GLYMA_18G178500 | 1.01E+08 | protein_coding | Glycine max | 18 | 42.787936 | hypothetical protein |
| GLYMA_18G178600 | NA | protein_coding | Glycine max | 18 | 42.791603 | hypothetical protein |
| GLYMA_18G178700 | NA | protein_coding | Glycine max | 18 | 42.837284 | hypothetical protein |
| GLYMA_18G178800 | NA | protein_coding | Glycine max | 18 | 42.918477 | hypothetical protein |
| GLYMA_18G178900 | NA | protein_coding | Glycine max | 18 | 43.114604 | hypothetical protein |
| GLYMA_18G179000 | NA | protein_coding | Glycine max | 18 | 43.119526 | hypothetical protein |
| GLYMA_18G179100 | NA | protein_coding | Glycine max | 18 | 43.184175 | hypothetical protein |
| GLYMA_18G298000 | 1.01E+08 | protein_coding | Glycine max | 18 | 57.564138 | hypothetical protein |
| GLYMA_18G298100 | 1.01E+08 | protein_coding | Glycine max | 18 | 57.571765 | hypothetical protein |
| GLYMA_18G298200 | NA | protein_coding | Glycine max | 18 | 57.579777 | hypothetical protein |
| GLYMA_18G298300 | 547889 | protein_coding | Glycine max | 18 | 57.587135 | hypothetical protein |
| GLYMA_18G298400 | NA | protein_coding | Glycine max | 18 | 57.591051 | hypothetical protein |
| GLYMA_18G298500 | 1.01E+08 | protein_coding | Glycine max | 18 | 57.596668 | hypothetical protein |
| GLYMA_18G298600 | 1.01E+08 | protein_coding | Glycine max | 18 | 57.614292 | hypothetical protein |
| GLYMA_18G298700 | 1.01E+08 | protein_coding | Glycine max | 18 | 57.619193 | hypothetical protein |
| GLYMA_18G298800 | NA | protein_coding | Glycine max | 18 | 57.633577 | hypothetical protein |
| GLYMA_18G298900 | 1.01E+08 | protein_coding | Glycine max | 18 | 57.653717 | hypothetical protein |
| GLYMA_18G299000 | 1.01E+08 | protein_coding | Glycine max | 18 | 57.670462 | hypothetical protein |
| GLYMA_18G299100 | 1.01E+08 | protein_coding | Glycine max | 18 | 57.674222 | hypothetical protein |
| GLYMA_18G299200 | NA | protein_coding | Glycine max | 18 | 57.679867 | hypothetical protein |
| GLYMA_18G299300 | 1.01E+08 | protein_coding | Glycine max | 18 | 57.705112 | hypothetical protein |
| GLYMA_18G299400 | 1.01E+08 | protein_coding | Glycine max | 18 | 57.718284 | hypothetical protein |
| GLYMA_18G299500 | 1.01E+08 | protein_coding | Glycine max | 18 | 57.741567 | hypothetical protein |
| GLYMA_18G299600 | NA | protein_coding | Glycine max | 18 | 57.751224 | hypothetical protein |
| GLYMA_18G299700 | NA | protein_coding | Glycine max | 18 | 57.755837 | hypothetical protein |
| GLYMA_18G299800 | 1.01E+08 | protein_coding | Glycine max | 18 | 57.757422 | hypothetical protein |
| GLYMA_18G299900 | 1.01E+08 | protein_coding | Glycine max | 18 | 57.763913 | hypothetical protein |
| GLYMA_18G300000 | NA | protein_coding | Glycine max | 18 | 57.771055 | hypothetical protein |
| GLYMA_18G300100 | 1.01E+08 | protein_coding | Glycine max | 18 | 57.774232 | hypothetical protein |
| GLYMA_18G300200 | 1.01E+08 | protein_coding | Glycine max | 18 | 57.789188 | hypothetical protein |
| GLYMA_18G300300 | 1.01E+08 | protein_coding | Glycine max | 18 | 57.823394 | hypothetical protein |
| GLYMA_18G300400 | 1.01E+08 | protein_coding | Glycine max | 18 | 57.831381 | hypothetical protein |
| GLYMA_18G300500 | 1.01E+08 | protein_coding | Glycine max | 18 | 57.841316 | hypothetical protein |
| GLYMA_18G300600 | NA | protein_coding | Glycine max | 18 | 57.844897 | hypothetical protein |
| GLYMA_18G300700 | 1.01E+08 | protein_coding | Glycine max | 18 | 57.852461 | hypothetical protein |
| GLYMA_18G300800 | 1.01E+08 | protein_coding | Glycine max | 18 | 57.860162 | hypothetical protein |
| GLYMA_18G300900 | 1.01E+08 | protein_coding | Glycine max | 18 | 57.867768 | hypothetical protein |
| GLYMA_18G301000 | NA | protein_coding | Glycine max | 18 | 57.890959 | hypothetical protein |
| GLYMA_18G301100 | 1.01E+08 | protein_coding | Glycine max | 18 | 57.894037 | hypothetical protein |
| GLYMA_18G301200 | 1.01E+08 | protein_coding | Glycine max | 18 | 57.903571 | hypothetical protein |
| GLYMA_18G301300 | NA | protein_coding | Glycine max | 18 | 57.913464 | hypothetical protein |
| GLYMA_18G301400 | NA | protein_coding | Glycine max | 18 | 57.915964 | hypothetical protein |
| GLYMA_18G301500 | 1E+08 | protein_coding | Glycine max | 18 | 57.926526 | hypothetical protein |
| GLYMA_18G301600 | 1.01E+08 | protein_coding | Glycine max | 18 | 57.939468 | hypothetical protein |
| GLYMA_18G301700 | 1.01E+08 | protein_coding | Glycine max | 18 | 57.945537 | hypothetical protein |
| GLYMA_18G301800 | NA | protein_coding | Glycine max | 18 | 57.950394 | hypothetical protein |
| GLYMA_18G301900 | 1E+08 | protein_coding | Glycine max | 18 | 57.953323 | hypothetical protein |
| GLYMA_18G302000 | 1.01E+08 | protein_coding | Glycine max | 18 | 57.965322 | hypothetical protein |
| GLYMA_18G302100 | NA | protein_coding | Glycine max | 18 | 57.988925 | hypothetical protein |
| GLYMA_18G302200 | NA | protein_coding | Glycine max | 18 | 57.998307 | hypothetical protein |
| GLYMA_18G302300 | NA | protein_coding | Glycine max | 18 | 58.014611 | hypothetical protein |
| GLYMA_19G014200 | 1.01E+08 | protein_coding | Glycine max | 19 | 1.345795 | hypothetical protein |
| GLYMA_19G014300 | 1.01E+08 | protein_coding | Glycine max | 19 | 1.35088 | hypothetical protein |
| GLYMA_19G014400 | 1.01E+08 | protein_coding | Glycine max | 19 | 1.364758 | hypothetical protein |
| GLYMA_19G014500 | 1.01E+08 | protein_coding | Glycine max | 19 | 1.379893 | hypothetical protein |
| GLYMA_19G014600 | 1.01E+08 | protein_coding | Glycine max | 19 | 1.396438 | hypothetical protein |
| GLYMA_19G014700 | 1.01E+08 | protein_coding | Glycine max | 19 | 1.401805 | hypothetical protein |
| GLYMA_19G014800 | NA | protein_coding | Glycine max | 19 | 1.422001 | hypothetical protein |
| GLYMA_19G014900 | NA | protein_coding | Glycine max | 19 | 1.432539 | hypothetical protein |
| GLYMA_19G015000 | 1.01E+08 | protein_coding | Glycine max | 19 | 1.451328 | hypothetical protein |
| GLYMA_19G015100 | NA | protein_coding | Glycine max | 19 | 1.461875 | hypothetical protein |
| GLYMA_19G015200 | NA | protein_coding | Glycine max | 19 | 1.463404 | hypothetical protein |
| GLYMA_19G015300 | 1.01E+08 | protein_coding | Glycine max | 19 | 1.500237 | hypothetical protein |
| GLYMA_19G015400 | NA | protein_coding | Glycine max | 19 | 1.519655 | hypothetical protein |
| GLYMA_19G015500 | 1E+08 | protein_coding | Glycine max | 19 | 1.53227 | hypothetical protein |
| GLYMA_19G015600 | NA | protein_coding | Glycine max | 19 | 1.536852 | hypothetical protein |
| GLYMA_19G015700 | NA | protein_coding | Glycine max | 19 | 1.539784 | hypothetical protein |
| GLYMA_19G015800 | 1.01E+08 | protein_coding | Glycine max | 19 | 1.543903 | hypothetical protein |
| GLYMA_19G015900 | 1.01E+08 | protein_coding | Glycine max | 19 | 1.570574 | hypothetical protein |
| GLYMA_19G016000 | 1.01E+08 | protein_coding | Glycine max | 19 | 1.585285 | hypothetical protein |
| GLYMA_19G016100 | 1.01E+08 | protein_coding | Glycine max | 19 | 1.592368 | hypothetical protein |
| GLYMA_19G016200 | NA | protein_coding | Glycine max | 19 | 1.607233 | hypothetical protein |
| GLYMA_19G016300 | 1.01E+08 | protein_coding | Glycine max | 19 | 1.611333 | hypothetical protein |
| GLYMA_19G016400 | NA | protein_coding | Glycine max | 19 | 1.631472 | hypothetical protein |
| GLYMA_19G016500 | NA | protein_coding | Glycine max | 19 | 1.682629 | hypothetical protein |
| GLYMA_19G016600 | 1.01E+08 | protein_coding | Glycine max | 19 | 1.691184 | hypothetical protein |
| GLYMA_19G016700 | 1.01E+08 | protein_coding | Glycine max | 19 | 1.728554 | hypothetical protein |
| GLYMA_19G016800 | 1.01E+08 | protein_coding | Glycine max | 19 | 1.745225 | hypothetical protein |
| GLYMA_19G016900 | NA | protein_coding | Glycine max | 19 | 1.75084 | hypothetical protein |
| GLYMA_19G017000 | NA | protein_coding | Glycine max | 19 | 1.759321 | hypothetical protein |
| GLYMA_19G017100 | 1.01E+08 | protein_coding | Glycine max | 19 | 1.773502 | hypothetical protein |
| GLYMA_19G017200 | 1.01E+08 | protein_coding | Glycine max | 19 | 1.785304 | hypothetical protein |
| GLYMA_19G017300 | NA | protein_coding | Glycine max | 19 | 1.814297 | hypothetical protein |
| GLYMA_19G017400 | 1.03E+08 | protein_coding | Glycine max | 19 | 1.819967 | hypothetical protein |
| GLYMA_19G017500 | NA | protein_coding | Glycine max | 19 | 1.827038 | hypothetical protein |
| GLYMA_19G017600 | NA | protein_coding | Glycine max | 19 | 1.832611 | hypothetical protein |
| GLYMA_19G017700 | 1.01E+08 | protein_coding | Glycine max | 19 | 1.838597 | hypothetical protein |
| GLYMA_19G017800 | NA | protein_coding | Glycine max | 19 | 1.844347 | hypothetical protein |
| GLYMA_19G130800 | NA | protein_coding | Glycine max | 19 | 39.069648 | hypothetical protein |
| GLYMA_19G130900 | NA | protein_coding | Glycine max | 19 | 39.114144 | hypothetical protein |
| GLYMA_19G131000 | NA | protein_coding | Glycine max | 19 | 39.159608 | hypothetical protein |
| GLYMA_19G131100 | NA | protein_coding | Glycine max | 19 | 39.173833 | hypothetical protein |
| GLYMA_19G131200 | NA | protein_coding | Glycine max | 19 | 39.176659 | hypothetical protein |
| GLYMA_19G131300 | 1.01E+08 | protein_coding | Glycine max | 19 | 39.186985 | hypothetical protein |
| GLYMA_19G131400 | 1.01E+08 | protein_coding | Glycine max | 19 | 39.195741 | hypothetical protein |
| GLYMA_19G131500 | NA | protein_coding | Glycine max | 19 | 39.199359 | hypothetical protein |
| GLYMA_19G131600 | 1E+08 | protein_coding | Glycine max | 19 | 39.215558 | hypothetical protein |
| GLYMA_19G131700 | NA | protein_coding | Glycine max | 19 | 39.220443 | hypothetical protein |
| GLYMA_19G131800 | 1.01E+08 | protein_coding | Glycine max | 19 | 39.243409 | hypothetical protein |
| GLYMA_19G131900 | NA | protein_coding | Glycine max | 19 | 39.258849 | hypothetical protein |
| GLYMA_19G132000 | 1.01E+08 | protein_coding | Glycine max | 19 | 39.262821 | hypothetical protein |
| GLYMA_19G132100 | NA | protein_coding | Glycine max | 19 | 39.272381 | hypothetical protein |
| GLYMA_19G132200 | 1.01E+08 | protein_coding | Glycine max | 19 | 39.280021 | hypothetical protein |
| GLYMA_19G132300 | 1.01E+08 | protein_coding | Glycine max | 19 | 39.29354 | hypothetical protein |
| GLYMA_19G132400 | 1.01E+08 | protein_coding | Glycine max | 19 | 39.328218 | hypothetical protein |
| GLYMA_19G132500 | NA | protein_coding | Glycine max | 19 | 39.349172 | hypothetical protein |
| GLYMA_19G132600 | NA | protein_coding | Glycine max | 19 | 39.355654 | hypothetical protein |
| GLYMA_19G132700 | NA | protein_coding | Glycine max | 19 | 39.375803 | hypothetical protein |
| GLYMA_19G132800 | 1.01E+08 | protein_coding | Glycine max | 19 | 39.397758 | hypothetical protein |
| GLYMA_19G132900 | 1.01E+08 | protein_coding | Glycine max | 19 | 39.41955 | hypothetical protein |
| GLYMA_19G133000 | 1.01E+08 | protein_coding | Glycine max | 19 | 39.429765 | hypothetical protein |
| GLYMA_19G133100 | 1.01E+08 | protein_coding | Glycine max | 19 | 39.435623 | hypothetical protein |
| GLYMA_19G133200 | NA | protein_coding | Glycine max | 19 | 39.44274 | hypothetical protein |
| GLYMA_19G133300 | 1.01E+08 | protein_coding | Glycine max | 19 | 39.448889 | hypothetical protein |
| GLYMA_19G133400 | 1.01E+08 | protein_coding | Glycine max | 19 | 39.452516 | hypothetical protein |
| GLYMA_19G133500 | 1.01E+08 | protein_coding | Glycine max | 19 | 39.464716 | hypothetical protein |
| GLYMA_19G133600 | 1.01E+08 | protein_coding | Glycine max | 19 | 39.470881 | hypothetical protein |
| GLYMA_19G133700 | NA | protein_coding | Glycine max | 19 | 39.477915 | hypothetical protein |
| GLYMA_19G133800 | 1E+08 | protein_coding | Glycine max | 19 | 39.488115 | hypothetical protein |
| GLYMA_19G133900 | NA | protein_coding | Glycine max | 19 | 39.490822 | hypothetical protein |
| GLYMA_19G134000 | 1.01E+08 | protein_coding | Glycine max | 19 | 39.497308 | hypothetical protein |
| GLYMA_19G134100 | 1.01E+08 | protein_coding | Glycine max | 19 | 39.510186 | hypothetical protein |
| GLYMA_19G134200 | NA | protein_coding | Glycine max | 19 | 39.523361 | hypothetical protein |
| GLYMA_19G134300 | 1.01E+08 | protein_coding | Glycine max | 19 | 39.537942 | hypothetical protein |
| GLYMA_19G134400 | NA | protein_coding | Glycine max | 19 | 39.551222 | hypothetical protein |
| GLYMA_19G134500 | NA | protein_coding | Glycine max | 19 | 39.551479 | hypothetical protein |
| GLYMA_19G134600 | 1.01E+08 | protein_coding | Glycine max | 19 | 39.566372 | hypothetical protein |
| GLYMA_19G134700 | 1.01E+08 | protein_coding | Glycine max | 19 | 39.576172 | hypothetical protein |
| GLYMA_19G159200 | 1.01E+08 | protein_coding | Glycine max | 19 | 42.004441 | hypothetical protein |
| GLYMA_19G159300 | 1.01E+08 | protein_coding | Glycine max | 19 | 42.00906 | hypothetical protein |
| GLYMA_19G159400 | 1.01E+08 | protein_coding | Glycine max | 19 | 42.015735 | hypothetical protein |
| GLYMA_19G159500 | NA | protein_coding | Glycine max | 19 | 42.029589 | hypothetical protein |
| GLYMA_19G159600 | 1.01E+08 | protein_coding | Glycine max | 19 | 42.044556 | hypothetical protein |
| GLYMA_19G159700 | 1.01E+08 | protein_coding | Glycine max | 19 | 42.064879 | hypothetical protein |
| GLYMA_19G159800 | NA | protein_coding | Glycine max | 19 | 42.073943 | hypothetical protein |
| GLYMA_19G159900 | 1.01E+08 | protein_coding | Glycine max | 19 | 42.08234 | hypothetical protein |
| GLYMA_19G160000 | 1.01E+08 | protein_coding | Glycine max | 19 | 42.097454 | hypothetical protein |
| GLYMA_19G160100 | 1.01E+08 | protein_coding | Glycine max | 19 | 42.103251 | hypothetical protein |
| GLYMA_19G160200 | 1.01E+08 | protein_coding | Glycine max | 19 | 42.107601 | hypothetical protein |
| GLYMA_19G160300 | 1.03E+08 | protein_coding | Glycine max | 19 | 42.120169 | hypothetical protein |
| GLYMA_19G160400 | 1.01E+08 | protein_coding | Glycine max | 19 | 42.123579 | hypothetical protein |
| GLYMA_19G160500 | 1E+08 | protein_coding | Glycine max | 19 | 42.12536 | hypothetical protein |
| GLYMA_19G160600 | 1.01E+08 | protein_coding | Glycine max | 19 | 42.132305 | hypothetical protein |
| GLYMA_19G160700 | 1.01E+08 | protein_coding | Glycine max | 19 | 42.138179 | hypothetical protein |
| GLYMA_19G160800 | 732610 | protein_coding | Glycine max | 19 | 42.148525 | hypothetical protein |
| GLYMA_19G160900 | 1.01E+08 | protein_coding | Glycine max | 19 | 42.160254 | hypothetical protein |
| GLYMA_19G161000 | 1.01E+08 | protein_coding | Glycine max | 19 | 42.172235 | hypothetical protein |
| GLYMA_19G161100 | NA | protein_coding | Glycine max | 19 | 42.187796 | hypothetical protein |
| GLYMA_19G161200 | 1.01E+08 | protein_coding | Glycine max | 19 | 42.19644 | hypothetical protein |
| GLYMA_19G161300 | 1.01E+08 | protein_coding | Glycine max | 19 | 42.202458 | hypothetical protein |
| GLYMA_19G161400 | 1E+08 | protein_coding | Glycine max | 19 | 42.212766 | hypothetical protein |
| GLYMA_19G161500 | 1.01E+08 | protein_coding | Glycine max | 19 | 42.215154 | hypothetical protein |
| GLYMA_19G161600 | 1.01E+08 | protein_coding | Glycine max | 19 | 42.231749 | hypothetical protein |
| GLYMA_19G161700 | 1.01E+08 | protein_coding | Glycine max | 19 | 42.238327 | hypothetical protein |
| GLYMA_19G161800 | 1.01E+08 | protein_coding | Glycine max | 19 | 42.249023 | hypothetical protein |
| GLYMA_19G161900 | NA | protein_coding | Glycine max | 19 | 42.254412 | hypothetical protein |
| GLYMA_19G162000 | 1.01E+08 | protein_coding | Glycine max | 19 | 42.270422 | hypothetical protein |
| GLYMA_19G162100 | 1.01E+08 | protein_coding | Glycine max | 19 | 42.275657 | hypothetical protein |
| GLYMA_19G162200 | NA | protein_coding | Glycine max | 19 | 42.296133 | hypothetical protein |
| GLYMA_19G162300 | NA | protein_coding | Glycine max | 19 | 42.306008 | hypothetical protein |
| GLYMA_19G162400 | 1.01E+08 | protein_coding | Glycine max | 19 | 42.319039 | hypothetical protein |
| GLYMA_19G162500 | 1.03E+08 | protein_coding | Glycine max | 19 | 42.327858 | hypothetical protein |
| GLYMA_19G162600 | 1.03E+08 | protein_coding | Glycine max | 19 | 42.337724 | hypothetical protein |
| GLYMA_19G162700 | NA | protein_coding | Glycine max | 19 | 42.345366 | hypothetical protein |
| GLYMA_19G162800 | 1.01E+08 | protein_coding | Glycine max | 19 | 42.347789 | hypothetical protein |
| GLYMA_19G162900 | 1.01E+08 | protein_coding | Glycine max | 19 | 42.36353 | hypothetical protein |
| GLYMA_19G163000 | NA | protein_coding | Glycine max | 19 | 42.374752 | hypothetical protein |
| GLYMA_19G163100 | NA | protein_coding | Glycine max | 19 | 42.393385 | hypothetical protein |
| GLYMA_19G163200 | 1.01E+08 | protein_coding | Glycine max | 19 | 42.399105 | hypothetical protein |
| GLYMA_19G163300 | 1.01E+08 | protein_coding | Glycine max | 19 | 42.403172 | hypothetical protein |
| GLYMA_19G163400 | NA | protein_coding | Glycine max | 19 | 42.432842 | hypothetical protein |
| GLYMA_19G163500 | 732654 | protein_coding | Glycine max | 19 | 42.435811 | hypothetical protein |
| GLYMA_19G163600 | 1.01E+08 | protein_coding | Glycine max | 19 | 42.444145 | hypothetical protein |
| GLYMA_19G163700 | 1.01E+08 | protein_coding | Glycine max | 19 | 42.450312 | hypothetical protein |
| GLYMA_19G163800 | NA | protein_coding | Glycine max | 19 | 42.456513 | hypothetical protein |
| GLYMA_19G163900 | NA | protein_coding | Glycine max | 19 | 42.463385 | hypothetical protein |
| GLYMA_19G164000 | NA | protein_coding | Glycine max | 19 | 42.485658 | hypothetical protein |
| GLYMA_20G095100 | NA | protein_coding | Glycine max | 20 | 33.802149 | hypothetical protein |
| GLYMA_20G095200 | 1.01E+08 | protein_coding | Glycine max | 20 | 33.827363 | hypothetical protein |
| GLYMA_20G095300 | 1.01E+08 | protein_coding | Glycine max | 20 | 33.83241 | hypothetical protein |
| GLYMA_20G095400 | NA | protein_coding | Glycine max | 20 | 33.844949 | hypothetical protein |
| GLYMA_20G095500 | 1.01E+08 | protein_coding | Glycine max | 20 | 33.851251 | hypothetical protein |
| GLYMA_20G095600 | NA | protein_coding | Glycine max | 20 | 33.856337 | hypothetical protein |
| GLYMA_20G095700 | 1.01E+08 | protein_coding | Glycine max | 20 | 33.861253 | hypothetical protein |
| GLYMA_20G095800 | 1.01E+08 | protein_coding | Glycine max | 20 | 33.873593 | hypothetical protein |
| GLYMA_20G095900 | NA | protein_coding | Glycine max | 20 | 33.888097 | hypothetical protein |
| GLYMA_20G096000 | 1E+08 | protein_coding | Glycine max | 20 | 33.891265 | hypothetical protein |
| GLYMA_20G096100 | 1.01E+08 | protein_coding | Glycine max | 20 | 33.906543 | hypothetical protein |
| GLYMA_20G096200 | NA | protein_coding | Glycine max | 20 | 33.92347 | hypothetical protein |
| GLYMA_20G096300 | NA | protein_coding | Glycine max | 20 | 33.927527 | hypothetical protein |
| GLYMA_20G096400 | NA | protein_coding | Glycine max | 20 | 33.932231 | hypothetical protein |
| GLYMA_20G096500 | NA | protein_coding | Glycine max | 20 | 33.943385 | hypothetical protein |
| GLYMA_20G096600 | NA | protein_coding | Glycine max | 20 | 33.944718 | hypothetical protein |
| GLYMA_20G096700 | 1.01E+08 | protein_coding | Glycine max | 20 | 33.954993 | hypothetical protein |
| GLYMA_20G096800 | NA | protein_coding | Glycine max | 20 | 33.996507 | hypothetical protein |
| GLYMA_20G096900 | 1.01E+08 | protein_coding | Glycine max | 20 | 34.000859 | hypothetical protein |
| GLYMA_20G097000 | NA | protein_coding | Glycine max | 20 | 34.012486 | hypothetical protein |
| GLYMA_20G097100 | NA | protein_coding | Glycine max | 20 | 34.018993 | hypothetical protein |
| GLYMA_20G097200 | 1.01E+08 | protein_coding | Glycine max | 20 | 34.025439 | hypothetical protein |
| GLYMA_20G097300 | NA | protein_coding | Glycine max | 20 | 34.028989 | hypothetical protein |
| GLYMA_20G097400 | 1.03E+08 | protein_coding | Glycine max | 20 | 34.033301 | hypothetical protein |
| GLYMA_20G097500 | 1.03E+08 | protein_coding | Glycine max | 20 | 34.047382 | hypothetical protein |
| GLYMA_20G097600 | 1.01E+08 | protein_coding | Glycine max | 20 | 34.07388 | hypothetical protein |
| GLYMA_20G097700 | 1.01E+08 | protein_coding | Glycine max | 20 | 34.078389 | hypothetical protein |
| GLYMA_20G097800 | 1.03E+08 | protein_coding | Glycine max | 20 | 34.091099 | hypothetical protein |
| GLYMA_20G097900 | NA | protein_coding | Glycine max | 20 | 34.120493 | hypothetical protein |
| GLYMA_20G098000 | NA | protein_coding | Glycine max | 20 | 34.129429 | hypothetical protein |
| GLYMA_20G098100 | 1.01E+08 | protein_coding | Glycine max | 20 | 34.134555 | hypothetical protein |
| GLYMA_20G098200 | 1.01E+08 | protein_coding | Glycine max | 20 | 34.140611 | hypothetical protein |
| GLYMA_20G098300 | 1.01E+08 | protein_coding | Glycine max | 20 | 34.143117 | hypothetical protein |
| GLYMA_20G098400 | 1.01E+08 | protein_coding | Glycine max | 20 | 34.151918 | hypothetical protein |
| GLYMA_20G098500 | NA | protein_coding | Glycine max | 20 | 34.179794 | hypothetical protein |
| GLYMA_20G098600 | NA | protein_coding | Glycine max | 20 | 34.184591 | hypothetical protein |
| GLYMA_20G098700 | 1.01E+08 | protein_coding | Glycine max | 20 | 34.192396 | hypothetical protein |
| GLYMA_20G098800 | NA | protein_coding | Glycine max | 20 | 34.20288 | hypothetical protein |
| GLYMA_20G098900 | 1.01E+08 | protein_coding | Glycine max | 20 | 34.207557 | hypothetical protein |
| GLYMA_20G099000 | NA | protein_coding | Glycine max | 20 | 34.230793 | hypothetical protein |
| GLYMA_20G099100 | 1.01E+08 | protein_coding | Glycine max | 20 | 34.238288 | hypothetical protein |
| GLYMA_20G099200 | 1.01E+08 | protein_coding | Glycine max | 20 | 34.242852 | hypothetical protein |
| GLYMA_20G099300 | 1.01E+08 | protein_coding | Glycine max | 20 | 34.253262 | hypothetical protein |
| GLYMA_20G099400 | 1.01E+08 | protein_coding | Glycine max | 20 | 34.257615 | hypothetical protein |
| GLYMA_20G099500 | NA | protein_coding | Glycine max | 20 | 34.275736 | hypothetical protein |
| GLYMA_20G099600 | NA | protein_coding | Glycine max | 20 | 34.295547 | hypothetical protein |
